# RECLU: a pipeline to discover reproducible transcriptional start sites and their alternative regulation using capped analysis of gene expression (CAGE)

**DOI:** 10.1186/1471-2164-15-269

**Published:** 2014-04-25

**Authors:** Hiroko Ohmiya, Morana Vitezic, Martin C Frith, Masayoshi Itoh, Piero Carninci, Alistair RR Forrest, Yoshihide Hayashizaki, Timo Lassmann

**Affiliations:** 1 RIKEN Center for Life Science Technologies (CLST), Division of Genomic Technologies, RIKEN Yokohama Institute, 1-7-22 Suehiro-cho, Tsurumi-ku, 230-0045 Yokohama, Japan; 2 RIKEN Advanced Center for Computing and Communication, Preventive Medicine and Applied Genomics Unit, 1-7-22 Suehiro-cho, Tsurumi-ku, 230-0045 Yokohama, Japan; 3 Department of Cell and Molecular Biology (CMB), Karolinska Institute, SE-171 77 Stockholm, Sweden; 4 Sequence Analysis Team, Computational Biology Research Center, National Institute of Advanced Industrial Science and Technology (AIST), 2-4-7 Aomi, Koto-ku, 135-0064 Tokyo, Japan; 5 RIKEN Preventive Medicine and Diagnosis Innovation Program (PMI), RIKEN Yokohama Institute, 1-7-22 Suehiro-cho, Tsurumi-ku, 230-0045 Yokohama, Japan; 6 Bioinformatics Centre, Department of Biology, University of Copenhagen, Ole Maaløes Vej 5, DK-2200 Copenhagen N, Denmark

**Keywords:** CAGE, Peak finding, Reproducibility, Hierarchical stability

## Abstract

**Background:**

Next generation sequencing based technologies are being extensively used to study transcriptomes. Among these, cap analysis of gene expression (CAGE) is specialized in detecting the most 5’ ends of RNA molecules. After mapping the sequenced reads back to a reference genome CAGE data highlights the transcriptional start sites (TSSs) and their usage at a single nucleotide resolution.

**Results:**

We propose a pipeline to group the single nucleotide TSS into larger reproducible peaks and compare their usage across biological states. Importantly, our pipeline discovers broad peaks as well as the fine structure of individual transcriptional start sites embedded within them. We assess the performance of our approach on a large CAGE datasets including 156 primary cell types and two cell lines with biological replicas. We demonstrate that genes have complicated structures of transcription initiation events. In particular, we discover that narrow peaks embedded in broader regions of transcriptional activity can be differentially used even if the larger region is not.

**Conclusions:**

By examining the reproducible fine scaled organization of TSS we can detect many differentially regulated peaks undetected by previous approaches.

## Background

The production of specific mRNAs by RNA polymerase II is regulated in most phases of homeostasis, growth, differentiation and development in eukaryotes. Measuring the transcription initiation events comprehensively will enable us to characterize aberrant expression patterns in human diseases and therefore aid in the identification of causative genes. The transcription start site (TSS) of a gene is defined by the first nucleotide that is copied at the 5’ end of the corresponding mRNA [1]. For the analysis of TSSs, sequencing-based methods have been developed prominently including the cap analysis of gene expression (CAGE) [2,3]. Mapping CAGE reads back to the genome identifies the active TSSs in a particular biological context while counting the number of reads at each site allows for quantification of the downstream RNAs. Multiple studies using CAGE technology have revealed the relationship between TSSs and core promoters, a broader region which collects multiple TSS events, the distribution of TSSs (TSSD) and transcription factor binding motifs around them, and the properties of each promoter class classified by the distribution of TSSs [1,4,5]. A challenge for the data analysis is to assemble nearby TSS into larger units representing co-regulated biological events. Frith et al. [6] demonstrated that CAGE peaks are composed of a hierarchy of overlapping peaks. The fine structure of peaks is largely determined by the local nucleotide composition of the genome, while broader regions of activity are likely to be determined by epigenetic effects [7]. To conduct the analysis, Frith et al. [6] developed a parametric clustering algorithm implemented in the program Paraclu which reports genomic intervals containing many more CAGE reads than surrounding regions. These regions can be contained within each other giving rise to a hierarchy of peaks. We used this program in the recent ENCODE study to define 82,783 transcriptional start regions in 15 cell lines [8].

However, in all previous studies only a single level of the peak hierarchy was used in the downstream analysis. For example, in the ENCODE study we collapsed the hierarchy by excluding all peaks contained within others. This is clearly unsatisfying from a biological standpoint as peaks at different scales may actually represent different events such as broad regions of open chromatin, narrower alternative promoters and finally individual TSS events.

This study was designed to understand structures of transcription initiation events using the CAGE technology. To achieve this goal we developed a pipeline discovering reproducible TSS peaks with multiple scales based on Paraclu and detect their alternative usage. We modified the original clustering approach in three ways to overcome the limitations mentioned above. First, we used the tag density as the cutoff value instead of the number of tags in a cluster to discard weakly expressed clusters and detect moderately expressed and narrow peaks. Secondly, we hypothesize that using reproducible peaks at different levels of the hierarchy is important in understanding changes in expression levels. Thus, we replaced collapsing clusters with extracting both the lowest peaks (termed “bottom”) and the highest peaks (termed “top”). Finally, the original Paraclu by Frith et al. [6] calculates a stability criterion for each cluster. In brief, Paraclu defines clusters as maximal scoring segments which are found by varying a density parameter *d*. Clusters with a low *d* are large and have sparse tags, and ones with a high *d* tend to be small and dense. Paraclu finds all possible maximal scoring segments and annotates each segment with the minimum and maximum values of *d* where it is maximal scoring. If a particular segment is maximal scoring over a large range of values for *d*, it is intuitively a “stable” cluster. Thus, the stability of each cluster is defined as max *d*/min *d*. Since we are interested in extracting reproducible clusters across replicas rather than excluding clusters with low stability, we simply added the stability of broad peaks to the ones of all peaks they contain to assess their responsibility and called it hierarchical stability. Based on these hierarchical stabilities in multiple replicates we used the irreproducible discovery rate (IDR) [9] analysis to evaluate the accuracy of each peak. The IDR analysis is used for quantitatively measuring the consistency between replicas and for selecting reproducible signals. We summarized the differences between the original Paraclu and our pipeline, RECLU in Table 1.

**Table 1:** Differences between two clustering methods

	**Original Paraclu**	**RECLU**
Threshold to discard clusters	Number of tags in the cluster	Tag per million (TPM) per base
Algorithm to calculate stabilities	Ratio of the maximum d to the minimum d	Sum of stabilities of accumulated clusters at the site
Evaluation of reproducibility	NA	IDR
Consensus cluster	Extracting the largest cluster at each site	Clusters at the top and bottom of the hierarchy

We implemented our approach as a complete analysis pipeline that firstly constructs the set of reproducible regions among related samples and in a second step detects significant changes between samples. Applying our approach to the FANTOM5 dataset [10] comprising 156 human primary, HeLa and THP-1 cells [11], whose properties are described in Additional file 1, reveals complex patterns of alternative peak usage. By including peaks at different levels in the hierarchy we can detected many additional differentially expressed alternative start sites compared to a previous approaches. We conclude that our pipeline is an effective tool to automatically discover alternative peaks and their differential usage among samples. This work is part of the FANTOM5 project. Data downloads, genomic tools and co-published manuscripts are summarized here [12].

## Implementation

**Figure 1:**
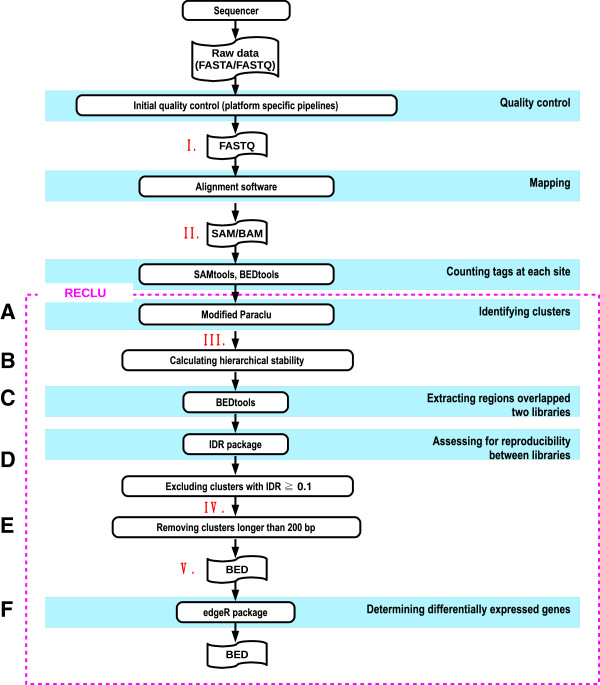
**Summary of the pipeline.** The procedures surrounded by the pink dotted line are contained in the pipeline. The Roman numerals correspond to Additional file 1. **(A)** Tag count data are clustered using the improved Paraclu program [6] (version 4). Clusters with TPM per base < 0.1 were discarded instead of using the total tag count in a cluster. **(B)** Hierarchical stability is calculated using the stability provided by Paraclu. The hierarchical stability is simply the sum of stabilities of the hierarchical clusters. **(C)** Regions that have 90% overlap between replicates are extracted by BEDtools [15] (version 2.12.0). **(D)** Executing the IDR package [9] (version 1.1) in the R language to evaluate reproducibility between replicates. The clusters with IDR ≥ 0.1 are discarded as irreproducible ones. **(E)** As well as clusters longer than 200 bp. **(F)** Detecting differentially expressed genes by the edgeR package [17] in the R language.

The RECLU pipeline starts with the analysis of previously mapped CAGE data (Figure 1, pink box). The core steps include the clustering of individual TSS using a modified version of the Paraclu algorithm, merging overlapping peaks in different replicas and applying the irreproducible discovery analysis (IDR) [9]. We will describe the key steps and materials and methods below.

### Clustering CAGE tags

One strength of CAGE technology is that tags mapped to the genome show both the location and intensity of transcription [3,5]. Therefore, we need to reflect these characteristics of the CAGE dataset, and we adopted the Paraclu program [6] for clustering the tag counts. To apply the paraclu methods to CAGE datasets the mapped reads have to be converted into the CAGE defined transcriptional start sites (CTSS) format. In brief a CTSS counts the number of CAGE reads whose mapping start at a single nucleotide in the reference genome. We used SAMtools [13] (version 0.1.18) to count the reads at each site.

### Modified paraclu

The clustering method provided by Frith et al. [6] defines TSS regions with arbitrary sizes as well as identifying peaks embedded in other peaks. To make this approach applicable to our study we modified the algorithm in two ways. Firstly, the original algorithm discarded lowly expressed peaks based on raw read numbers. Since we are interested in analyzing reproducible peaks and this filtering might discard some short clusters with a moderate tag counts density, we need to account for different sequencing depths in biological replicas. Therefore we use a normalized tag per million (TPM) [14] per base threshold and omit clusters with < 0.1 TPM per base instead of the total tag count.

Secondly, the original algorithm calculates a stability criterion for each peak defined by the ratio between the maximal and minimal clustering parameter *d* giving rise to that peak. Clusters with a stability < 2.0 are discarded. Since we are less concerned with the stability of clusters within a single sample than the reproducibility across samples, we simply add the stability of broad peaks to the stability of peaks they contain. This hierarchal stability is an effective way of including the fine structure at promoters in the downstream steps while discarding spurious intergenic signals.

### Selecting reproducible peaks

Since we perform the clustering of CAGE data independently in each replica we need to integrate these results before being able to compare different biological samples. Our goal is to find a reproducible set of peaks in each biological condition. We use two tools to obtain such a set. Firstly, we simply compare the genomic coordinates of peaks and retain those with an reciprocal overlap of over 90 percent between any two replicas using BEDtools [15] (version 2.12.0). The genomic coordinates of the retained peaks cover only the overlapping regions and extensions in an individual replica are discarded. Secondly we verify if the signal in replicated regions is reproducible using the irreproducible discovery rate (IDR) package [9] (version 1.1). We only keep clusters with an IDR < 0.1, the same threshold as used by Derrien et al. [16]. Finally we exclude all reproducible clusters longer than 200 bp from the downstream analysis.

By definition, all clusters only found in one replica are discarded by our method. However since we apply our method to replicas corresponding to different cell types independently, it is common to discover highly reproducible clusters in one cell type which are completely absent in the other.

### Data sources

We used two CAGE datasets. The first was the human CAGE data with replicates set for 156 primary cells sequenced on a HeliScope sequencer and mapped to the hg19 genome assembly in the FANTOM5 project [10]. All primary cell data and ethics application numbers are described in the FANTOM5 main paper [10]. In brief the majority of primary cell samples were purchased from commercial suppliers while the remainder were obtained through collaborating institutes from patients who provided informed consent. The other was the triplicate human CAGE dataset for HeLa and THP-1 samples sequenced on a HeliScope sequencer and mapped to the hg18 genome assembly by Kanamori-Katayama et al. [11].

### Parameters used to run the original Paraclu program

In parallel we applied the original Paraclu program (version 4) on the CAGE datasets to compare the results. Clusters with < 30 tag counts, < 2 stability, or longer than 200 bp were discarded.

### Differential expression analysis

We used the edgeR package [17] (version 2.5.3) to perform the exact test for differential expression analysis in our pipeline. Since it is known that the variance of the distribution for expression level across samples tends to exceed the mean of the distribution at considerably many loci, which is called overdispersion, and the negative binomial distribution fits to the data better than Poisson [18], the edgeR package is widely used.

### Gene ontology analysis

We interrogated the GO terms in the GOTERM_BP_FAT category using the DAVID Bioinformatics resource [19] for differentially expressed genes for all pairwise comparisons of 11 blood cells by the FANTOM5 project [10] and the comparison between the HeLa and THP-1 cells by Kanamori-Katayama et al. [11]. Firstly, we extracted highly expressed genes in a cell type compared with the other ones (up-regulated), and vice versa (down-regulated), based on the clustering by the original Paraclu and our pipeline, respectively. Next, we picked out the commonly up-regulated/down-regulated genes in both clustering methods and performed GO analysis by using these genes. We adopted GO terms with FDR < 0.05 as significant terms. Likewise, we also performed the GO analysis using differentially expressed genes identified by only our pipeline.

### Motif discovery analysis

We performed motif discovery analysis by using clusters with differential expression. First, we classified the clusters with an absolute log fold change > 2.0 into 4 groups; up-regulated (i.e., higher expression in the HeLa cells than in the THP-1 cells) at the top peaks, down-regulated at the top peaks, up-regulated at the bottom peaks, and down-regulated at the bottom peaks. Then, we extracted the top 100 clusters with the highest log concentration from each group to make target datasets, and we randomly selected 500 clusters from the sets not significantly differentially expressed as a control dataset. We used the same control dataset for all analyses. For all datasets, we use the region of ± 500 bp relative to the TSS. We executed Dispom [20] (version 1.5), which discovers de novo motifs significantly over-represented at the promoter region of target genes using a learning process to infer the parameters, 20 times for each dataset and discarded the results with a P-value > 1e-4 or those whose distance between the 75 and 25 position distribution percentile is > 50 bp. We then compared standard motif representations in the JASPAR core database [21] using the Tomtom program [22] (version 4.8.1) measuring the similarity between motifs, and extracted those with a P-value < 1.0e-4.

## Results

To evaluate our pipeline we used a large set of primary cells sequenced in biological replicates as well as a previously published dataset comparing the cell lines HeLa and THP-1 [11]. Although we focus here on the application of the pipeline to the human CAGE datasets, the pipeline can also be applied to CAGE data from other organisms.

### Hierarchical structure of TSS

**Figure 2:**
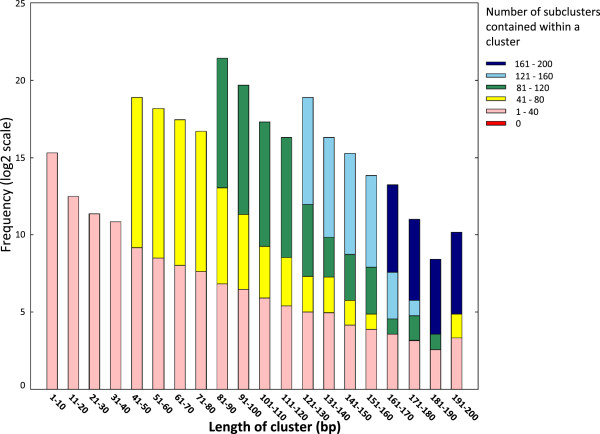
**Histogram of cluster length for HeLa sample.** Clusters with IDR ≥ 0.1 or longer than 200 bp were discarded. Clusters were binned according to their size, and the number of clusters for each size was plotted on the graph. Within each size bin, the clusters containing 1 to 40 clusters (i.e., “clusters within clusters”) are shown in pink, containing 41 to 80 clusters are yellow, and so on (see legend at right of the figure). The frequency of clusters in each size bin is represented in the log (base 2).

The main advantage of our approach is its ability to detect multiple overlapping transcriptional elements; TSS peaks with multiple scales. To explore the basic characteristics of them, we plotted the length of peaks against the number of other peaks contained within them (Figure 2). Remarkably, even relatively short peaks contain a large number of other elements indicating that CAGE defined transcriptional start regions have highly complex and hierarchical structures.

### Properties of RECLU peaks

**Figure 3:**
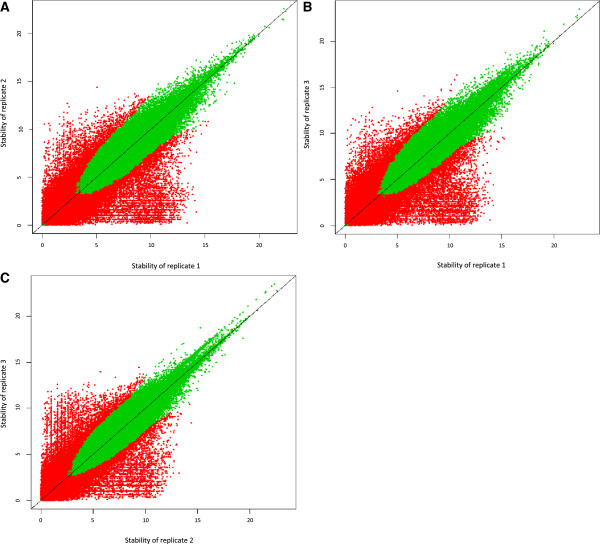
**Scatter plot of hierarchical stabilities for all pairwise combinations in HeLa cells sequenced by HeliScope.** Green and red dots indicate reproducible and irreproducible clusters at a 0.1 IDR threshold respectively. **(A)** The result between replicate 1 and 2. **(B)** Between replicate 1 and 3. **(C)** Between replicate 2 and 3.

We examined the association between the stability and the reproducibility for peaks. As expected, peaks with a high stability tend to be more reproducible than low stability peaks (Figure 3). The analysis intuitively demonstrates that hierarchical stability and reproducibility is a useful way to visualize sample quality.

**Figure 4:**
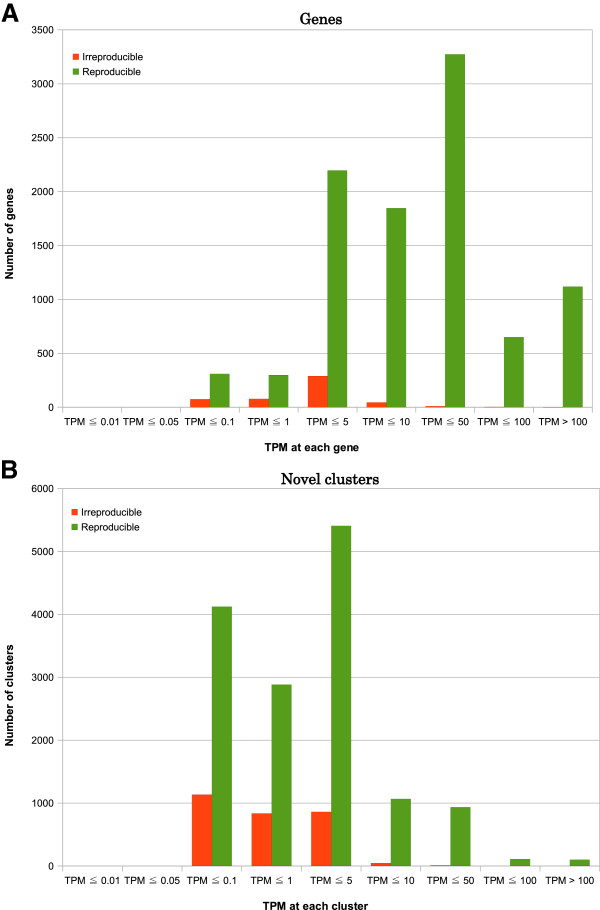
**TPM distribution for reproducible and irreproducible clusters.(A)** The number of genes on the RefSeq hg18 genome annotated by reproducible and irreproducible clusters for HeLa cells by Kanamori-Katayama et al. [11] are represented at each TPM bin. Clusters with IDR < 0.1 were identified as reproducible clusters (green bars) and the other ones were classified into irreproducible clusters (orange). Within each group, the clusters were subdivided according to their TPM and plotted at each TPM bin. **(B)** The number of clusters not annotated to genes on the RefSeq hg18 genome for HeLa cells are represented in each TPM bin. These clusters do not overlap the RefSeq hg18 transcription start sites with ± 500 bp window size. Clusters were separated by the above method, and reproducible clusters are represented in green and irreproducible ones are orange.

To understand which proportion of the data is being discarded by our approach, we separated peaks into those in the vicinity of the known RefSeq genes [23] and the remaining novel set. We then plotted the number of genes with and without a single reproducible associated peak classified separated into several bins of expression (Figure 4A). Discarded genes are commonly associated with lowly expressed peaks as compared to the reproducibly detected genes. Nevertheless, an appreciable fraction of lowly expressed genes is found to be reproducible. Novel peaks tended to follow the same trend but are generally more weakly expressed than peaks associated to known genes (Figure 4B).

### Comparison to previous approach

**Figure 5:**
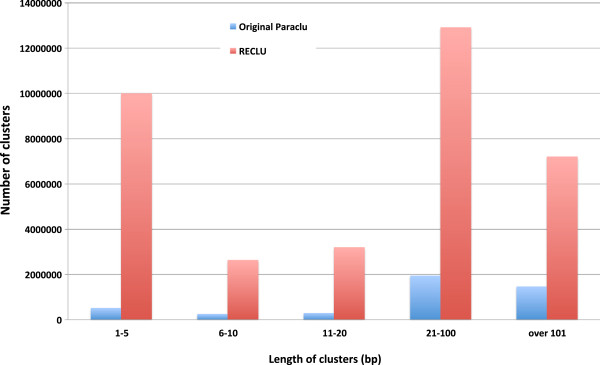
**Histogram of cluster length identified by each method.** We made the histogram of cluster length for 156 human primary cells by the original Paraclu and RECLU. For the clustering by the original Paraclu, we observed clusters for each replicate and calculated the mean length of the cell sample. We executed RECLU for all pairwise combinations of replicates in each cell sample and computed the mean.

To compare our peaks to those derived by the original algorithm we clustered data from 156 human primary cell types. Not surprisingly given the modifications we made our method discovered many more peaks than the original Paraclu (Figure 5). In particular, RECLU detected many additional clusters with a length of less than 5 bp. This indicates that RECLU detects TSSs more precisely due to using the density of CAGE tags instead of the raw tag counts.

At first we were concerned that the RECLU program would have a dramatically increased running time compared with the original Paraclu. However, the running time did not increase remarkably even when using RECLU for the datasets with different TPM thresholds (Additional file 2).

### Detecting differentially expressed peaks

We sought to understand whether differential regulation happens only at the scale of whole promoters or whether individual TSS positions may exhibit state specific behavior. Furthermore we can easily imagine that changes on the latter fine scale are obfuscated when defining promoters as broad regions. In addition we wanted to explore whether the additional peaks found by our method have biological relevance.

To address these points we extracted two types of clusters from a structure of clusters. The first class contains all peaks which are not themselves included in any other peak. The second one contains all peaks which do not contain any other peaks. We termed the first class as “bottom” since we can imagine these peaks as a foundation. Conversely we termed the second class as “top” peaks since these are the highest up in our collection of peaks. In essence these two classes represent the broadest and narrowest reproducible peaks we can derive from the data by applying our method.

**Figure 6:**
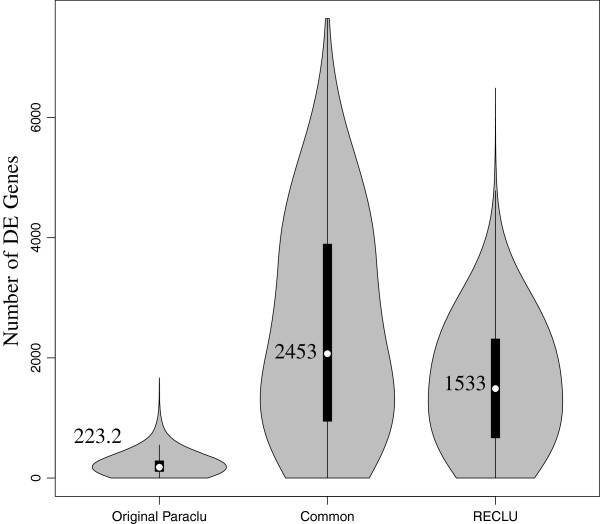
**Number of differentially expressed genes discovered in all pairwise comparisons of primary cells.** The results were separated into the common set of genes detected by both methods and the genes only detected by RECLU and the original Paraclu. The numbers in the plot are the mean number of genes detected in each set.

Using both classes we conducted a differential gene expression analysis comparing the HeLa and THP-1 samples (Additional file 3 and 4) and all human primary samples against each other using the edgeR package [17]. We call a gene as differentially expressed if its promoter contains at least one significantly differentially expressed top or bottom cluster (absolute log fold change > 2 and adjusted P-value < 0.05). For comparison purposes we also used the peaks produced by the original Paraclu method. On average 2453 genes are detected as differentially expressed by both methods. In addition, RECLU detected an additional 1533 genes while there are only 223 genes unique to the original Paraclu (Figure 6).

**Figure 7:**
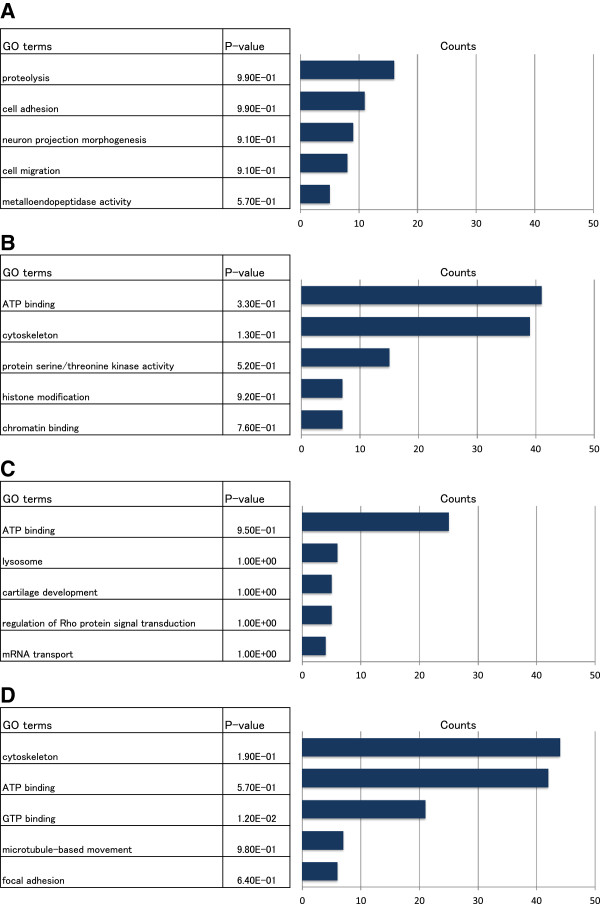
**Gene ontology terms obtained by the original Paraclu and RECLU.** We retrieved gene ontology (GO) terms describing differentially expressed genes between the HeLa and THP-1 cells after clustering by the original Paraclu or RECLU. **(A)** GO terms annotated to abundantly expressed genes for the HeLa cells compared with the THP-1 cells identified by both the original Paraclu and RECLU. **(B)** GO terms characterizing more expressed for the HeLa cells than the THP-1 cells identified by only RECLU. **(C)** GO terms annotated to strongly expressed genes for the THP-1 cells by both methods. **(D)** GO terms describing highly expressed genes for the THP-1 cells by only RECLU.

While a deep analysis of the differentially expressed genes is beyond the scope of this manuscript, we sought to understand whether the novel differentially expressed genes discovered by RECLU are of biological relevance. To address this question we performed gene ontology enrichment analysis using DAVID [19] on a subset of 11 haematopoetic primary cells. We obtained GO terms significantly enriched in the intersection of both methods and asked whether the additional genes found belong to those terms (Additional file 5). Some genes detected by only RECLU associated with GO terms by both methods, implying that RECLU could increase statistical power to detect terms identified previously. To delve further into the result, we compared GO terms detected by only RECLU, or RECLU and the original Paraclu using the HeLa and THP-1 cells (Figure 7). Terms uncovered using only RECLU tended to have lower P-values than ones using both methods across the comparison. In addition, the results obtained solely by peaks unique to RECLU shared some similar terms to those obtained by the both methods, suggesting that the additional genes belong to the same biological mechanisms. Interestingly, RECLU discovered the “ATP binding” term in the both the up-regulated and down-regulated gene groups. Finding this term in the up-regulated gene group is consistent with the fact that the exocytosis of ATP followed by activation of P2 receptors played a key role in cancer cell migration [24], and the same term in the down-regulated group is compatible with the fact that macrophages have the ability of cholesterol efflux, which is an important mechanism to maintain cholesterol homeostasis in macrophages, involving ATP-binding cassette transporter protein [25]. Thereby, it was apparent that different genes characterized by the ATP-binding term in the two different categories involved separate cell functions.

**Figure 8:**
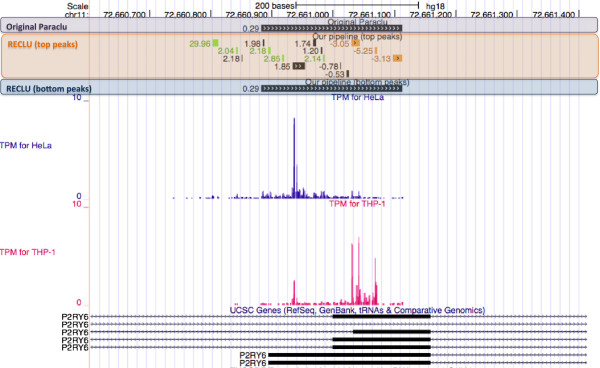
**Example of differentially expressed clusters for the comparison between the HeLa and THP-1 cells.** An Example of differentially expressed clusters was represented by the UCSC Genome Browser [36] provided by the original Paraclu and RECLU. Clusters annotated to P2RY6 gene in chromosome 11 on the RefSeq hg18 genome. Each stacked track shows consistent genomic features. The track at the top shows the location of these clusters. Below this, the next track in purple shows the peaks annotated to P2RY6 identified the original Paraclu. A black cluster indicates that the cluster is not significantly differentially expressed or < 2.0 absolute log fold change. The third track from the top emphasized in orange indicates the 15 top peaks annotated to P2RY6. Three down-regulated peaks, highly expressed peaks for the THP-1 cells compared with the HeLa cells, of them are represented in orange and the log fold changes at the left of these clusters are negative values. Green bars indicate up-regulated clusters. Likewise, the bottom peak around the transcription start sites of P2RY6 emphasized in blue is shown below. Since the peak was not significantly differentially expressed or < 2.0 absolute log fold change, the bar with chevrons is represented in black. TPM for HeLa and TPM for THP-1 tracks below this show the mean TPM among replicates at each site for HeLa and THP-1 cells, respectively. The following tracks indicate genomic information for P2RY6 based on the UCSC database [23].

For illustrative purposes we selected the peaks surrounding the P2RY6 gene (Figure 8). We observed an unchanging peak overlapping the known transcripts using the original Paraclu, which was similar to a bottom peak by our pipeline. However, several top peaks within these regions identified by our pipeline are differentially expressed between HeLa and THP-1 cells. In this case it is clear that while the overall RNA output at the gene level did not change, the main TSS of the P2RY6 gene in THP-1 cells was shifted by around 100 bp downstream relative to HeLa cells.

### Motif discovery analysis

**Figure 9:**
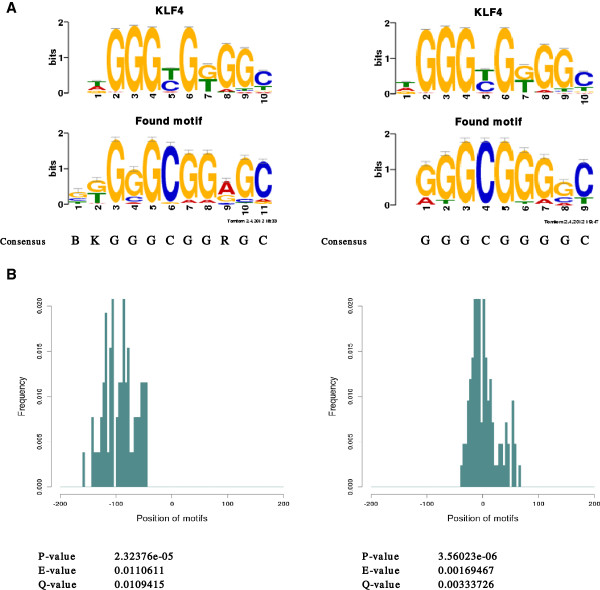
**Motif and position distribution detected in the down-regulated clusters.** The top 100 clusters having the highest log concentration and down-regulated (i.e., the HeLa cells expressed higher levels than the THP-1 cells) at the top and bottom peaks were used for motif discovery analysis. **(A)** We found differentially abundant motifs by Dispom [20], and we compared standard motif representations in the JASPAR core database [21] using the Tomtom program [22]. The sequence logo and the corresponding consensus sequence at the left-hand side were predicted from the clusters at the top peaks, and the right-hand side shows the result at the bottom peaks. The sequence logos at the top left and right sides show the KLF4 motif, and the ones below this are obtained through predictions. For the consensus sequences, B stands for C, G, or T, K stands for G or T, and R stands for A or G. **(B)** Histograms of the predicted positions relative to TSSs of the motifs at each cluster. As well as **(A)**, the figure at the left side shows the result at the top peaks, and right side is the plot at the bottom peaks.

We next performed motif discovery analysis on the peaks identified by the differential expression analysis to find transcription factor binding sites. To discover motifs based on similarity of gene expression profiles, which is the most widely used strategy [26], we separated the clusters with significantly differential expression into 4 groups and found the Kruppel-like factor 4 (KLF4) motif in promoters more highly expressed in HeLa when compared to THP-1. This was observed for both top or bottom peaks that were differentially expressed (Figure 9). Although the consensus sequences at the bottom peaks were 2 nt shorter than those at the top peaks, they were highly similar. We conclude that both top and bottom peaks could be used for motif discovery. According to the Q-value for the similarity to the KLF4 motif, the results at the bottom peaks were more significant than those at the top peaks. The position distribution of the motif was concentrated in the region of -200 ∼ + 100 bp.

Although the position distribution at the bottom peaks shifted slightly downstream compared with that at the top peaks, this distribution actually corresponds to that of the top peaks because the bottom peaks are longer than the top peaks and the TSSs at the bottom peaks are considered upstream relative to the ones at the top peaks.

Consequently, the KLF4 motif position relative to the TSS was predicted at approximately -100 bp, which is consistent with the result of Wang et al. [27]. We also investigated the differential expression of the KLF4 gene, and it was found that this gene was significantly down-regulated, matching our de novo motif discovery results; the gene had higher expression in the HeLa cells. The KLF4 gene is known as one of the transcription factors, including Oct3/4, Sox2 and c-Myc, for induction of pluripotent stem cells [28]. However, recent studies have shown that this gene could function as both an oncogene and a suppressor of p53 expression by acting directly on its promoter [29]. Another study reported that RNA polymerase III transcription factor TFIIIC had a region with significant similarity to the KLF4 motif [30]. Given the properties of the HeLa cells described by Macville et al. [31], the finding that the expression of the KLF4 gene in the HeLa cells was higher than that of the THP-1 cells and the binding site of KLF4 was identified significantly for the genes having higher expression in the HeLa cells, makes sense. We found several genes, including lysophosphatidic acid receptor 5 (LPAR5) and telomerase RNA component (Terc), with the KLF4 motif and higher expression in the HeLa cells. The LPAR5 gene encodes a protein of a G protein-coupled receptor that binds the lipid signaling molecule lysophosphatidic acid (LPA) [32], and it has been shown that LPA stimulates cell proliferation by acting on its cognate G protein-coupled receptors and that aberrant LPA production contributes to cancer initiation [33]. The findings of Wang et al. [34] suggest that KLF4 might be responsible for reactivating Terc, hence the results in this study are in agreement with the previous studies.

## Discussion

The objective of this study was to develop a clustering pipeline producing reproducible peaks at multiple scales to understand the fine structures of transcription initiation events. We modified the clustering algorithm by Frith et al. [6] in the following way. Firstly, we adopted the tag density for a threshold instead of the raw tag counts to identify narrow peaks. As a result, RECLU detected numerous clusters with from one to five bp length (Figure 5), and the original Paraclu identified a cluster whereas RECLU discovered a lot of peaks in the same region (Figure 8), implying that RECLU can identify individual TSSs in a promoter region that are missed by the original Paraclu program. Secondly, we used two classes of clusters; the top and bottom peaks, for the downstream analyses to interpret properties of clusters with multiple scales. Consequently, we found that the structure of clusters was highly complex and hierarchic (Figure 2). An illustrative example is the P2RY6 promoter (Figure 8). While the overall expression level is similar between THP1 and HeLa cells there is a clear shift in the fine scale TSS usage. The latter is undetected by the original paraclu algorithm. Therefore, our hypothesis about the importance of considering the hierarchy of clusters was demonstrated. In addition, we showed the hierarchy and complexity of promoter regions by investigating the structure of TSSs (Additional file 4). Finally, we used IDR analysis to assess the reproducibility for each cluster based on the hierarchical stability. Here the stability is defined as the ratio between the maximum and minimum density parameter in the cluster [6], in other words it is the slope of change. Since we are interested in the entire strength of the structure of clusters rather than stability of individual clusters, we added stabilities of the broader clusters to the stability of the cluster which they contained and called it hierarchical stability. Since IDR was designed to assess quantitatively reproducibility across replicates and permit an arbitrary scale, the method can be used for a variety of experimental datasets [9]. In our pipeline the IDR analysis tends to evaluate clusters with high hierarchical stability as reproducible (Figure 3). In addition, many lowly expressed peaks are found to be reproducible (Figure 4) highlighting that the IDR analysis does not simply discard lowly expressed clusters. As a result of these improvements, we discovered many additional significantly differentially expressed genes compared to those found by the original Paraclu method. We demonstrate that additional peaks discovered by RECLU can improve downstream analyses including GO term enrichment analysis. On the basis of these results, we conclude that RECLU is well suited to detect the complex structures of transcriptional initiation events.

Different methods to identify and classify TSSs measured by CAGE have been developed [1,5,35]. However, these studies have only used non-overlapping peaks. RECLU detects overlapping peaks and detects alternative TSSs usage at a fine scale (Figure 6, and 8). This is important when moving to downstream analyses including the analysis of transcriptional regulatory networks and GO term enrichment analysis (Figure 7, Additional file 5). We have not yet examined the association between transcription initiation events and underlying genomic sequence features in depth. Doing so may reveal the combinations of DNA bindings motifs, and thereby transcription factors, gives rise to specific architectures of RECLU peaks present at individual genes.

Finally, and in contrast to previous approaches, RECLU considers the biological reproducibility to define peaks boundaries. Clinical studies, including the evaluation of response to treatment, discovering prognostic factors for early diagnosis and biomarker development to determine therapeutic strategies, will require robust analyses using highly reproducible expression peaks due to the necessity of accurate identification. Thus, assessment of reproducibility of clusters is crucial, and RECLU can be applied in clinical settings.

## Conclusions

To understand the manner and mechanisms of transcription initiation by RNA polymerase II, we propose a clustering and quality control pipeline to detect TSSs on a genome-wide scale from the CAGE sequence tags. The new framework clusters CAGE data at multiple scales and adopts the IDR to measure reproducibility between replicates of each cluster. Our pipeline reveals that genes have complicated structures of transcription initiation events and discovers novel peaks which were difficult to detect by previous approaches. We demonstrate that the additional peaks are differentially used among primary cells. Further work is needed to understand the biological role of these additional events.

## Availability and requirements

**Project name:** RECLU**Project home page:**http://fantom.gsc.riken.jp/5/**Operating systems:** Unix/Linux or Mac**Programming language:** C++, R, Perl, and bash**Other requirements:** R, SAMtools, and BEDtools.**License:** GNU GPL3**Others:** The RECLU package is available from the FANTOM web-page (http://fantom.gsc.riken.jp/software/) and sourceforge (http://en.sourceforge.jp/projects/reclu/releases/). The analysis results of this study are available via a UCSC Genome Browser track hub (http://fantom.gsc.riken.jp/5/suppl/Ohmiya_et_al_2014/data/hub.txt).

## Abbreviations

TSS: Transcription start site; CAGE: Cap analysis of gene expression; TSSD: Transcription start site distribution; IDR: Irreproducible discovery rate; TPM: Tags per million; CTSS: CAGE transcription start site; GO: Gene ontology; LINE: Long interspersed nuclear element; SINE: Short interspersed nuclear element; LTR: Long terminal repeat elements; lincRNA: Long intergenic non-coding RNA; miRNA: Micro RNA; snRNA: Small nuclear RNA; lncRNA: Long non-coding RNA.

## Competing interests

The authors declare that they have no competing interests.

## Authors’ contributions

MF developed the original clustering method and adapted it to FANTOM5-scale data. MV participated in the design of this study and performed a part of statistical analyses. MI was responsible for CAGE data production. HO and TL carried out the analysis and drafted the manuscript. TL designed this study. YH and TL supervised the study. PC, ARRF and YH were responsible for the FANTOM consortium management and concept. The FANTOM consortium supported the design of this study. All authors read and approved the final manuscript.

## Supplementary Material

Additional file 1 **Tag counts at phases of the pipeline for each dataset.** The numbers at the left side correspond to Figure 1. FANTOM5 project dataset is human CAGE data for a variety of primary cells as described in Forrest et al. [10]. The other dataset described by Kanamori-Katayama et al. [11] is triplicate human CAGE data for HeLa and THP-1 cells. The former dataset was mapped to the hg19 genome, and the other one was the hg18 genome. Both datasets were sequenced by the HeliScope platform. Click here for file

Additional file 2 **Running time of the original Paraclu and RECLU program.** Elapsed times (sec) of the original Paraclu and RECLU programs with different parameter values for each replicate in HeLa and THP-1 cells are shown in the line plots. **(A)** The elapsed times of the original Paraclu program with different total tag counts (1, 10, 20, 30 and 40) as a threshold are shown. **(B)** We improved the original Paraclu program to eliminate clusters with < 0.1 tags per million (TPM) instead of the total tag counts, measured the running time of it with different TPMs. Click here for file

Additional file 3 **Examples of the clusters with complicated structures.** The distributions of clusters and TPMs for HeLa and THP-1 cells provided by Kanamori-Katayama et al. [11] are shown by the UCSC Genome Browser [36]. **(A)** Clusters annotated to TXNDC12 gene in chromosome 1 on the RefSeq hg18 genomes. The track at the top shows the location of these clusters. Below this, the next track indicates the five top peaks located around the transcription start sites of the gene. The one up-regulated peak of them is represented in green and the log fold change at the left of the cluster is more than 2.0, and the other top peaks in black are not significantly differentially expressed and the log fold change is 0.0. Likewise, the two bottom peaks are shown below. Since the peak at the left side is now significantly differentially expressed, the cluster is represented in black, while the other one is down-regulated and the cluster is orange and the log fold change is less than -2.0. “TPM for HeLa” and “TPM for THP-1” tracks show the mean TPM among replicates at each site for HeLa and THP-1 cells, respectively. The following tracks indicate genomic information for TXNDC12 based on the UCSC Genome Browser database. The two hierarchical cluster collections are annotated to TXNDC12, and the one of them is highly expressed for THP-1 cells than HeLa cells, while the other one has higher expression for HeLa cells. **(B)** Clusters annotated to JMJD5 gene is chromosome 16 on the RefSeq hg18 genome. For the details at each track, see the above description at **(A)**. **(C)** Clusters annotated to C14orf1 gene in chromosome 14. There are two hierarchical cluster chunks and one of them is down-regulated and the other one is up-regulated. Click here for file

Additional file 4 **Summary of results for differential expression analysis.(A)** The number of genes on the RefSeq hg18 genome annotated by clusters significantly differentially expressed between HeLa and THP-1 cells by Kanamori-Katayama et al. [11]. The differential expression analysis was executed for the top peaks and bottom peaks separately by the edgeR package [17] in the R language, and significantly differentially expressed clusters (adjusted P-value <0.05) with > absolute log fold change were annotated to genes. **(B)** The number of clusters not annotated to genes on the RefSeq hg18 genome with significantly differentially expressed between HeLa and THP-1 cells. These clusters do not overlap the RefSeq hg18 transcription start sites with ± 500 bp windowsize. **(C)** The clusters annotated to genes. The blue bar at the leftmost site represents the number of genes with only one differentially expressed cluster, and the next blue bar corresponds to the number of genes with two differentially expressed clusters. The red bar at the leftmost site represents the total number of clusters annotated to the genes with only one differentially expressed cluster. The red bar at the rightmost site indicates the total number of clusters annotated to the genes with over 10 differentially expressed clusters. Click here for file

Additional file 5 **GO terms describing differentially expressed genes.** Gene ontology (GO) terms characterizing genes and the number of differentially expressed genes. The left most column shows GO terms (FDR < 0.05) that we interrogated for the differentially expressed genes identified by both the original Paraclu and our pipeline, and the next columns are the FDR and the number of genes characterized by the term, respectively. The other columns are the number of differentially expressed genes annotated to the terms and identified by RECLU or the original Paraclu. The low at the bottom of each table represents the sum of above values. Click here for file
