# Supplementary figures and images for: RECLU: a pipeline to discover reproducible transcriptional start sites and their alternative regulation using capped analysis of gene expression (CAGE)
Source: BMC Genomics. 2014 Apr 25;15:269. doi: 10.1186/1471-2164-15-269 (PMC4029093; doi:10.1186/1471-2164-15-269)

**A**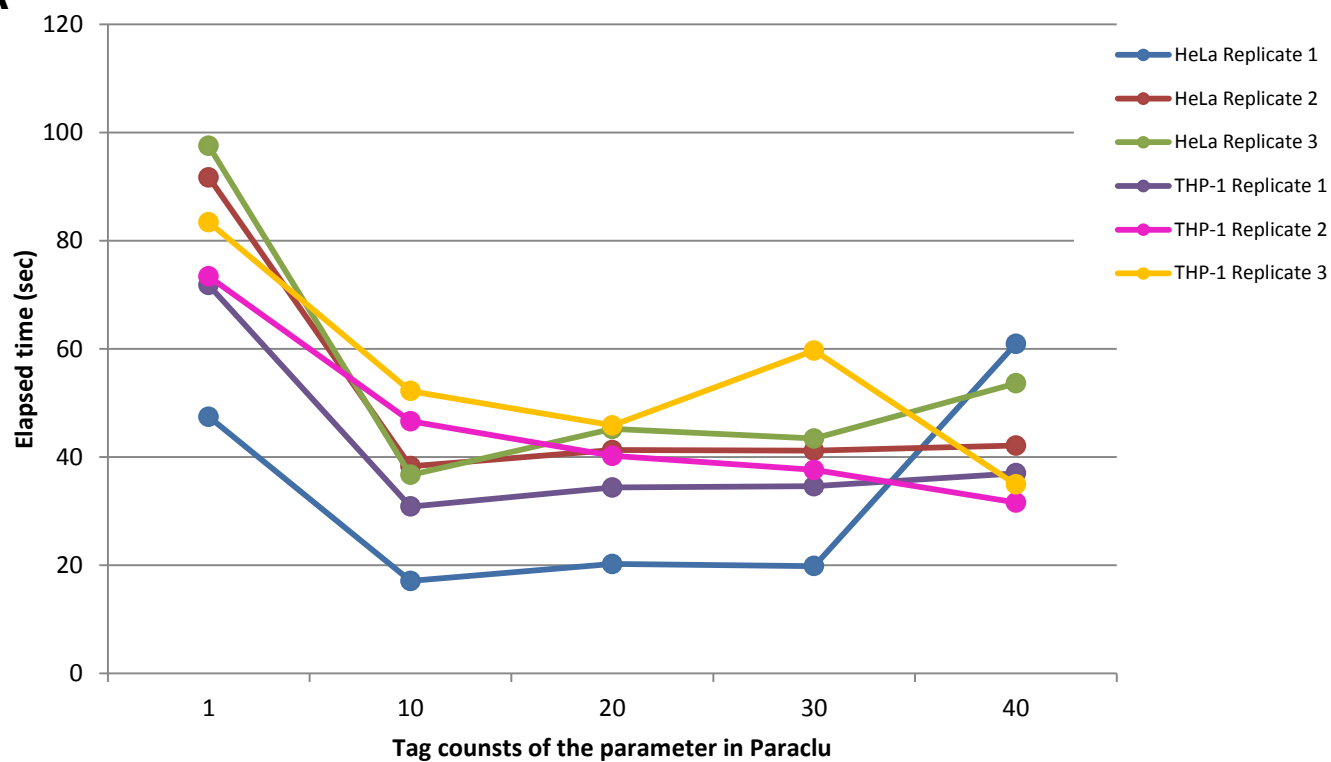**B**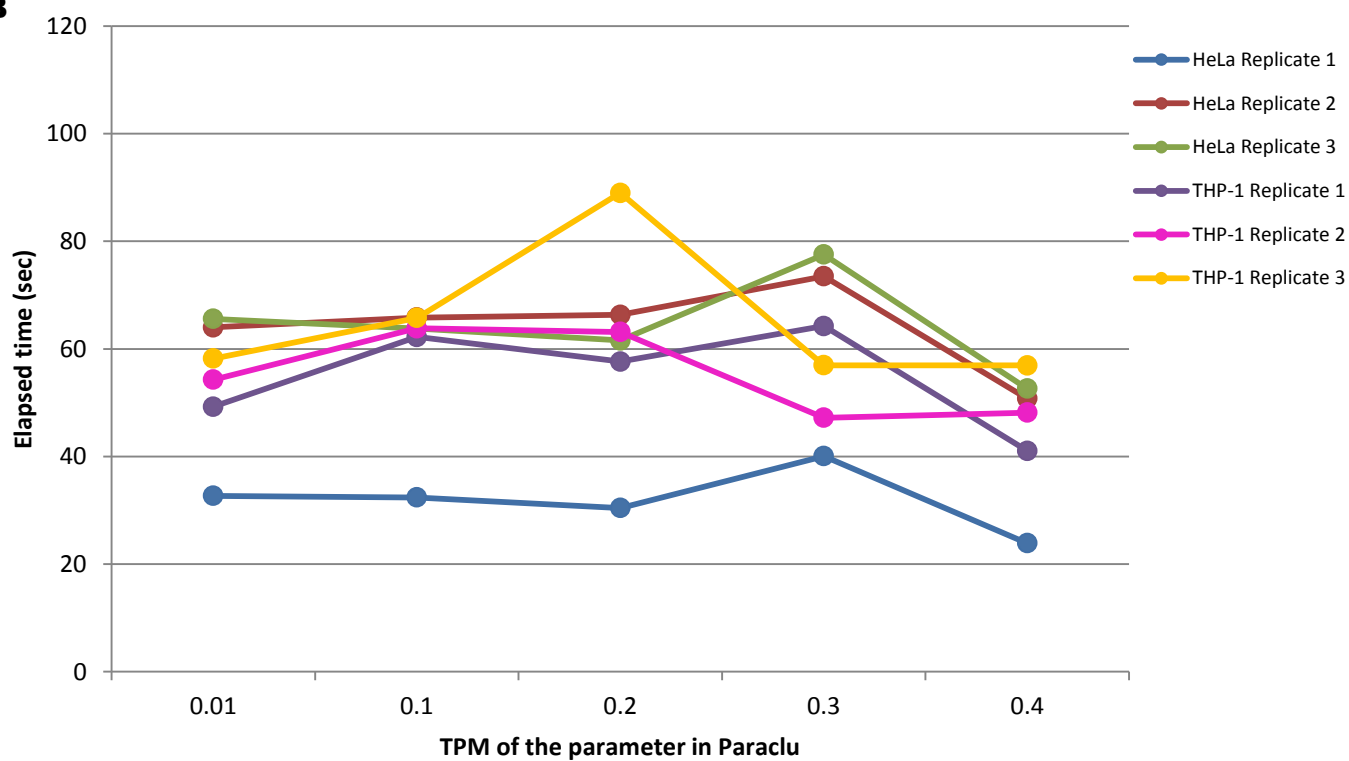

Supplement: Additional file 2 — Running time of the original Paraclu and RECLU program. Elapsed times (sec) of the original Paraclu and RECLU programs with different parameter values for each replicate in HeLa and THP-1 cells are shown in the line plots. (A) The elapsed times of the original Paraclu program with different total tag counts (1, 10, 20, 30 and 40) as a threshold are shown. (B) We improved the original Paraclu program to eliminate clusters with < 0.1 tags per million (TPM) instead of the total tag counts, measured the running time of it with different TPMs. [file 1471-2164-15-269-S2.PDF]

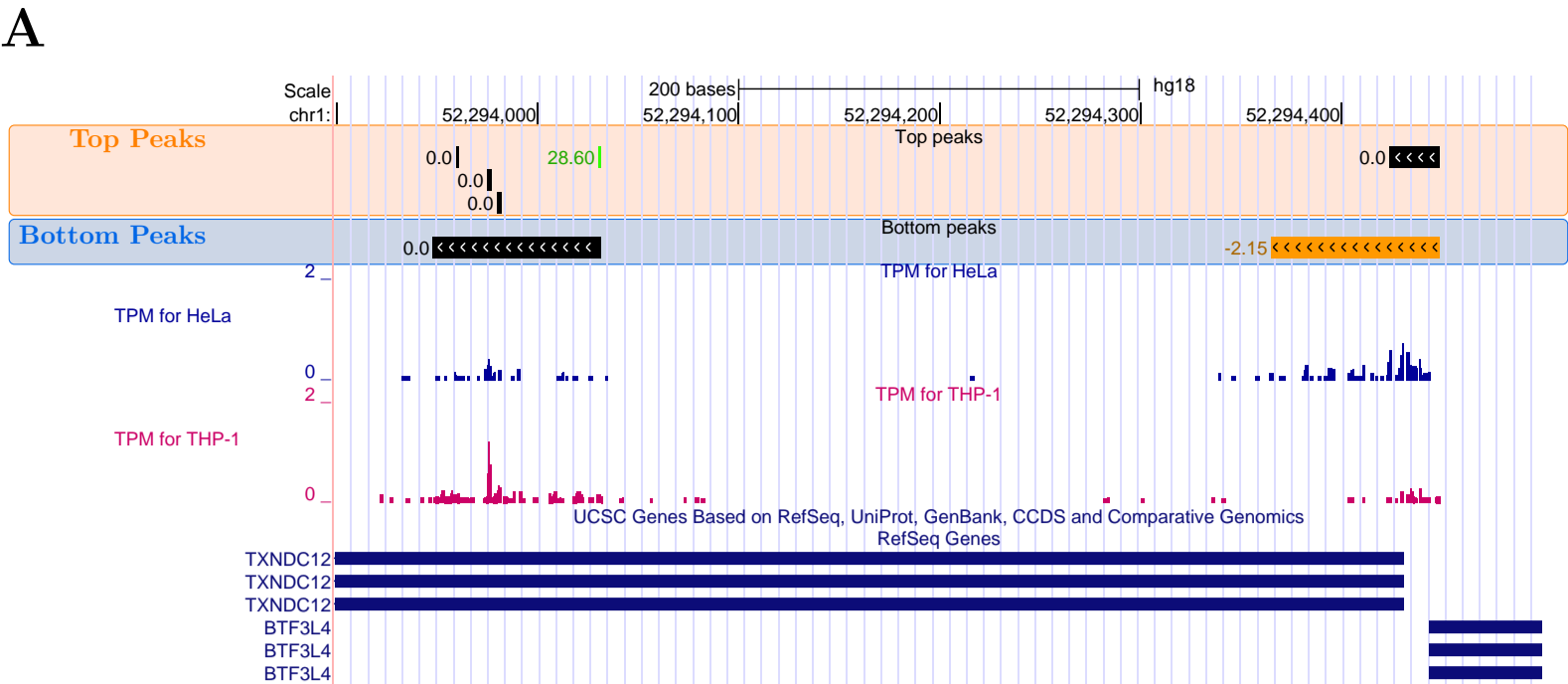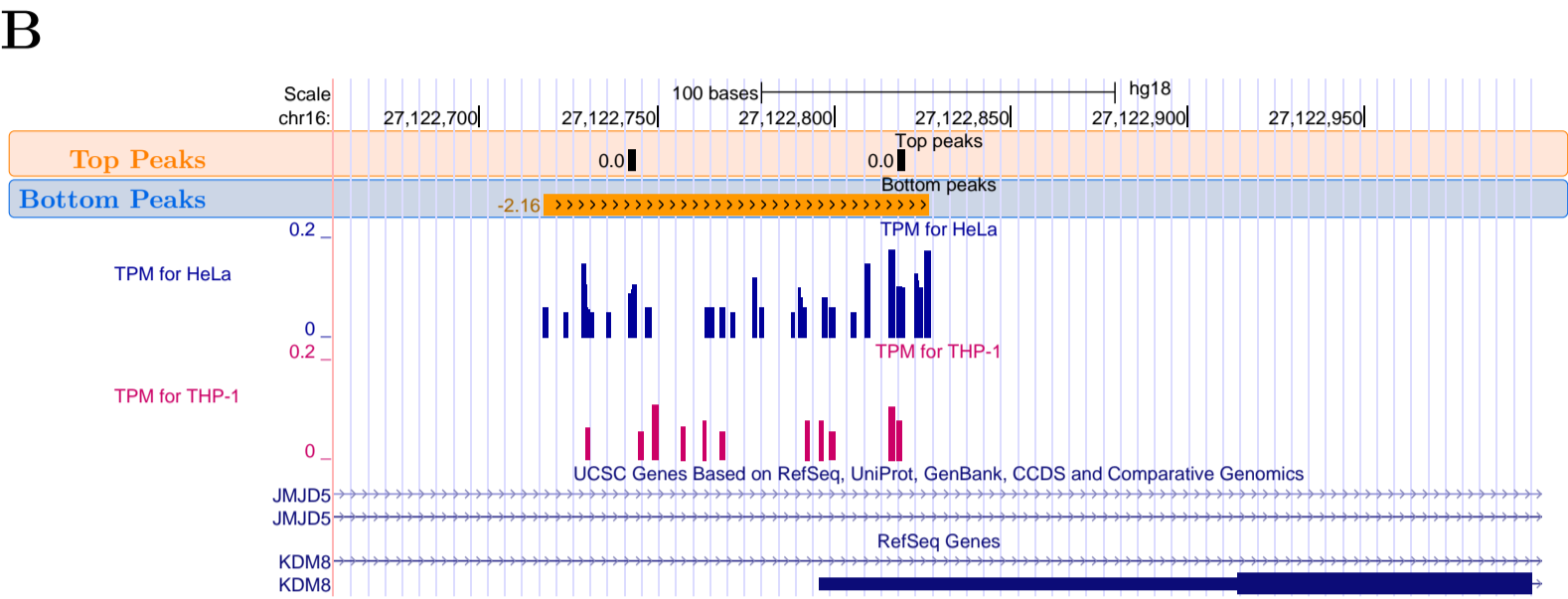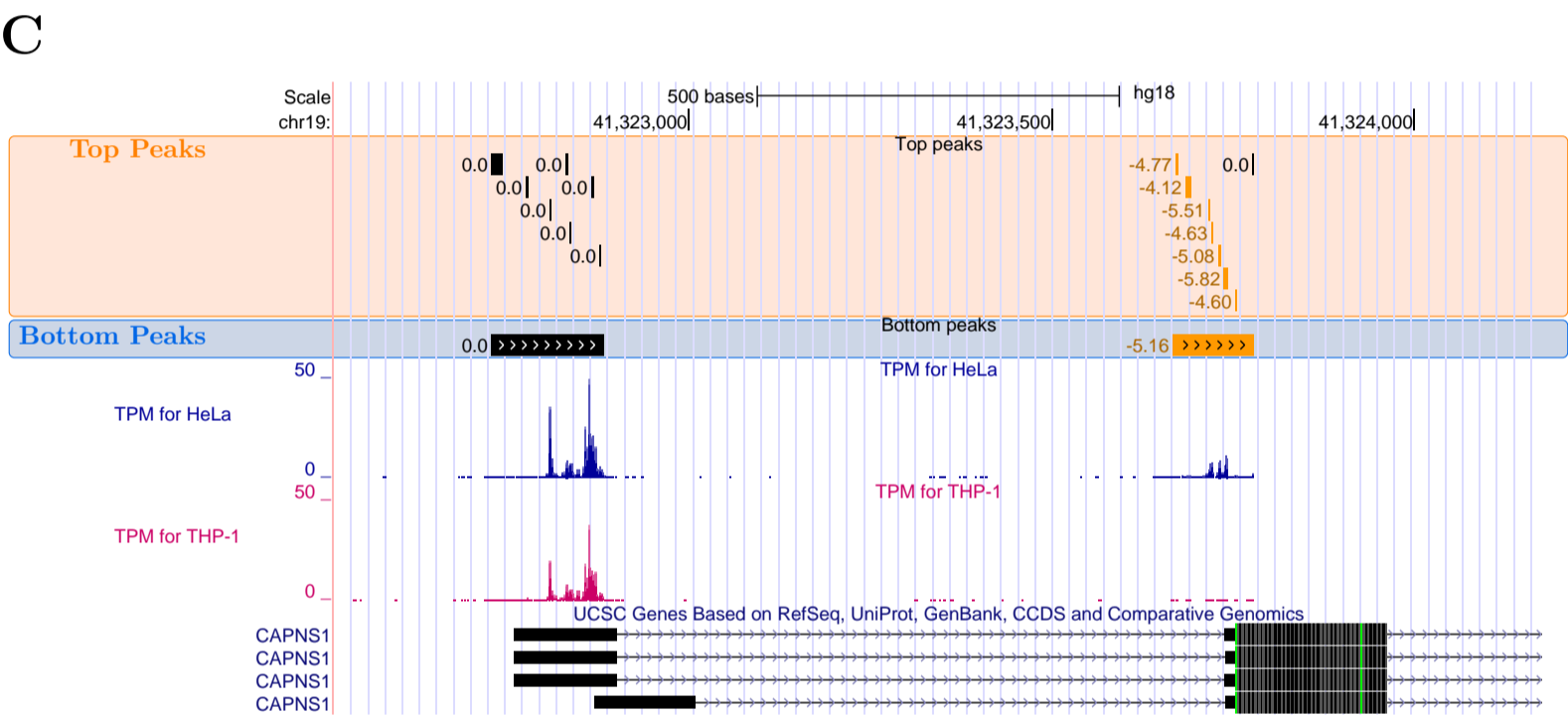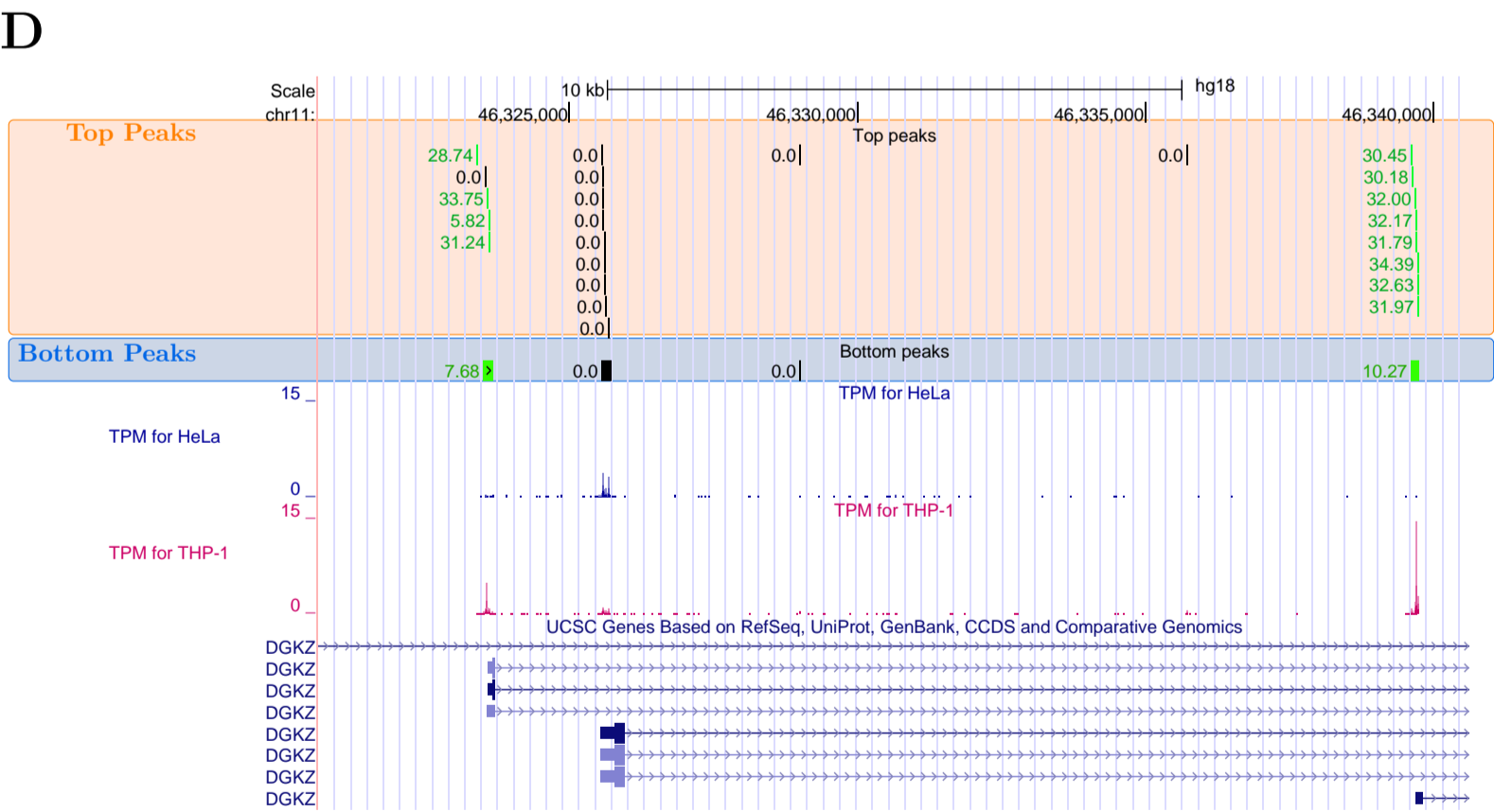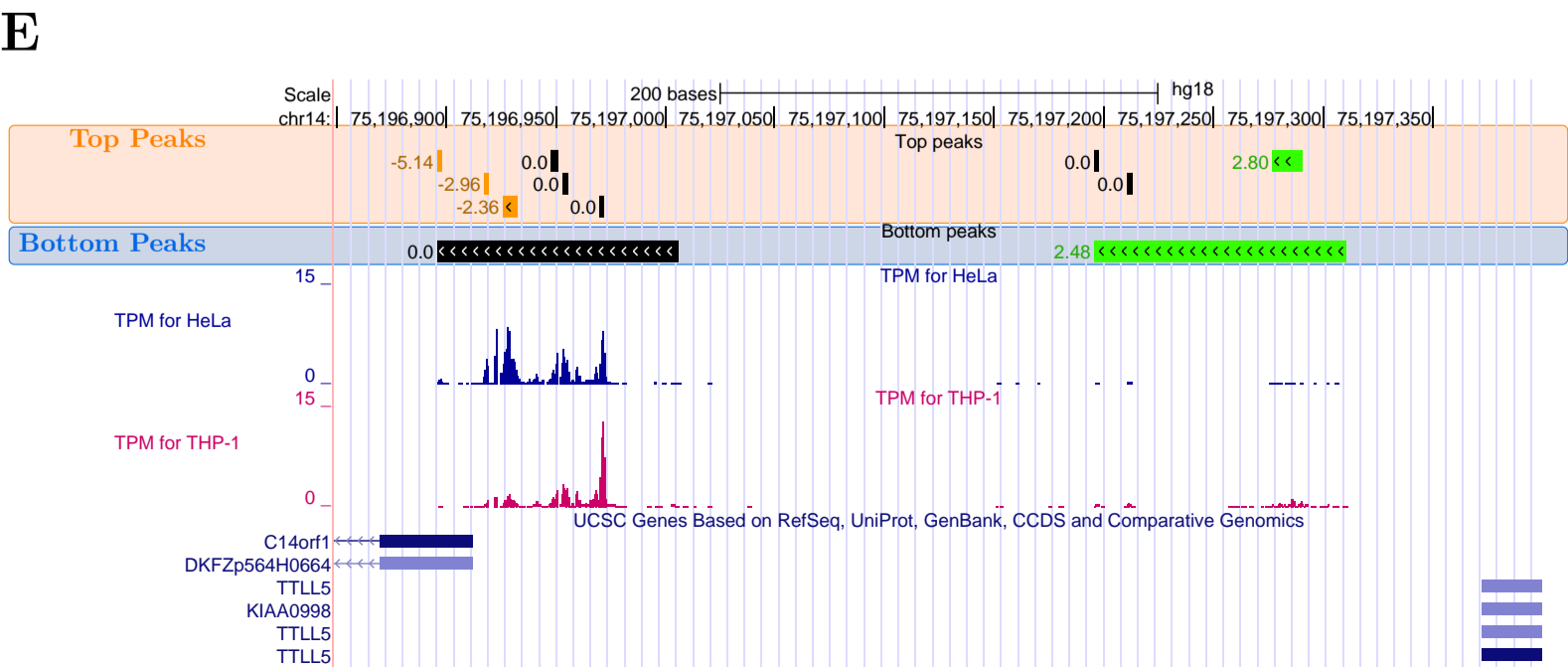

Supplement: Additional file 4 — Summary of results for differential expression analysis.(A) The number of genes on the RefSeq hg18 genome annotated by clusters significantly differentially expressed between HeLa and THP-1 cells by Kanamori-Katayama et al. [11]. The differential expression analysis was executed for the top peaks and bottom peaks separately by the edgeR package [17] in the R language, and significantly differentially expressed clusters (adjusted P-value <0.05) with > absolute log fold change were annotated to genes. (B) The number of clusters not annotated to genes on the RefSeq hg18 genome with significantly differentially expressed between HeLa and THP-1 cells. These clusters do not overlap the RefSeq hg18 transcription start sites with ± 500 bp windowsize. (C) The clusters annotated to genes. The blue bar at the leftmost site represents the number of genes with only one differentially expressed cluster, and the next blue bar corresponds to the number of genes with two differentially expressed clusters. The red bar at the leftmost site represents the total number of clusters annotated to the genes with only one differentially expressed cluster. The red bar at the rightmost site indicates the total number of clusters annotated to the genes with over 10 differentially expressed clusters. [file 1471-2164-15-269-S4.PDF]

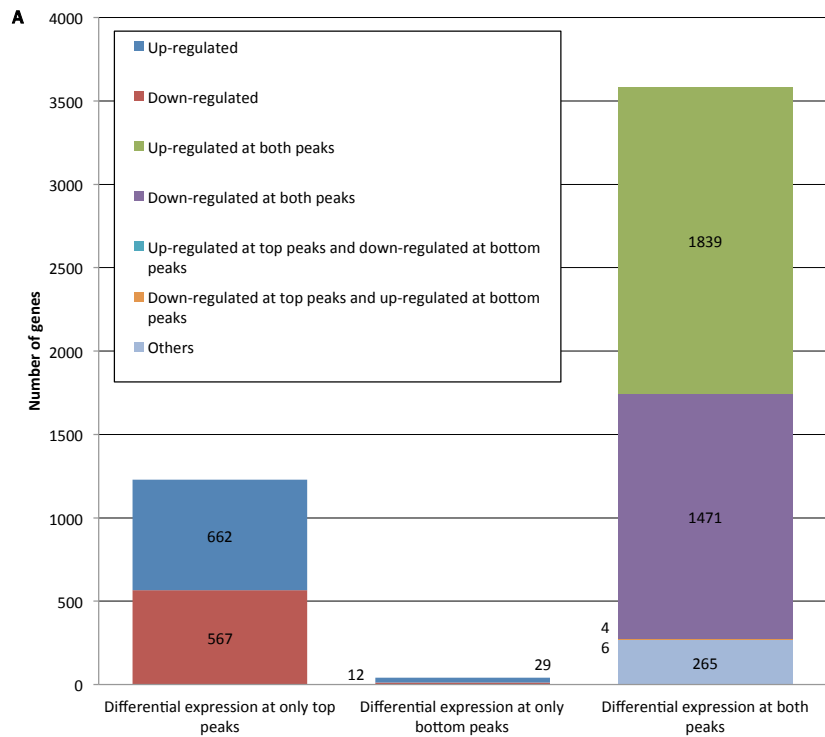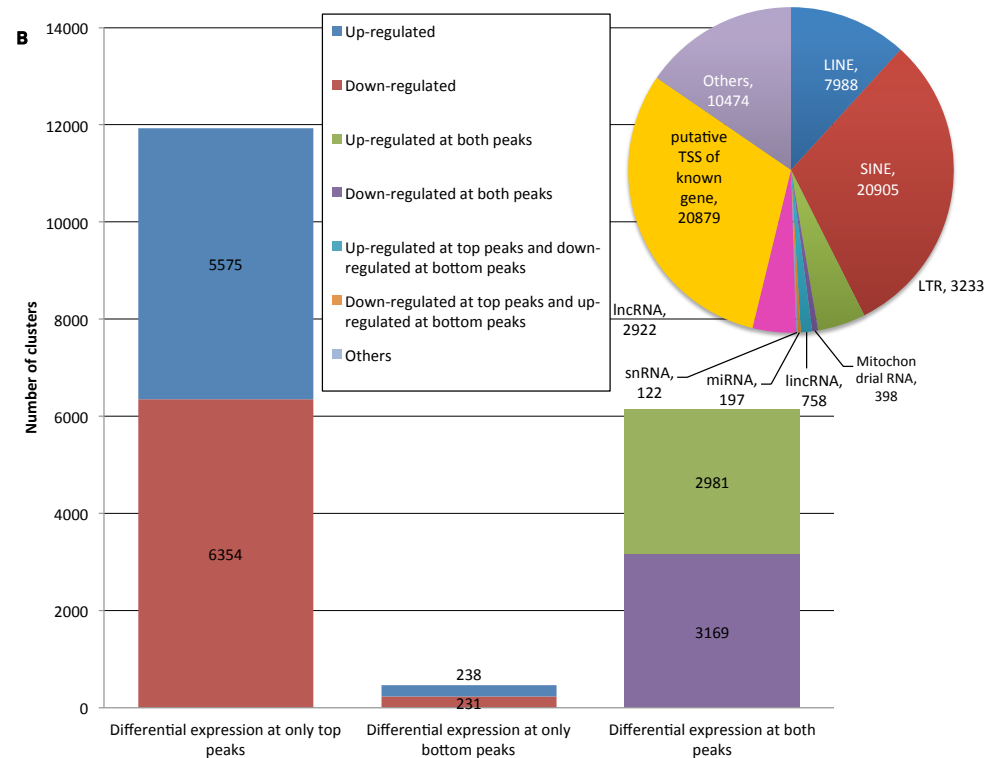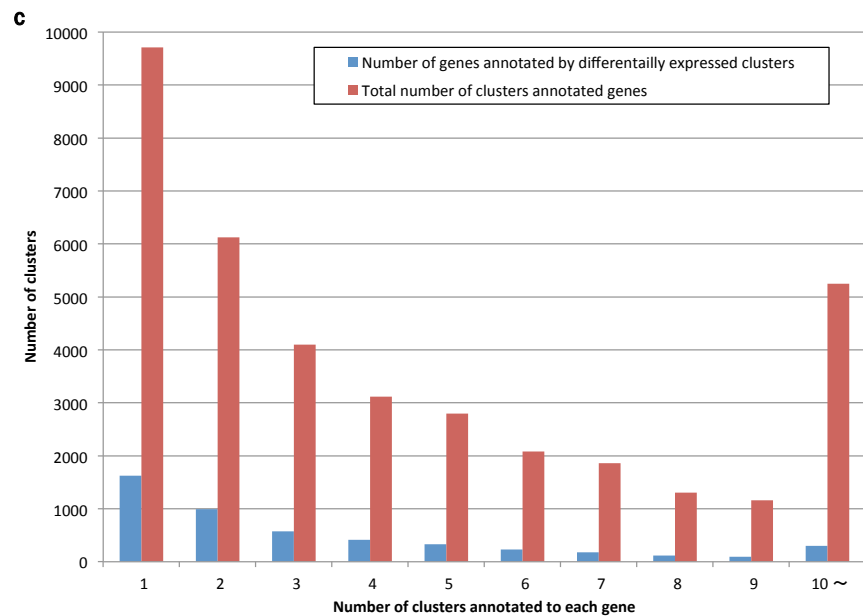

Supplement: Additional file 5 — GO terms describing differentially expressed genes. Gene ontology (GO) terms characterizing genes and the number of differentially expressed genes. The left most column shows GO terms (FDR < 0.05) that we interrogated for the differentially expressed genes identified by both the original Paraclu and our pipeline, and the next columns are the FDR and the number of genes characterized by the term, respectively. The other columns are the number of differentially expressed genes annotated to the terms and identified by RECLU or the original Paraclu. The low at the bottom of each table represents the sum of above values. [file 1471-2164-15-269-S5.PDF]
