# Supplementary material for: RECLU: a pipeline to discover reproducible transcriptional start sites and their alternative regulation using capped analysis of gene expression (CAGE)
Source: BMC Genomics. 2014 Apr 25;15:269. doi: 10.1186/1471-2164-15-269 (PMC4029093; doi:10.1186/1471-2164-15-269)
Supplement: Additional file 3 — Examples of the clusters with complicated structures. The distributions of clusters and TPMs for HeLa and THP-1 cells provided by Kanamori-Katayama et al. [11] are shown by the UCSC Genome Browser [36]. (A) Clusters annotated to TXNDC12 gene in chromosome 1 on the RefSeq hg18 genomes. The track at the top shows the location of these clusters. Below this, the next track indicates the five top peaks located around the transcription start sites of the gene. The one up-regulated peak of them is represented in green and the log fold change at the left of the cluster is more than 2.0, and the other top peaks in black are not significantly differentially expressed and the log fold change is 0.0. Likewise, the two bottom peaks are shown below. Since the peak at the left side is now significantly differentially expressed, the cluster is represented in black, while the other one is down-regulated and the cluster is orange and the log fold change is less than -2.0. “TPM for HeLa” and “TPM for THP-1” tracks show the mean TPM among replicates at each site for HeLa and THP-1 cells, respectively. The following tracks indicate genomic information for TXNDC12 based on the UCSC Genome Browser database. The two hierarchical cluster collections are annotated to TXNDC12, and the one of them is highly expressed for THP-1 cells than HeLa cells, while the other one has higher expression for HeLa cells. (B) Clusters annotated to JMJD5 gene is chromosome 16 on the RefSeq hg18 genome. For the details at each track, see the above description at (A). (C) Clusters annotated to C14orf1 gene in chromosome 14. There are two hierarchical cluster chunks and one of them is down-regulated and the other one is up-regulated. [file 1471-2164-15-269-S3.PDF]

1 CD14+CD16- monocytes vs. CD14+CD16+ monocytes (up-regulated)

GO term	FDR	Counts at both methods	Counts at RECLU	Counts at original Paraclu
response to wounding	0.00014	16	1	0
sum		16	1	0

2 CD14+CD16- monocytes vs. CD14+CD16+ monocytes (down-regulated)

GO term	FDR	Counts at both methods	Counts at RECLU	Counts at original Paraclu
sum		0	0	0

3 CD14+CD16- monocytes vs. CD14-CD16+ monocytes (up-regulated)

GO term	FDR	Counts at both methods	Counts at RECLU	Counts at original Paraclu
response to wounding	4.9e-11	40	1	0
inflammatory response	4e-08	28	6	0
defense response	1.5e-06	36	1	0
taxis	0.0093	14	0	0
chemotaxis	0.0093	14	0	0
positive regulation of response to stimulus	0.035	16	0	0
sum		148	8	0

4 CD14+CD16- monocytes vs. CD14-CD16+ monocytes (down-regulated)

GO term	FDR	Counts at both methods	Counts at RECLU	Counts at original Paraclu
protein-DNA complex assembly	0.0015	9	0	0
nucleosome assembly	0.011	8	6	1
chromatin assembly	0.013	8	0	0
nucleosome organization	0.021	8	0	0
sum		33	6	1

5 CD14+CD16- monocytes vs. CD14+ monocytes (up-regulated)

GO term	FDR	Counts at both methods	Counts at RECLU	Counts at original Paraclu
sum		0	0	0

6 CD14+CD16- monocytes vs. CD14+ monocytes (down-regulated)

GO term	FDR	Counts at both methods	Counts at RECLU	Counts at original Paraclu
cell activation	2.7e-12	38	0	0
leukocyte activation	2e-11	34	0	0
immune response	3.5e-11	58	12	0
positive regulation of immune system process	2.7e-09	31	0	0
lymphocyte activation	6.4e-09	28	0	0
regulation of apoptosis	2e-07	56	9	0
regulation of programmed cell death	2.9e-07	56	0	0
regulation of cell death	3.3e-07	56	0	0
response to wounding	1.7e-06	42	1	1
defense response	4.5e-06	45	2	0
regulation of cell proliferation	6.6e-06	52	6	0
positive regulation of cell communication	8.5e-06	31	0	0
positive regulation of signal transduction	1.2e-05	29	2	0
positive regulation of developmental process	1.3e-05	28	0	0
leukocyte differentiation	2.2e-05	19	0	0
regulation of T cell activation	2.4e-05	18	0	0
regulation of protein kinase cascade	2.5e-05	26	0	0
inflammatory response	2.5e-05	30	15	0
regulation of lymphocyte activation	2.8e-05	20	0	0
hemopoiesis	3.8e-05	25	4	0
positive regulation of cell proliferation	4e-05	34	18	1
immune system development	4.8e-05	27	0	0
T cell activation	7.6e-05	18	1	0
positive regulation of response to stimulus	0.00017	24	0	0
lymphocyte differentiation	0.00017	16	0	0
positive regulation of T cell activation	0.00018	14	2	0
regulation of leukocyte activation	0.00018	20	0	0
hemopoietic or lymphoid organ development	0.00024	25	0	0
positive regulation of leukocyte activation	0.00025	16	0	0
positive regulation of leukocyte proliferation	0.0004	12	0	0
positive regulation of mononuclear cell proliferation	0.0004	12	0	0
positive regulation of cell differentiation	0.00041	23	1	1
regulation of cell activation	0.00042	20	0	0
protein kinase cascade	0.00042	30	8	0
positive regulation of cell activation	0.00047	16	0	1
positive regulation of lymphocyte activation	0.00051	15	0	0
regulation of mononuclear cell proliferation	0.00059	14	0	0
regulation of leukocyte proliferation	0.00059	14	0	0
positive regulation of apoptosis	0.001	32	14	0
positive regulation of programmed cell death	0.0012	32	0	0
positive regulation of multicellular organismal process	0.0012	23	0	0
anti-apoptosis	0.0012	21	0	0
positive regulation of cell death	0.0013	32	1	1
intracellular signaling cascade	0.0013	64	0	0
positive regulation of cytokine production	0.0013	14	0	0
apoptosis	0.0015	39	32	1
immune response-activating signal transduction	0.0017	11	0	0
regulation of phosphorylation	0.0019	33	1	0
T cell differentiation	0.0019	12	3	0
programmed cell death	0.0021	39	0	0
positive regulation of lymphocyte proliferation	0.003	11	0	0
positive regulation of immune response	0.003	17	1	0
regulation of cytokine production	0.0032	19	1	0
regulation of lymphocyte proliferation	0.0036	13	0	0
immune response-regulating signal transduction	0.0036	11	0	0
positive regulation of locomotion	0.0036	14	0	0
cytokine-mediated signaling pathway	0.0041	12	10	0
positive regulation of protein kinase cascade	0.0044	18	0	0
regulation of phosphorus metabolic process	0.0045	33	0	0
regulation of phosphate metabolic process	0.0045	33	0	0
peptidyl-tyrosine phosphorylation	0.0053	10	3	1
immune effector process	0.0053	16	0	0
negative regulation of apoptosis	0.0061	27	30	0
cell death	0.007	42	16	0
peptidyl-tyrosine modification	0.0076	10	0	0
negative regulation of programmed cell death	0.0078	27	0	0
negative regulation of cell death	0.0082	27	0	0
death	0.0083	42	2	0
regulation of T cell proliferation	0.0093	11	1	0
enzyme linked receptor protein signaling pathway	0.01	26	0	0
regulation of lymphocyte differentiation	0.011	11	1	0
regulation of MAPKKK cascade	0.012	14	0	0
regulation of T cell differentiation	0.013	10	1	0
positive regulation of T cell proliferation	0.013	9	2	0
cell proliferation	0.032	29	19	0
positive regulation of myeloid cell differentiation	0.039	8	0	0
antigen receptor-mediated signaling pathway	0.039	8	1	0
protein amino acid phosphorylation	0.039	38	4	0
positive regulation of cell migration	0.044	12	3	0
sum		1982	227	7

7 CD14+CD16- monocytes vs. CD19+ B cells (up-regulated)

GO term	FDR	Counts at both methods	Counts at RECLU	Counts at original Paraclu
response to wounding	5.5e-11	70	2	0
inflammatory response	2.7e-07	46	23	0
defense response	1.2e-06	67	4	0
immune response	0.00027	66	21	1
actin cytoskeleton organization	0.0026	30	11	1
protein kinase cascade	0.0027	41	7	1
intracellular signaling cascade	0.0031	98	0	0
actin filament-based process	0.0096	30	0	0
positive regulation of transferase activity	0.025	29	0	0
regulation of cytokine production	0.037	24	0	0
sum		501	68	3

8 CD14+CD16- monocytes vs. CD19+ B cells (down-regulated)

GO term	FDR	Counts at both methods	Counts at RECLU	Counts at original Paraclu
nucleosome organization	2.5e-07	21	0	0
nucleosome assembly	2.9e-07	20	5	2
chromatin assembly	5.6e-07	20	0	0
protein-DNA complex assembly	1.3e-06	20	0	0
chromatin organization	3.8e-06	41	0	0
chromatin assembly or disassembly	1.4e-05	22	0	0
regulation of T cell activation	1.8e-05	21	0	0
regulation of lymphocyte activation	4.6e-05	23	0	0
regulation of alpha-beta T cell activation	7.5e-05	12	0	0
DNA packaging	9.8e-05	20	0	0
positive regulation of lymphocyte activation	0.00016	18	0	0
positive regulation of T cell activation	0.00017	16	3	0
chromosome organization	0.00018	44	0	0
regulation of cell activation	0.00023	24	0	0
regulation of leukocyte activation	0.00038	23	0	0
regulation of T cell differentiation	0.00044	13	0	0
positive regulation of leukocyte activation	0.00059	18	0	0
regulation of lymphocyte differentiation	0.00073	14	0	0
positive regulation of cell activation	0.0012	18	0	0
lymphocyte activation	0.0024	24	0	0
positive regulation of T cell differentiation	0.0029	10	2	0
positive regulation of alpha-beta T cell activation	0.0052	9	0	0
positive regulation of lymphocyte differentiation	0.0065	10	0	0
T cell activation	0.007	18	2	0
leukocyte activation	0.02	25	0	1
immune system development	0.022	27	0	0
hemopoietic or lymphoid organ development	0.023	26	0	0
hemopoiesis	0.041	24	6	0
regulation of alpha-beta T cell differentiation	0.044	8	0	0
positive regulation of immune system process	0.047	24	1	0
sum		613	19	3

9 CD14+CD16- monocytes vs. CD34 cells differentiated to erythrocyte lineage (up-regulated)

GO term	FDR	Counts at both methods	Counts at RECLU	Counts at original Paraclu
immune response	8.4e-17	122	7	1
leukocyte activation	7.7e-10	54	1	0
response to wounding	3.9e-09	87	2	0
defense response	4.8e-09	96	2	0
inflammatory response	1.6e-08	62	8	0
cell activation	2.4e-08	57	0	0
lymphocyte activation	5.6e-08	45	0	0
regulation of apoptosis	4.3e-07	110	2	0
intracellular signaling cascade	5.3e-07	153	0	0
regulation of programmed cell death	7.8e-07	110	0	0
regulation of cell death	9.7e-07	110	1	0
positive regulation of immune system process	7.1e-06	46	0	0
apoptosis	1.5e-05	85	25	2
programmed cell death	3.1e-05	85	0	0
regulation of cytokine production	6.7e-05	37	0	0
positive regulation of apoptosis	9e-05	65	3	0
positive regulation of programmed cell death	0.00012	65	0	0
actin cytoskeleton organization	0.00012	42	11	1
regulation of T cell differentiation	0.00013	18	0	0
positive regulation of lymphocyte differentiation	0.00013	15	0	0
positive regulation of cell death	0.00014	65	0	0
protein kinase cascade	0.00015	58	1	1
regulation of alpha-beta T cell activation	0.0002	15	0	0
immune effector process	0.00021	30	0	0
death	0.00028	93	1	0
positive regulation of T cell differentiation	0.00031	14	0	0
cell death	0.00039	92	8	0
antigen processing and presentation of peptide antigen	0.00046	13	0	0
T cell activation	0.00072	28	2	0
actin filament-based process	0.00072	42	0	0
regulation of lymphocyte differentiation	0.00073	19	0	0
antigen processing and presentation	0.00077	22	2	1
regulation of alpha-beta T cell differentiation	0.0011	12	0	0
regulation of I-kappaB kinase/NF-kappaB cascade	0.0013	25	0	0
innate immune response	0.0014	29	29	0
positive regulation of alpha-beta T cell activation	0.0017	12	0	0
lymphocyte proliferation	0.0018	15	0	0
regulation of lymphocyte activation	0.0019	30	0	0
regulation of T cell activation	0.002	26	0	0
positive regulation of cell activation	0.0026	25	0	0
regulation of actin cytoskeleton organization	0.0027	22	1	0
positive regulation of I-kappaB kinase/NF-kappaB cascade	0.0031	23	7	0
positive regulation of lymphocyte activation	0.0031	23	0	0
positive regulation of cytokine production	0.0032	22	0	0
leukocyte proliferation	0.0034	15	0	0
mononuclear cell proliferation	0.0034	15	0	0
regulation of cytoskeleton organization	0.0035	28	0	0
positive regulation of immune response	0.004	29	1	0
regulation of protein kinase cascade	0.0045	41	0	0
regulation of actin filament-based process	0.0047	22	0	0
myeloid leukocyte activation	0.0062	15	0	0
regulation of CD4-positive, alpha beta T cell differentiation	0.0064	9	0	0
regulation of cell activation	0.0079	32	1	0
positive regulation of protein kinase cascade	0.0083	31	0	0
positive regulation of alpha-beta T cell differentiation	0.0093	10	2	0
regulation of actin filament polymerization	0.0099	16	1	0
regulation of actin polymerization or depolymerization	0.011	17	0	0
positive regulation of T cell activation	0.014	19	0	0
positive regulation of leukocyte activation	0.015	23	0	0
regulation of actin filament length	0.017	17	0	0
leukocyte differentiation	0.017	26	0	0
hemopoiesis	0.019	38	2	0
positive regulation of response to stimulus	0.019	38	0	0
regulation of leukocyte activation	0.021	30	0	0
T cell proliferation	0.022	11	1	0
negative regulation of apoptosis	0.026	50	23	0
positive regulation of transferase activity	0.028	38	0	0
positive regulation of kinase activity	0.028	37	0	0
lymphocyte differentiation	0.031	22	0	0
regulation of cell proliferation	0.033	91	7	1
anti-apoptosis	0.035	34	0	0
regulation of small GTPase mediated signal transduction	0.036	39	4	0
positive regulation of cell communication	0.037	47	0	0
negative regulation of programmed cell death	0.038	50	0	0
positive regulation of multicellular organismal process	0.04	38	0	0
negative regulation of cell death	0.042	50	2	0
regulation of protein complex assembly	0.046	20	0	0
regulation of protein polymerization	0.049	17	0	0
sum		3234	157	7

10 CD14+CD16- monocytes vs. CD34 cells differentiated to erythrocyte lineage (down-regulated)

GO term	FDR	Counts at both methods	Counts at RECLU	Counts at original Paraclu
protein-DNA complex assembly	1.4e-20	38	0	0
nucleosome organization	3.4e-20	38	0	0
DNA packaging	4.3e-20	42	0	0
nucleosome assembly	8.5e-20	36	1	0
chromatin assembly	3.5e-19	36	0	0
cell cycle	2.3e-18	110	10	0
chromosome organization	3.7e-18	83	0	0
chromatin assembly or disassembly	9.2e-16	39	0	0
cell cycle phase	5.6e-14	69	0	0
cell cycle process	7.9e-13	81	1	0
M phase	1.1e-11	57	0	0
cellular macromolecular complex subunit organization	2.9e-11	59	0	0
chromatin organization	1.1e-10	60	0	0
cellular macromolecular complex assembly	1.4e-10	54	0	0
mitotic cell cycle	1.7e-09	57	7	0
DNA metabolic process	1.9e-09	69	0	0
organelle fission	9.7e-09	42	0	0
mitosis	4.4e-08	40	15	0
nuclear division	4.4e-08	40	0	0
cell division	7.1e-08	47	13	0
M phase of mitotic cell cycle	7.8e-08	40	0	0
macromolecular complex subunit organization	5.7e-06	77	0	0
macromolecular complex assembly	9.5e-06	73	0	0
chromosome segregation	9.9e-06	21	3	0
DNA replication	1e-05	33	4	0
response to DNA damage stimulus	2e-05	49	5	0
DNA repair	0.00072	38	9	0
cellular response to stress	0.002	59	0	0
microtubule-based process	0.003	34	2	0
regulation of cell cycle	0.0046	40	1	0
DNA-dependent DNA replication	0.015	14	1	0
DNA replication initiation	0.016	8	2	0
M phase of meiotic cell cycle	0.026	18	0	0
meiosis	0.026	18	1	1
cytoskeleton organization	0.027	46	1	1
meiotic cell cycle	0.034	18	1	0
negative regulation of organelle organization	0.043	16	0	0
nitrogen compound biosynthetic process	0.043	37	0	0
sum		1736	77	2

11 CD14+CD16- monocytes vs. CD4+CD25-CD45RA+ naive conventional T cell (up-regulated)

GO term	FDR	Counts at both methods	Counts at RECLU	Counts at original Paraclu
response to wounding	3.9e-15	87	2	0
defense response	2e-12	90	2	0
inflammatory response	2.4e-12	61	12	0
immune response	1.1e-10	93	8	1
protein kinase cascade	2.9e-07	56	1	0
leukocyte activation	3.7e-07	43	1	0
cell activation	2.5e-06	46	0	0
regulation of apoptosis	3.2e-06	91	3	0
regulation of programmed cell death	5.2e-06	91	0	0
regulation of cell death	6.2e-06	91	2	0
immune effector process	2.6e-05	28	0	0
lymphocyte activation	3e-05	35	0	0
regulation of cytokine production	3.4e-05	33	0	0
regulation of cell proliferation	4.9e-05	86	5	0
innate immune response	5e-05	28	32	0
apoptosis	5.2e-05	71	36	2
response to organic substance	8.6e-05	80	1	0
programmed cell death	9.3e-05	71	0	0
positive regulation of transferase activity	0.00012	38	2	0
regulation of MAPKKK cascade	0.00012	24	1	0
intracellular signaling cascade	0.00014	120	0	0
positive regulation of apoptosis	0.00018	55	9	0
positive regulation of programmed cell death	0.00022	55	0	0
actin cytoskeleton organization	0.00024	36	11	1
positive regulation of cell death	0.00026	55	1	0
positive regulation of immune system process	0.00029	37	0	0
positive regulation of kinase activity	0.00041	36	0	0
MAPKKK cascade	0.00061	31	1	0
actin filament-based process	0.0012	36	0	0
positive regulation of multicellular organismal process	0.0016	36	0	0
death	0.0018	76	0	0
positive regulation of immune response	0.002	26	1	0
positive regulation of JUN kinase activity	0.0021	12	3	0
regulation of transferase activity	0.0025	47	0	0
regulation of protein kinase cascade	0.0025	36	0	0
cell death	0.0027	75	12	0
positive regulation of protein kinase activity	0.0045	33	1	0
positive regulation of response to stimulus	0.0057	34	0	0
lymphocyte proliferation	0.0062	13	0	0
regulation of JNK cascade	0.0073	16	0	1
positive regulation of cytokine production	0.0074	19	0	0
cell proliferation	0.0078	51	16	0
regulation of cytokine biosynthetic process	0.0087	17	0	0
regulation of JUN kinase activity	0.01	12	0	0
leukocyte proliferation	0.011	13	0	0
mononuclear cell proliferation	0.011	13	0	0
regulation of kinase activity	0.011	44	0	0
positive regulation of developmental process	0.012	37	0	0
leukocyte mediated immunity	0.016	18	0	0
regulation of stress-activated protein kinase signaling pathway	0.016	16	0	0
regulation of phosphorus metabolic process	0.017	54	0	0
regulation of phosphate metabolic process	0.017	54	0	0
regulation of protein amino acid phosphorylation	0.017	27	0	0
positive regulation of cell communication	0.018	41	0	0
lymphocyte mediated immunity	0.019	16	0	0
anti-apoptosis	0.019	30	0	0
regulation of phosphorylation	0.024	52	0	0
regulation of cytoskeleton organization	0.025	23	1	0
sum		2645	164	5

12 CD14+CD16- monocytes vs. CD4+CD25-CD45RA+ naive conventional T cell (down-regulated)

GO term	FDR	Counts at both methods	Counts at RECLU	Counts at original Paraclu
protein-DNA complex assembly	4.9e-06	20	0	0
nucleosome organization	7.2e-06	20	0	0
nucleosome assembly	8.3e-06	19	4	2
chromatin assembly	1.5e-05	19	0	0
regulation of T cell activation	7e-05	21	0	0
chromatin assembly or disassembly	0.00029	21	0	0
DNA packaging	0.00035	20	0	0
lymphocyte activation	0.0028	25	0	0
regulation of lymphocyte activation	0.0035	21	0	0
lymphocyte differentiation	0.0059	17	0	0
positive regulation of immune system process	0.0066	27	0	0
leukocyte differentiation	0.0089	19	0	0
regulation of cell activation	0.013	22	0	0
hemopoiesis	0.017	26	5	0
chromatin organization	0.018	35	0	0
regulation of leukocyte activation	0.021	21	0	0
leukocyte activation	0.027	26	0	0
hemopoietic or lymphoid organ development	0.032	27	0	0
sum		406	9	2

13 CD14+CD16- monocytes vs. CD4+ T cells (up-regulated)

GO term	FDR	Counts at both methods	Counts at RECLU	Counts at original Paraclu
response to wounding	5.7e-12	79	2	0
immune response	6.4e-11	91	13	1
inflammatory response	2.8e-10	56	21	1
defense response	3e-10	83	6	0
leukocyte activation	1.3e-06	41	0	0
protein kinase cascade	4.6e-06	52	2	1
cell activation	6.8e-06	44	0	0
lymphocyte activation	3.8e-05	34	0	0
immune effector process	4.6e-05	27	0	0
regulation of cytokine production	0.00017	31	0	0
intracellular signaling cascade	0.00027	115	0	0
response to organic substance	0.00028	76	1	0
positive regulation of immune system process	0.00031	36	0	0
innate immune response	0.00034	26	40	0
positive regulation of transferase activity	0.00038	36	1	0
positive regulation of multicellular organismal process	0.00058	36	0	0
actin cytoskeleton organization	0.00083	34	9	1
positive regulation of immune response	0.00091	26	0	0
positive regulation of kinase activity	0.0014	34	0	0
regulation of apoptosis	0.0014	80	9	0
regulation of programmed cell death	0.002	80	0	0
regulation of cell death	0.0023	80	2	0
apoptosis	0.0024	64	35	2
positive regulation of cell communication	0.0026	42	0	0
MAPKKK cascade	0.0027	29	2	0
actin filament-based process	0.0036	34	0	0
programmed cell death	0.0039	64	0	0
positive regulation of cytokine production	0.0041	19	0	0
regulation of cell proliferation	0.0043	77	6	0
positive regulation of protein kinase activity	0.0053	32	1	0
positive regulation of response to stimulus	0.0064	33	0	0
regulation of tumor necrosis factor production	0.0084	11	0	0
death	0.01	71	0	0
T cell activation	0.013	22	2	0
cell death	0.016	70	8	0
regulation of MAPKKK cascade	0.018	20	1	0
regulation of cytokine biosynthetic process	0.024	16	0	0
regulation of transferase activity	0.024	43	0	0
cell proliferation	0.026	48	20	0
lymphocyte proliferation	0.027	12	1	0
positive regulation of developmental process	0.031	35	0	0
leukocyte mediated immunity	0.038	17	0	0
positive regulation of apoptosis	0.039	47	14	0
adaptive immune response based on somatic recombination of immune receptors built from immunoglobulin superfamily domains	0.039	16	0	0
adaptive immune response	0.039	16	2	0
regulation of cytoskeleton organization	0.043	22	0	0
leukocyte proliferation	0.043	12	0	0
mononuclear cell proliferation	0.043	12	0	0
positive regulation of signal transduction	0.045	36	0	0
positive regulation of programmed cell death	0.046	47	0	0
sum		2164	198	6

14 CD14+CD16- monocytes vs. CD4+ T cells (down-regulated)

GO term	FDR	Counts at both methods	Counts at RECLU	Counts at original Paraclu
protein-DNA complex assembly	3.5e-10	28	0	0
nucleosome assembly	2.6e-09	26	4	1
chromatin assembly	6.1e-09	26	0	0
nucleosome organization	3.1e-08	26	0	0
chromatin assembly or disassembly	3.2e-07	29	0	0
DNA packaging	1.2e-06	27	0	0
chromatin organization	1e-05	51	1	0
chromosome organization	0.00039	56	2	0
regulation of T cell activation	0.00058	23	0	0
lymphocyte activation	0.00075	31	0	0
cellular macromolecular complex assembly	0.0014	41	0	0
regulation of lymphocyte activation	0.0029	25	0	0
cellular macromolecular complex subunit organization	0.0043	43	0	0
protein amino acid phosphorylation	0.0071	66	4	0
leukocyte activation	0.016	32	0	0
regulation of cell activation	0.018	26	0	0
phosphorus metabolic process	0.019	86	0	0
phosphate metabolic process	0.019	86	1	0
lymphocyte differentiation	0.02	19	0	0
regulation of leukocyte activation	0.022	25	0	0
hemopoiesis	0.026	31	7	0
hemopoietic or lymphoid organ development	0.027	33	0	0
T cell activation	0.029	21	3	0
sum		857	22	1

15 CD14+CD16- monocytes vs. CD8+ T cells (up-regulated)

GO term	FDR	Counts at both methods	Counts at RECLU	Counts at original Paraclu
response to wounding	1.4e-12	84	1	0
inflammatory response	5.4e-10	58	14	0
defense response	7e-09	84	5	0
immune response	8.2e-08	88	13	1
protein kinase cascade	3e-06	55	5	0
immune effector process	0.00021	27	0	0
regulation of apoptosis	0.0003	87	8	0
regulation of programmed cell death	0.00047	87	0	0
regulation of cell death	0.00055	87	2	0
positive regulation of transferase activity	0.00083	37	2	0
apoptosis	0.00091	69	33	2
intracellular signaling cascade	0.00092	120	0	0
regulation of MAPKKK cascade	0.00098	23	1	0
leukocyte activation	0.001	37	1	0
positive regulation of cell communication	0.0012	45	0	0
innate immune response	0.0014	26	32	0
programmed cell death	0.0016	69	0	0
actin cytoskeleton organization	0.0016	35	11	1
regulation of protein kinase cascade	0.002	37	0	0
positive regulation of kinase activity	0.0027	35	0	0
regulation of cytokine production	0.0028	30	0	0
cell activation	0.0036	40	0	0
regulation of cytoskeleton organization	0.0039	25	1	0
cell death	0.005	76	11	0
positive regulation of immune system process	0.0053	35	0	0
response to organic substance	0.0056	76	0	0
death	0.0066	76	2	0
actin filament-based process	0.007	35	0	0
positive regulation of protein kinase activity	0.0094	33	3	1
MAPKKK cascade	0.012	29	2	0
regulation of cell proliferation	0.012	80	5	0
regulation of cytokine biosynthetic process	0.014	17	0	0
positive regulation of apoptosis	0.014	51	12	0
regulation of organelle organization	0.014	32	0	0
positive regulation of programmed cell death	0.017	51	0	0
positive regulation of signal transduction	0.017	39	0	0
lymphocyte activation	0.019	30	0	0
positive regulation of cell death	0.019	51	0	0
positive regulation of JUN kinase activity	0.023	11	3	0
regulation of stress-activated protein kinase signaling pathway	0.024	16	0	0
regulation of transferase activity	0.032	45	0	0
anti-apoptosis	0.037	30	0	0
positive regulation of immune response	0.039	24	1	0
regulation of actin cytoskeleton organization	0.04	18	1	0
sum		2140	169	5

16 CD14+CD16- monocytes vs. CD8+ T cells (down-regulated)

GO term	FDR	Counts at both methods	Counts at RECLU	Counts at original Paraclu
protein-DNA complex assembly	5.7e-07	25	0	0
nucleosome assembly	3.6e-06	23	10	1
nucleosome organization	5.2e-06	24	0	0
chromatin assembly	7.3e-06	23	0	0
chromatin assembly or disassembly	0.00015	26	0	0
leukocyte activation	0.00016	38	0	0
lymphocyte activation	0.00037	33	0	0
DNA packaging	0.00051	24	1	0
chromatin organization	0.00073	49	1	0
lymphocyte differentiation	0.0037	21	0	0
leukocyte differentiation	0.0041	24	0	0
cell activation	0.0043	39	1	0
protein amino acid phosphorylation	0.0059	70	5	1
hemopoietic or lymphoid organ development	0.0074	36	0	0
regulation of T cell activation	0.0077	22	0	0
hemopoiesis	0.016	33	4	1
T cell activation	0.026	22	4	0
immune system development	0.028	36	0	0
phosphate metabolic process	0.035	90	1	0
phosphorus metabolic process	0.035	90	0	0
immune response	0.035	69	9	0
chromosome organization	0.037	53	3	0
sum		870	39	3

17 CD14+CD16- monocytes vs. Macrophage - monocyte derived (up-regulated)

GO term	FDR	Counts at both methods	Counts at RECLU	Counts at original Paraclu
death	5e-05	54	1	0
cell death	0.0001	53	7	0
apoptosis	0.00034	46	27	1
response to wounding	0.00049	42	1	0
programmed cell death	0.00052	46	0	0
immune response	0.0011	49	13	0
positive regulation of transferase activity	0.0021	25	0	0
positive regulation of kinase activity	0.0037	24	2	0
defense response	0.0038	44	7	0
positive regulation of protein kinase activity	0.0072	23	1	0
leukocyte activation	0.0082	24	2	0
lymphocyte activation	0.015	21	0	0
regulation of apoptosis	0.016	51	2	0
inflammatory response	0.016	28	14	0
regulation of programmed cell death	0.02	51	0	0
regulation of cell death	0.023	51	0	0
regulation of transferase activity	0.025	30	0	0
T cell activation	0.027	16	1	0
regulation of kinase activity	0.032	29	0	0
cell activation	0.045	25	0	0
regulation of protein kinase activity	0.046	28	1	0
regulation of phosphorylation	0.047	34	1	0
sum		794	80	1

18 CD14+CD16- monocytes vs. Macrophage - monocyte derived (down-regulated)

GO term	FDR	Counts at both methods	Counts at RECLU	Counts at original Paraclu
M phase	4.1e-06	45	0	0
cell cycle	1.9e-05	77	13	0
mitosis	2.6e-05	34	14	3
nuclear division	2.6e-05	34	0	0
M phase of mitotic cell cycle	4e-05	34	0	0
organelle fission	7e-05	34	0	0
cell cycle phase	8.1e-05	49	0	0
DNA packaging	0.00012	23	0	0
protein-DNA complex assembly	0.00016	20	0	0
cell cycle process	0.00057	58	0	0
nucleosome organization	0.0012	19	0	0
nucleosome assembly	0.0012	18	3	0
chromatin assembly	0.0021	18	1	0
cell division	0.0033	36	15	1
mitotic cell cycle	0.0065	41	9	1
chromosome segregation	0.018	16	4	3
sum		556	59	8

19 CD14+CD16- monocytes vs. Neutrophils (up-regulated)

GO term	FDR	Counts at both methods	Counts at RECLU	Counts at original Paraclu
translation	4.7e-31	111	8	0
ncRNA metabolic process	3.5e-19	75	5	0
translational elongation	1.2e-14	43	4	0
ribosome biogenesis	3.3e-14	47	2	0
ribonucleoprotein complex biogenesis	2e-13	57	0	0
ncRNA processing	6.1e-13	58	0	0
RNA processing	3.8e-10	107	6	1
rRNA metabolic process	8.6e-10	36	0	0
rRNA processing	6.7e-09	34	10	0
tRNA metabolic process	6.7e-07	36	0	0
tRNA aminoacylation	0.00071	18	0	0
amino acid activation	0.00071	18	0	0
tRNA aminoacylation for protein translation	0.00071	18	5	0
cellular respiration	0.0011	27	0	0
oxidation reduction	0.0067	96	1	0
ribosomal large subunit biogenesis	0.011	8	1	0
cofactor metabolic process	0.019	39	0	0
intracellular protein transport	0.024	62	13	0
cellular macromolecular complex subunit organization	0.043	59	0	0
cellular macromolecular complex assembly	0.047	54	0	0
mitochondrion organization	0.049	30	4	0
sum		1033	59	1

20 CD14+CD16- monocytes vs. Neutrophils (down-regulated)

GO term	FDR	Counts at both methods	Counts at RECLU	Counts at original Paraclu
immune response	4.3e-08	59	6	4
defense response	1.3e-07	54	2	0
inflammatory response	1.2e-05	34	7	2
intracellular signaling cascade	0.00013	77	0	0
response to wounding	0.00016	43	0	0
leukocyte activation	0.00062	26	0	1
cell activation	0.0045	27	0	0
protein amino acid phosphorylation	0.0049	46	2	2
protein kinase cascade	0.0075	31	1	0
myeloid leukocyte activation	0.025	10	0	0
sum		407	18	9

21 CD14+CD16+ monocytes vs. CD14-CD16+ monocytes (up-regulated)

GO term	FDR	Counts at both methods	Counts at RECLU	Counts at original Paraclu
response to wounding	6.5e-11	25	0	0
inflammatory response	6e-08	18	4	0
defense response	2.7e-05	20	2	0
immune response	0.018	17	4	1
sum		80	10	1

22 CD14+CD16+ monocytes vs. CD14-CD16+ monocytes (down-regulated)

GO term	FDR	Counts at both methods	Counts at RECLU	Counts at original Paraclu
sum		0	0	0

23 CD14+CD16+ monocytes vs. CD14+ monocytes (up-regulated)

GO term	FDR	Counts at both methods	Counts at RECLU	Counts at original Paraclu
translation	1.1e-06	39	8	1
mitochondrial electron transport, NADH to ubiquinone	0.00046	12	1	0
cellular respiration	0.00094	17	0	0
electron transport chain	0.0085	17	3	0
mitochondrial ATP synthesis coupled electron transport	0.0099	12	0	0
ATP synthesis coupled electron transport	0.0099	12	0	0
immune response	0.019	50	7	0
oxidative phosphorylation	0.027	15	3	0
respiratory electron transport chain	0.038	12	9	0
translational elongation	0.038	15	8	1
sum		201	39	2

24 CD14+CD16+ monocytes vs. CD14+ monocytes (down-regulated)

GO term	FDR	Counts at both methods	Counts at RECLU	Counts at original Paraclu
immune response	5.9e-09	68	10	0
regulation of apoptosis	1.1e-07	72	6	0
response to wounding	1.5e-07	55	0	1
regulation of programmed cell death	1.7e-07	72	0	0
regulation of cell death	2e-07	72	0	0
leukocyte activation	2.4e-06	33	0	0
inflammatory response	2.5e-06	39	12	0
cell activation	3.7e-06	36	0	0
hemopoiesis	5.2e-06	32	3	0
intracellular signaling cascade	6.4e-06	92	0	0
defense response	1.2e-05	56	2	0
hemopoietic or lymphoid organ development	1.5e-05	33	0	0
positive regulation of immune system process	2.5e-05	31	0	0
negative regulation of apoptosis	2.7e-05	39	20	1
negative regulation of programmed cell death	3.9e-05	39	0	0
negative regulation of cell death	4.2e-05	39	0	0
anti-apoptosis	5.8e-05	28	0	0
immune system development	6.2e-05	33	0	0
protein kinase cascade	8.7e-05	39	8	0
regulation of phosphorylation	9.1e-05	45	1	0
regulation of phosphate metabolic process	0.00029	45	0	0
regulation of phosphorus metabolic process	0.00029	45	1	0
leukocyte differentiation	0.00029	21	0	0
lymphocyte activation	0.00043	26	0	0
regulation of cell proliferation	0.0017	60	5	0
positive regulation of locomotion	0.002	17	0	0
protein amino acid phosphorylation	0.0024	53	4	0
lymphocyte differentiation	0.004	17	0	0
taxis	0.0075	21	0	0
chemotaxis	0.0075	21	6	0
positive regulation of developmental process	0.0076	29	0	0
positive regulation of cell motion	0.01	16	2	0
positive regulation of cell migration	0.015	15	2	0
regulation of transferase activity	0.017	34	0	0
apoptosis	0.018	47	27	2
cell migration	0.019	28	6	1
cell death	0.022	53	6	0
regulation of T cell activation	0.022	17	0	0
positive regulation of apoptosis	0.024	37	11	0
positive regulation of cell communication	0.026	31	0	0
death	0.026	53	1	0
programmed cell death	0.026	47	0	0
positive regulation of cell proliferation	0.026	36	16	1
positive regulation of programmed cell death	0.028	37	0	0
positive regulation of cell death	0.031	37	1	1
immune effector process	0.032	18	0	0
regulation of lymphocyte activation	0.033	19	0	0
regulation of mononuclear cell proliferation	0.037	14	0	0
regulation of leukocyte proliferation	0.037	14	0	0
negative regulation of macromolecule metabolic process	0.038	53	0	0
cell motion	0.039	39	8	0
regulation of kinase activity	0.048	32	0	0
sum		1985	158	7

25 CD14+CD16+ monocytes vs. CD19+ B cells (up-regulated)

GO term	FDR	Counts at both methods	Counts at RECLU	Counts at original Paraclu
response to wounding	3.2e-09	59	2	0
defense response	6.1e-09	64	1	0
inflammatory response	2.3e-08	43	19	1
immune response	5.5e-06	62	16	0
innate immune response	0.00093	21	43	1
immune effector process	0.0025	20	0	0
positive regulation of immune response	0.0084	20	1	0
intracellular signaling cascade	0.014	82	0	0
positive regulation of immune system process	0.016	26	0	0
positive regulation of defense response	0.049	13	0	0
sum		410	82	2

26 CD14+CD16+ monocytes vs. CD19+ B cells (down-regulated)

GO term	FDR	Counts at both methods	Counts at RECLU	Counts at original Paraclu
chromatin organization	7.2e-06	41	0	0
regulation of T cell activation	0.00014	20	0	0
nucleosome organization	0.00065	17	0	0
chromosome organization	0.00087	43	0	0
nucleosome assembly	0.00091	16	7	0
lymphocyte activation	0.00094	25	0	0
regulation of alpha-beta T cell activation	0.001	11	0	0
regulation of lymphocyte activation	0.0014	21	0	0
chromatin assembly	0.0015	16	0	0
positive regulation of T cell activation	0.0015	15	3	0
T cell activation	0.0021	19	1	0
chromatin assembly or disassembly	0.0024	19	0	0
protein-DNA complex assembly	0.0026	16	0	0
regulation of T cell differentiation	0.0044	12	0	0
regulation of cell activation	0.0052	22	0	0
positive regulation of lymphocyte activation	0.0061	16	0	0
regulation of lymphocyte differentiation	0.0062	13	0	0
regulation of leukocyte activation	0.0084	21	0	0
leukocyte activation	0.0095	26	0	0
positive regulation of leukocyte activation	0.019	16	0	0
regulation of transcription	0.028	138	0	0
positive regulation of cell activation	0.033	16	0	0
positive regulation of T cell differentiation	0.033	9	1	0
sum		568	12	0

27 CD14+CD16+ monocytes vs. CD34 cells differentiated to erythrocyte lineage (up-regulated)

GO term	FDR	Counts at both methods	Counts at RECLU	Counts at original Paraclu
immune response	2.4e-20	119	8	0
defense response	4.8e-10	90	1	0
inflammatory response	5e-10	60	7	2
response to wounding	2.6e-09	80	1	0
leukocyte activation	1.2e-08	48	1	0
cell activation	4.9e-08	52	0	0
regulation of apoptosis	7.1e-08	102	4	0
regulation of programmed cell death	1.3e-07	102	0	0
regulation of cell death	1.6e-07	102	0	0
lymphocyte activation	5.7e-07	40	0	0
positive regulation of apoptosis	2.8e-06	63	8	1
positive regulation of programmed cell death	3.7e-06	63	0	0
positive regulation of cell death	4.5e-06	63	0	0
intracellular signaling cascade	6.1e-06	134	0	0
apoptosis	8.1e-06	78	27	0
T cell activation	1e-05	29	2	1
positive regulation of immune system process	1.1e-05	42	0	0
programmed cell death	1.5e-05	78	0	0
actin cytoskeleton organization	2.5e-05	40	5	0
positive regulation of T cell differentiation	6.1e-05	14	0	0
regulation of T cell differentiation	0.00012	17	0	0
death	0.00014	85	1	0
actin filament-based process	0.00015	40	0	0
immune effector process	0.00017	28	0	0
positive regulation of lymphocyte differentiation	0.00022	14	0	0
cell death	0.00022	84	6	0
positive regulation of immune response	0.00025	29	0	0
regulation of alpha-beta T cell activation	0.00032	14	0	0
innate immune response	0.00032	28	23	2
antigen processing and presentation	0.00038	21	1	0
positive regulation of alpha-beta T cell activation	0.00043	12	0	0
antigen processing and presentation of peptide antigen	0.0011	12	0	0
regulation of actin cytoskeleton organization	0.0013	21	2	0
leukocyte differentiation	0.0015	26	0	0
regulation of actin filament polymerization	0.0018	16	0	0
regulation of actin polymerization or depolymerization	0.0018	17	0	0
regulation of actin filament-based process	0.0022	21	0	0
regulation of cytokine production	0.0028	31	0	0
regulation of alpha-beta T cell differentiation	0.0029	11	0	0
regulation of actin filament length	0.0029	17	0	0
regulation of lymphocyte differentiation	0.0029	17	1	0
positive regulation of alpha-beta T cell differentiation	0.0029	10	0	0
regulation of cytoskeleton organization	0.0031	26	0	0
lymphocyte differentiation	0.0037	22	0	0
hemopoiesis	0.0057	36	3	0
positive regulation of cytokine production	0.0065	20	0	0
T cell proliferation	0.0066	11	1	0
regulation of I-kappaB kinase/NF-kappaB cascade	0.007	22	1	0
myeloid leukocyte activation	0.0076	14	0	0
regulation of T cell activation	0.0086	23	0	0
positive regulation of T cell activation	0.0092	18	2	0
protein kinase cascade	0.011	48	4	0
positive regulation of cell activation	0.013	22	0	0
regulation of lymphocyte activation	0.015	26	0	0
positive regulation of response to stimulus	0.015	35	0	0
positive regulation of protein kinase cascade	0.015	28	0	0
lymphocyte proliferation	0.016	13	0	0
regulation of organelle organization	0.017	33	0	0
regulation of protein kinase cascade	0.019	36	0	0
hemopoietic or lymphoid organ development	0.02	37	0	0
positive regulation of lymphocyte activation	0.021	20	0	0
positive regulation of I-kappaB kinase/NF-kappaB cascade	0.021	20	11	1
T cell differentiation	0.022	16	2	0
regulation of protein complex assembly	0.026	19	0	0
leukocyte proliferation	0.027	13	0	0
mononuclear cell proliferation	0.027	13	0	0
immune system development	0.032	38	0	0
regulation of cell activation	0.036	28	1	0
regulation of protein polymerization	0.039	16	0	0
sum		2623	123	7

28 CD14+CD16+ monocytes vs. CD34 cells differentiated to erythrocyte lineage (down-regulated)

GO term	FDR	Counts at both methods	Counts at RECLU	Counts at original Paraclu
protein-DNA complex assembly	1.4e-18	36	0	0
DNA packaging	2.8e-18	40	0	0
nucleosome organization	3.3e-18	36	0	0
nucleosome assembly	9.7e-18	34	3	0
chromosome organization	2.9e-17	81	1	0
chromatin assembly	3.6e-17	34	0	0
cell cycle	1e-16	106	10	0
chromatin assembly or disassembly	5e-15	38	0	0
cell cycle phase	2e-13	68	0	0
M phase	6.6e-12	57	0	0
cell cycle process	1.4e-11	78	1	0
organelle fission	3.7e-10	44	0	0
chromatin organization	8e-10	58	0	0
nuclear division	1.7e-09	42	0	0
mitosis	1.7e-09	42	8	0
M phase of mitotic cell cycle	3.2e-09	42	0	0
mitotic cell cycle	3.8e-09	56	7	0
cell division	1.4e-08	48	10	0
cellular macromolecular complex subunit organization	1.1e-07	52	0	0
DNA metabolic process	2.3e-07	64	0	0
cellular macromolecular complex assembly	6.6e-07	47	0	0
chromosome segregation	8.2e-06	21	0	0
DNA replication	3e-05	32	7	0
regulation of cell cycle	0.00051	42	2	0
macromolecular complex subunit organization	0.00089	70	0	0
DNA replication initiation	0.00089	9	2	0
macromolecular complex assembly	0.0016	66	0	0
microtubule-based process	0.0023	34	2	0
response to DNA damage stimulus	0.0046	43	3	0
cytoskeleton organization	0.0092	47	1	1
DNA-dependent DNA replication	0.013	14	0	0
DNA repair	0.029	34	11	0
DNA duplex unwinding	0.037	8	1	0
DNA geometric change	0.037	8	0	0
negative regulation of organelle organization	0.037	16	0	0
sum		1547	69	1

29 CD14+CD16+ monocytes vs. CD4+CD25-CD45RA+ naive conventional T cell (up-regulated)

GO term	FDR	Counts at both methods	Counts at RECLU	Counts at original Paraclu
immune response	1.8e-12	91	11	0
response to wounding	2.2e-12	77	1	0
inflammatory response	5.8e-12	57	10	1
defense response	9.3e-11	81	1	0
immune effector process	1.2e-07	30	0	0
regulation of apoptosis	2.4e-06	85	8	0
intracellular signaling cascade	2.5e-06	117	0	0
regulation of programmed cell death	3.9e-06	85	0	0
regulation of cell death	4.6e-06	85	2	0
positive regulation of apoptosis	5.5e-06	55	8	0
positive regulation of immune system process	6.4e-06	38	0	0
positive regulation of programmed cell death	7e-06	55	0	0
positive regulation of cell death	8.2e-06	55	0	0
actin cytoskeleton organization	1.9e-05	36	10	0
positive regulation of immune response	7.4e-05	27	0	0
actin filament-based process	0.0001	36	0	0
innate immune response	0.00011	26	35	1
cell activation	0.00011	40	0	0
leukocyte activation	0.00011	36	1	0
apoptosis	0.00014	65	31	1
leukocyte mediated immunity	0.00016	20	0	0
lymphocyte mediated immunity	0.00018	18	0	0
programmed cell death	0.00023	65	0	0
regulation of cell proliferation	0.00042	77	6	0
positive regulation of response to stimulus	0.00059	34	0	0
lymphocyte activation	0.0013	30	0	0
regulation of cytoskeleton organization	0.0013	24	0	0
regulation of actin cytoskeleton organization	0.0014	19	2	0
regulation of cytokine production	0.0019	28	0	0
protein kinase cascade	0.002	44	4	0
regulation of actin filament-based process	0.0024	19	0	0
regulation of organelle organization	0.0026	31	0	0
cell death	0.0041	69	10	0
death	0.0051	69	0	0
response to organic substance	0.0089	68	2	0
monosaccharide metabolic process	0.012	30	0	0
adaptive immune response based on somatic recombination of immune receptors built from immunoglobulin superfamily domains	0.019	16	0	0
adaptive immune response	0.019	16	3	0
positive regulation of transferase activity	0.02	31	0	0
positive regulation of kinase activity	0.026	30	0	0
regulation of kinase activity	0.027	40	0	0
regulation of MAPKKK cascade	0.029	19	1	0
regulation of transferase activity	0.031	41	0	0
regulation of phosphorylation	0.031	48	0	0
positive regulation of cytokine production	0.033	17	0	0
immunoglobulin mediated immune response	0.037	13	2	0
regulation of protein kinase cascade	0.042	31	0	0
regulation of phosphate metabolic process	0.042	49	0	0
regulation of phosphorus metabolic process	0.042	49	0	0
positive regulation of defense response	0.045	15	0	0
sum		2237	148	3

30 CD14+CD16+ monocytes vs. CD4+CD25-CD45RA+ naive conventional T cell (down-regulated)

GO term	FDR	Counts at both methods	Counts at RECLU	Counts at original Paraclu
protein-DNA complex assembly	0.00067	17	0	0
nucleosome organization	0.00091	17	0	0
nucleosome assembly	0.0013	16	8	0
lymphocyte activation	0.0015	25	0	0
chromatin assembly	0.002	16	0	0
chromatin assembly or disassembly	0.0035	19	0	0
lymphocyte differentiation	0.0038	17	0	0
regulation of T cell activation	0.0047	18	0	0
leukocyte differentiation	0.0054	19	0	0
hemopoiesis	0.0095	26	3	0
positive regulation of immune system process	0.011	26	0	0
leukocyte activation	0.015	26	0	0
hemopoietic or lymphoid organ development	0.018	27	0	0
DNA packaging	0.02	17	0	0
protein amino acid phosphorylation	0.023	50	2	0
sum		336	13	0

31 CD14+CD16+ monocytes vs. CD4+ T cells (up-regulated)

GO term	FDR	Counts at both methods	Counts at RECLU	Counts at original Paraclu
response to wounding	1.2e-12	77	2	0
immune response	2.2e-12	90	10	0
inflammatory response	3.2e-12	57	11	0
defense response	1.3e-10	80	1	0
immune effector process	2.1e-06	28	0	0
positive regulation of immune system process	4.4e-06	38	0	0
intracellular signaling cascade	4.4e-06	115	0	0
positive regulation of immune response	1.3e-05	28	0	0
leukocyte activation	2.4e-05	37	0	0
cell activation	2.5e-05	41	0	0
positive regulation of response to stimulus	0.00013	35	0	0
lymphocyte activation	0.00029	31	0	0
regulation of apoptosis	0.00058	77	14	0
regulation of programmed cell death	0.00083	77	0	0
regulation of cell death	0.00098	77	2	0
innate immune response	0.0013	24	38	1
protein kinase cascade	0.0014	44	9	0
leukocyte mediated immunity	0.0034	18	0	0
lymphocyte mediated immunity	0.0047	16	0	0
response to organic substance	0.0055	68	2	0
regulation of actin cytoskeleton organization	0.0055	18	2	0
positive regulation of apoptosis	0.0057	47	15	0
positive regulation of programmed cell death	0.0069	47	1	0
positive regulation of cell death	0.0079	47	1	0
apoptosis	0.0086	59	38	0
regulation of actin filament-based process	0.0089	18	0	0
actin cytoskeleton organization	0.013	30	11	1
programmed cell death	0.014	59	0	0
regulation of cytoskeleton organization	0.014	22	0	0
T cell activation	0.015	21	3	0
regulation of cytokine production	0.015	26	0	0
adaptive immune response	0.016	16	3	0
adaptive immune response based on somatic recombination of immune receptors built from immunoglobulin superfamily domains	0.016	16	0	0
regulation of MAPKKK cascade	0.024	19	1	0
positive regulation of cytokine production	0.028	17	0	0
immunoglobulin mediated immune response	0.032	13	2	0
positive regulation of defense response	0.039	15	0	0
positive regulation of transferase activity	0.042	30	1	0
actin filament-based process	0.045	30	0	0
B cell mediated immunity	0.047	13	0	0
regulation of organelle organization	0.047	28	0	0
sum		1649	167	2

32 CD14+CD16+ monocytes vs. CD4+ T cells (down-regulated)

GO term	FDR	Counts at both methods	Counts at RECLU	Counts at original Paraclu
protein-DNA complex assembly	8.5e-05	21	0	0
chromatin organization	8.7e-05	48	1	0
nucleosome assembly	0.00011	20	11	0
chromatin assembly	0.00021	20	0	0
chromatin assembly or disassembly	0.00038	24	0	0
nucleosome organization	0.00063	20	0	0
lymphocyte activation	0.0042	29	0	0
chromosome organization	0.0044	52	2	0
DNA packaging	0.006	21	1	0
protein amino acid phosphorylation	0.022	63	2	0
regulation of T cell activation	0.023	20	0	0
sum		338	17	0

33 CD14+CD16+ monocytes vs. CD8+ T cells (up-regulated)

GO term	FDR	Counts at both methods	Counts at RECLU	Counts at original Paraclu
inflammatory response	4e-12	59	8	0
response to wounding	1.3e-11	78	1	0
immune response	1.3e-10	90	11	0
defense response	6.2e-10	82	0	0
positive regulation of immune system process	8.1e-06	39	0	0
intracellular signaling cascade	1.7e-05	119	0	0
immune effector process	4.1e-05	27	0	0
regulation of actin cytoskeleton organization	0.00014	21	2	0
positive regulation of immune response	0.00022	27	0	0
regulation of actin filament-based process	0.00024	21	0	0
actin cytoskeleton organization	0.00073	34	8	0
regulation of cytoskeleton organization	0.0009	25	0	0
regulation of apoptosis	0.0011	80	11	0
innate immune response	0.0012	25	36	0
regulation of programmed cell death	0.0016	80	0	0
regulation of actin polymerization or depolymerization	0.0017	16	0	0
regulation of cell death	0.0019	80	1	0
regulation of actin filament polymerization	0.002	15	1	0
positive regulation of response to stimulus	0.002	34	0	0
regulation of actin filament length	0.0026	16	0	0
positive regulation of apoptosis	0.0031	50	17	0
actin filament-based process	0.0032	34	0	0
protein kinase cascade	0.0034	45	7	0
cell activation	0.0034	38	0	0
regulation of cell proliferation	0.0035	77	6	1
leukocyte activation	0.0035	34	0	0
positive regulation of programmed cell death	0.0038	50	0	0
positive regulation of cell death	0.0043	50	0	0
regulation of organelle organization	0.0077	31	0	0
cell death	0.014	70	5	0
response to organic substance	0.015	70	2	0
regulation of protein complex assembly	0.016	18	0	0
death	0.017	70	0	0
apoptosis	0.017	61	34	0
negative regulation of protein complex assembly	0.02	11	3	0
positive regulation of transferase activity	0.022	32	1	0
positive regulation of kinase activity	0.027	31	0	0
programmed cell death	0.027	61	0	0
lymphocyte activation	0.033	28	0	0
regulation of protein polymerization	0.036	15	0	0
Ras protein signal transduction	0.036	19	3	0
leukocyte mediated immunity	0.036	17	0	0
adaptive immune response	0.037	16	3	0
adaptive immune response based on somatic recombination of immune receptors built from immunoglobulin superfamily domains	0.037	16	0	0
regulation of cytokine production	0.049	26	0	0
sum		1938	160	1

34 CD14+CD16+ monocytes vs. CD8+ T cells (down-regulated)

GO term	FDR	Counts at both methods	Counts at RECLU	Counts at original Paraclu
protein-DNA complex assembly	0.00029	21	0	0
protein amino acid phosphorylation	0.0011	71	6	1
nucleosome assembly	0.0018	19	11	0
nucleosome organization	0.002	20	0	0
lymphocyte activation	0.002	31	0	0
leukocyte activation	0.0021	35	0	0
chromatin assembly	0.0031	19	0	0
hemopoietic or lymphoid organ development	0.004	36	0	0
chromatin organization	0.0047	46	1	0
leukocyte differentiation	0.0091	23	0	0
hemopoiesis	0.0092	33	3	0
lymphocyte differentiation	0.0097	20	0	0
cell activation	0.015	37	0	0
immune system development	0.015	36	0	0
chromatin assembly or disassembly	0.019	22	0	0
phosphorus metabolic process	0.035	88	0	0
phosphate metabolic process	0.035	88	2	0
sum		645	23	1

35 CD14+CD16+ monocytes vs. Macrophage - monocyte derived (up-regulated)

GO term	FDR	Counts at both methods	Counts at RECLU	Counts at original Paraclu
translational elongation	1.2e-07	20	5	1
lymphocyte activation	2.7e-05	24	0	0
leukocyte activation	6.1e-05	26	1	0
cell activation	0.00012	28	0	0
immune response	0.00017	47	7	0
T cell activation	0.00022	18	1	0
regulation of apoptosis	0.00039	51	4	0
apoptosis	0.00051	42	25	0
regulation of programmed cell death	0.00053	51	0	0
regulation of cell death	0.0006	51	0	0
death	0.00068	47	1	0
programmed cell death	0.00077	42	0	0
cell death	0.0014	46	7	0
positive regulation of apoptosis	0.0017	33	10	0
positive regulation of programmed cell death	0.0019	33	1	0
positive regulation of cell death	0.0021	33	0	0
translation	0.0068	27	7	0
response to wounding	0.0078	36	0	0
intracellular signaling cascade	0.0083	65	0	0
immune system development	0.0094	24	0	0
leukocyte differentiation	0.0099	16	0	0
hemopoietic or lymphoid organ development	0.012	23	0	0
lymphocyte differentiation	0.014	14	0	0
defense response	0.014	39	2	0
regulation of protein kinase cascade	0.02	22	0	0
regulation of I-kappaB kinase/NF-kappaB cascade	0.022	14	2	0
hemopoiesis	0.03	21	2	0
inflammatory response	0.045	25	15	0
positive regulation of protein kinase cascade	0.047	17	0	0
sum		935	90	1

36 CD14+CD16+ monocytes vs. Macrophage - monocyte derived (down-regulated)

GO term	FDR	Counts at both methods	Counts at RECLU	Counts at original Paraclu
DNA packaging	0.00088	21	0	0
M phase	0.0014	38	0	0
immune effector process	0.002	22	0	0
mitosis	0.0028	29	14	4
nuclear division	0.0028	29	0	0
response to wounding	0.0037	51	1	0
M phase of mitotic cell cycle	0.0041	29	0	0
organelle fission	0.0063	29	0	0
protein-DNA complex assembly	0.0083	17	0	0
nucleosome organization	0.011	17	0	0
nucleosome assembly	0.013	16	4	0
chromatin assembly	0.021	16	0	0
cell cycle phase	0.023	41	0	0
sum		355	19	4

37 CD14+CD16+ monocytes vs. Neutrophils (up-regulated)

GO term	FDR	Counts at both methods	Counts at RECLU	Counts at original Paraclu
translation	2.1e-36	115	9	0
ncRNA metabolic process	2.1e-21	76	3	0
translational elongation	2.8e-18	46	1	0
ribosome biogenesis	5.2e-16	48	2	0
ribonucleoprotein complex biogenesis	8e-16	59	0	0
ncRNA processing	6.1e-15	59	0	0
RNA processing	9.8e-14	111	6	0
rRNA metabolic process	3.8e-12	38	0	0
rRNA processing	3.5e-11	36	5	0
tRNA metabolic process	3e-08	37	0	0
cellular respiration	2.1e-05	29	0	0
tRNA aminoacylation	0.00031	18	0	0
amino acid activation	0.00031	18	0	0
tRNA aminoacylation for protein translation	0.00031	18	3	0
oxidation reduction	0.0059	92	3	0
ribosomal large subunit biogenesis	0.0073	8	1	0
tRNA processing	0.012	21	5	0
cellular macromolecular complex subunit organization	0.016	58	0	0
intracellular protein transport	0.016	60	10	0
cellular macromolecular complex assembly	0.019	53	0	0
electron transport chain	0.031	26	4	0
cofactor metabolic process	0.032	37	0	0
energy derivation by oxidation of organic compounds	0.039	30	0	0
aerobic respiration	0.049	13	0	0
sum		1106	52	0

38 CD14+CD16+ monocytes vs. Neutrophils (down-regulated)

GO term	FDR	Counts at both methods	Counts at RECLU	Counts at original Paraclu
defense response	7.4e-08	62	2	0
inflammatory response	4.1e-07	41	6	3
immune response	2.9e-06	63	6	4
response to wounding	9.4e-06	52	0	0
protein amino acid phosphorylation	0.00019	57	1	2
intracellular signaling cascade	0.0004	88	0	0
protein kinase cascade	0.0014	37	2	0
leukocyte activation	0.0026	28	0	0
phosphorylation	0.0032	61	0	0
phosphorus metabolic process	0.014	68	0	0
phosphate metabolic process	0.014	68	0	0
cell activation	0.023	29	0	0
sum		654	17	9

39 CD14-CD16+ monocytes vs. CD14+ monocytes (up-regulated)

GO term	FDR	Counts at both methods	Counts at RECLU	Counts at original Paraclu
mitochondrial electron transport, NADH to ubiquinone	6.8e-05	13	2	0
respiratory electron transport chain	0.0015	14	6	0
cellular respiration	0.0016	17	2	0
mitochondrial ATP synthesis coupled electron transport	0.0021	13	0	0
ATP synthesis coupled electron transport	0.0021	13	0	0
oxidative phosphorylation	0.0091	16	2	1
translation	0.0093	32	7	0
electron transport chain	0.014	17	1	1
sum		135	20	2

40 CD14-CD16+ monocytes vs. CD14+ monocytes (down-regulated)

GO term	FDR	Counts at both methods	Counts at RECLU	Counts at original Paraclu
response to wounding	2.7e-10	69	0	1
immune response	3.4e-09	79	7	0
protein kinase cascade	2.5e-08	52	5	0
intracellular signaling cascade	4.7e-08	115	0	0
leukocyte activation	6.2e-08	40	0	0
regulation of apoptosis	1.8e-07	83	3	0
cell activation	2.5e-07	43	0	0
regulation of programmed cell death	2.9e-07	83	0	0
regulation of cell death	3.4e-07	83	0	0
lymphocyte activation	9.7e-07	34	0	0
inflammatory response	1.2e-06	45	13	0
positive regulation of immune system process	2.2e-06	37	0	0
hemopoiesis	6.3e-06	36	3	0
defense response	1.1e-05	65	1	0
regulation of phosphorylation	1.2e-05	54	0	0
positive regulation of cell proliferation	1.3e-05	50	15	3
negative regulation of apoptosis	1.7e-05	45	24	0
regulation of phosphate metabolic process	1.8e-05	55	0	0
regulation of phosphorus metabolic process	1.8e-05	55	0	0
regulation of cell proliferation	2.4e-05	76	7	0
regulation of cytokine production	2.4e-05	30	1	0
hemopoietic or lymphoid organ development	2.4e-05	37	0	0
negative regulation of programmed cell death	2.7e-05	45	0	0
negative regulation of cell death	2.9e-05	45	0	0
immune system development	3.6e-05	38	0	0
taxis	0.0001	27	0	0
chemotaxis	0.0001	27	4	0
anti-apoptosis	0.00013	31	0	0
leukocyte differentiation	0.00014	24	0	0
positive regulation of cell communication	0.00043	40	0	0
positive regulation of signal transduction	0.00062	37	0	0
lymphocyte differentiation	0.00085	20	0	0
cell migration	0.0011	35	5	2
protein amino acid phosphorylation	0.0011	63	4	0
positive regulation of developmental process	0.0012	35	0	0
regulation of lymphocyte activation	0.0013	24	0	0
regulation of transferase activity	0.0015	42	0	0
regulation of T cell activation	0.0015	21	0	0
positive regulation of locomotion	0.0018	19	0	0
death	0.002	66	1	0
positive regulation of smooth muscle cell proliferation	0.0022	11	1	0
regulation of leukocyte activation	0.003	25	0	0
cell death	0.0034	65	5	0
regulation of kinase activity	0.0034	40	0	0
regulation of protein kinase activity	0.0038	39	0	0
apoptosis	0.0041	57	20	1
regulation of mononuclear cell proliferation	0.0044	17	0	0
regulation of leukocyte proliferation	0.0044	17	0	0
negative regulation of molecular function	0.0046	38	0	0
MAPKKK cascade	0.0057	26	2	0
positive regulation of leukocyte activation	0.0059	19	0	0
negative regulation of transferase activity	0.0061	18	0	0
programmed cell death	0.0064	57	0	0
locomotory behavior	0.0072	33	2	0
regulation of cell activation	0.0076	25	0	0
regulation of protein kinase cascade	0.0077	31	0	0
positive regulation of cell motion	0.0081	18	2	0
positive regulation of cell migration	0.0096	17	4	0
positive regulation of cell activation	0.012	19	0	1
cell motility	0.012	35	0	0
localization of cell	0.012	35	0	0
cell motion	0.012	47	7	0
positive regulation of cell differentiation	0.012	29	2	1
regulation of smooth muscle cell proliferation	0.017	12	0	0
regulation of lymphocyte proliferation	0.018	16	0	0
T cell activation	0.019	20	0	0
positive regulation of leukocyte proliferation	0.023	13	0	0
positive regulation of mononuclear cell proliferation	0.023	13	0	0
negative regulation of macromolecule metabolic process	0.026	63	0	0
phosphorylation	0.029	67	2	0
regulation of binding	0.03	22	0	0
positive regulation of lymphocyte activation	0.03	17	0	0
negative regulation of protein kinase activity	0.032	16	0	0
cell proliferation	0.034	43	10	0
wound healing	0.034	25	3	1
positive regulation of multicellular organismal process	0.04	29	0	0
regulation of cell size	0.042	26	0	0
phosphorus metabolic process	0.045	77	0	0
phosphate metabolic process	0.045	77	0	0
enzyme linked receptor protein signaling pathway	0.048	36	0	0
positive regulation of cytokine production	0.048	16	0	0
negative regulation of kinase activity	0.048	16	1	0
regulation of T cell differentiation	0.049	12	1	0
sum		3239	155	10

41 CD14-CD16+ monocytes vs. CD19+ B cells (up-regulated)

GO term	FDR	Counts at both methods	Counts at RECLU	Counts at original Paraclu
immune response	0.00091	51	8	1
response to wounding	0.012	40	2	0
defense response	0.015	44	4	0
immune effector process	0.027	17	0	0
intracellular signaling cascade	0.035	72	0	0
inflammatory response	0.041	28	15	1
sum		252	29	2

42 CD14-CD16+ monocytes vs. CD19+ B cells (down-regulated)

GO term	FDR	Counts at both methods	Counts at RECLU	Counts at original Paraclu
regulation of T cell activation	5.1e-05	20	0	0
regulation of lymphocyte activation	0.00011	22	0	0
regulation of cell activation	0.00048	23	0	0
positive regulation of lymphocyte activation	0.00051	17	0	0
positive regulation of immune system process	0.00067	27	1	0
positive regulation of T cell activation	0.0007	15	1	0
regulation of leukocyte activation	0.0008	22	0	0
lymphocyte activation	0.0012	24	0	0
positive regulation of leukocyte activation	0.0018	17	0	0
regulation of transcription	0.0022	136	0	0
positive regulation of cell activation	0.0033	17	0	0
T cell activation	0.0041	18	1	0
chromatin organization	0.0093	33	0	0
leukocyte activation	0.01	25	0	0
phosphorus metabolic process	0.018	62	0	0
phosphate metabolic process	0.018	62	0	0
transcription	0.035	110	0	0
positive regulation of lymphocyte proliferation	0.036	11	0	0
apoptosis	0.043	43	27	2
positive regulation of mononuclear cell proliferation	0.043	11	0	0
positive regulation of leukocyte proliferation	0.043	11	0	0
protein amino acid phosphorylation	0.048	46	4	0
sum		772	34	2

43 CD14-CD16+ monocytes vs. CD34 cells differentiated to erythrocyte lineage (up-regulated)

GO term	FDR	Counts at both methods	Counts at RECLU	Counts at original Paraclu
immune response	6.1e-17	109	5	2
leukocyte activation	1.2e-07	45	1	0
regulation of programmed cell death	4.8e-07	97	0	0
intracellular signaling cascade	5.3e-07	133	0	0
regulation of cell death	5.8e-07	97	1	0
regulation of apoptosis	6.1e-07	96	2	0
positive regulation of apoptosis	3.1e-06	61	11	1
cell activation	3.3e-06	47	0	0
positive regulation of programmed cell death	4.1e-06	61	0	0
positive regulation of cell death	4.9e-06	61	0	0
lymphocyte activation	7.5e-06	37	0	0
positive regulation of lymphocyte differentiation	1.2e-05	15	0	0
defense response	1.3e-05	76	2	0
programmed cell death	2.2e-05	75	0	0
apoptosis	2.7e-05	74	16	1
positive regulation of T cell differentiation	3.5e-05	14	0	0
regulation of T cell differentiation	6.2e-05	17	0	0
inflammatory response	6.2e-05	48	5	4
immune effector process	6.5e-05	28	0	0
death	0.00016	82	0	0
regulation of alpha-beta T cell activation	0.00018	14	0	0
cell death	0.00024	81	11	0
positive regulation of alpha-beta T cell activation	0.00027	12	0	0
regulation of lymphocyte differentiation	0.00028	18	0	0
positive regulation of immune system process	0.00029	38	0	0
antigen processing and presentation of peptide antigen	0.00066	12	0	0
hemopoiesis	0.00068	37	0	0
positive regulation of cytokine production	0.00073	21	0	0
positive regulation of T cell activation	0.001	19	0	0
regulation of cytokine production	0.0011	31	0	0
T cell activation	0.0011	25	2	1
positive regulation of cell activation	0.0016	23	1	0
regulation of alpha-beta T cell differentiation	0.0019	11	0	0
positive regulation of alpha-beta T cell differentiation	0.002	10	0	0
actin cytoskeleton organization	0.002	35	4	1
leukocyte differentiation	0.0023	25	0	0
positive regulation of lymphocyte activation	0.0026	21	0	0
hemopoietic or lymphoid organ development	0.0027	38	0	0
protein kinase cascade	0.003	48	1	0
regulation of T cell activation	0.004	23	0	0
immune system development	0.0044	39	0	0
positive regulation of immune response	0.0044	26	0	0
myeloid leukocyte activation	0.0045	14	0	0
positive regulation of protein kinase cascade	0.0063	28	0	0
regulation of lymphocyte activation	0.0065	26	0	0
regulation of protein kinase cascade	0.0068	36	0	0
leukocyte mediated immunity	0.0069	19	0	0
actin filament-based process	0.0086	35	0	0
positive regulation of leukocyte activation	0.011	21	0	0
response to wounding	0.011	60	2	0
regulation of I-kappaB kinase/NF-kappaB cascade	0.013	21	0	0
regulation of cell activation	0.016	28	0	1
antigen processing and presentation	0.017	18	0	0
innate immune response	0.02	24	16	5
positive regulation of tumor necrosis factor production	0.024	8	1	0
regulation of specific transcription from RNA polymerase II promoter	0.026	19	0	0
induction of apoptosis	0.026	41	9	2
induction of programmed cell death	0.028	41	0	0
lymphocyte mediated immunity	0.033	16	0	0
positive regulation of response to stimulus	0.037	33	0	0
T cell proliferation	0.037	10	0	0
positive regulation of I-kappaB kinase/NF-kappaB cascade	0.04	19	6	1
positive regulation of MAPKKK cascade	0.044	13	0	0
regulation of actin cytoskeleton organization	0.045	18	0	0
sum		2428	96	19

44 CD14-CD16+ monocytes vs. CD34 cells differentiated to erythrocyte lineage (down-regulated)

GO term	FDR	Counts at both methods	Counts at RECLU	Counts at original Paraclu
protein-DNA complex assembly	3e-19	36	0	0
nucleosome organization	7.1e-19	36	0	0
nucleosome assembly	2.2e-18	34	4	0
DNA packaging	4.9e-18	39	1	0
chromatin assembly	8.2e-18	34	0	0
cell cycle	2.9e-17	104	11	0
chromosome organization	9.5e-17	78	1	0
chromatin assembly or disassembly	6.5e-14	36	0	0
cell cycle phase	8e-13	65	0	0
cell cycle process	3.3e-11	75	1	0
M phase	2.2e-10	53	0	0
mitotic cell cycle	5.6e-10	56	4	0
chromatin organization	1.4e-09	56	0	0
organelle fission	1.5e-09	42	0	0
cellular macromolecular complex subunit organization	5.7e-09	53	0	0
mitosis	7.4e-09	40	7	0
nuclear division	7.4e-09	40	0	0
cell division	9.6e-09	47	9	0
M phase of mitotic cell cycle	1.3e-08	40	0	0
cellular macromolecular complex assembly	3.9e-08	48	0	0
DNA metabolic process	2.5e-07	62	0	0
DNA replication	9.9e-06	32	4	0
chromosome segregation	2.3e-05	20	1	0
regulation of cell cycle	0.00014	42	1	0
DNA replication initiation	0.00062	9	1	0
macromolecular complex subunit organization	0.00067	68	0	0
microtubule-based process	0.00081	34	1	0
macromolecular complex assembly	0.0014	64	0	0
cytoskeleton organization	0.0026	47	2	1
response to DNA damage stimulus	0.0035	42	3	0
DNA-dependent DNA replication	0.008	14	0	0
negative regulation of organelle organization	0.021	16	0	0
microtubule cytoskeleton organization	0.025	22	2	0
DNA duplex unwinding	0.027	8	1	0
DNA geometric change	0.027	8	0	0
cellular response to stress	0.03	53	0	0
negative regulation of cellular component organization	0.049	21	0	0
sum		1574	54	1

45 CD14-CD16+ monocytes vs. CD4+CD25-CD45RA+ naive conventional T cell (up-regulated)

GO term	FDR	Counts at both methods	Counts at RECLU	Counts at original Paraclu
immune response	2e-07	73	6	2
response to wounding	2.6e-06	59	3	0
defense response	6.8e-06	64	2	0
inflammatory response	1.3e-05	42	6	2
immune effector process	2.5e-05	25	0	0
intracellular signaling cascade	3.7e-05	103	0	0
lymphocyte mediated immunity	0.0002	17	0	0
regulation of programmed cell death	0.00021	73	0	0
response to organic substance	0.00023	67	0	0
regulation of cell death	0.00024	73	1	0
positive regulation of apoptosis	0.00027	47	10	0
regulation of apoptosis	0.00031	72	6	0
positive regulation of programmed cell death	0.00033	47	0	0
positive regulation of cell death	0.00038	47	0	0
leukocyte mediated immunity	0.00075	18	0	0
leukocyte activation	0.0021	31	1	0
programmed cell death	0.0021	57	0	0
apoptosis	0.003	56	19	0
innate immune response	0.0033	22	23	4
cell death	0.0043	63	5	0
death	0.0054	63	0	0
cell activation	0.0093	33	0	0
immunoglobulin mediated immune response	0.011	13	1	0
lymphocyte activation	0.013	26	0	0
actin cytoskeleton organization	0.014	28	6	1
B cell mediated immunity	0.016	13	0	0
adaptive immune response	0.022	15	0	0
adaptive immune response based on somatic recombination of immune receptors built from immunoglobulin superfamily domains	0.022	15	0	0
protein kinase cascade	0.022	38	2	0
positive regulation of immune response	0.026	21	0	0
actin filament-based process	0.046	28	0	0
sum		1349	91	9

46 CD14-CD16+ monocytes vs. CD4+CD25-CD45RA+ naive conventional T cell (down-regulated)

GO term	FDR	Counts at both methods	Counts at RECLU	Counts at original Paraclu
regulation of T cell activation	0.00019	20	0	0
positive regulation of immune system process	0.00028	29	0	0
lymphocyte activation	0.00037	26	0	0
regulation of lymphocyte activation	0.0019	21	0	0
T cell activation	0.0029	19	1	0
lymphocyte differentiation	0.0036	17	0	0
leukocyte activation	0.0045	27	0	0
leukocyte differentiation	0.0052	19	0	0
regulation of cell activation	0.0073	22	0	0
hemopoiesis	0.009	26	0	2
protein amino acid phosphorylation	0.0095	51	2	0
regulation of leukocyte activation	0.012	21	0	0
hemopoietic or lymphoid organ development	0.017	27	0	0
phosphate metabolic process	0.034	65	0	0
phosphorus metabolic process	0.034	65	0	0
immune system development	0.048	27	0	0
sum		482	3	2

47 CD14-CD16+ monocytes vs. CD4+ T cells (up-regulated)

GO term	FDR	Counts at both methods	Counts at RECLU	Counts at original Paraclu
immune response	2.9e-07	73	5	2
response to wounding	9e-06	58	2	0
immune effector process	0.00013	24	0	0
intracellular signaling cascade	0.00021	101	0	0
lymphocyte mediated immunity	0.00022	17	0	0
leukocyte activation	0.00024	33	0	0
defense response	0.00068	59	1	0
leukocyte mediated immunity	0.00083	18	0	0
positive regulation of apoptosis	0.00087	46	9	1
positive regulation of programmed cell death	0.0011	46	0	0
positive regulation of cell death	0.0012	46	0	0
inflammatory response	0.0012	38	8	2
regulation of MAPKKK cascade	0.0014	20	1	0
cell activation	0.0014	35	0	0
lymphocyte activation	0.0014	28	0	0
regulation of programmed cell death	0.0025	70	0	0
regulation of cell death	0.0029	70	1	0
regulation of apoptosis	0.0036	69	7	0
protein kinase cascade	0.0043	40	0	0
immunoglobulin mediated immune response	0.011	13	1	0
programmed cell death	0.012	55	0	0
innate immune response	0.014	21	25	4
apoptosis	0.017	54	26	1
B cell mediated immunity	0.017	13	0	0
cell death	0.023	61	8	0
adaptive immune response based on somatic recombination of immune receptors built from immunoglobulin superfamily domains	0.024	15	0	0
adaptive immune response	0.024	15	0	0
positive regulation of kinase activity	0.024	28	0	0
response to organic substance	0.025	61	0	0
death	0.027	61	1	0
positive regulation of immune response	0.029	21	0	0
regulation of actin cytoskeleton organization	0.03	16	0	0
regulation of stress-activated protein kinase signaling pathway	0.032	14	0	0
regulation of protein kinase cascade	0.035	29	0	0
cell proliferation	0.039	42	10	1
regulation of actin filament-based process	0.046	16	0	0
positive regulation of transferase activity	0.049	28	1	0
sum		1454	106	11

48 CD14-CD16+ monocytes vs. CD4+ T cells (down-regulated)

GO term	FDR	Counts at both methods	Counts at RECLU	Counts at original Paraclu
lymphocyte activation	0.00019	30	0	0
chromatin organization	0.00077	43	1	0
nucleosome assembly	0.00079	18	7	0
T cell activation	0.0011	22	2	0
chromatin assembly or disassembly	0.0013	22	0	0
chromatin assembly	0.0013	18	0	0
protein-DNA complex assembly	0.0026	18	0	0
nucleosome organization	0.0036	18	0	0
leukocyte activation	0.0038	31	0	0
regulation of T cell activation	0.0058	20	0	0
protein amino acid phosphorylation	0.0089	60	5	0
lymphocyte differentiation	0.015	18	0	0
regulation of lymphocyte activation	0.016	22	0	0
hemopoietic or lymphoid organ development	0.044	30	0	0
cell activation	0.045	32	0	0
sum		402	15	0

49 CD14-CD16+ monocytes vs. CD8+ T cells (up-regulated)

GO term	FDR	Counts at both methods	Counts at RECLU	Counts at original Paraclu
immune response	1.1e-06	71	11	3
response to wounding	5.7e-06	58	1	0
immune effector process	0.0001	24	0	0
intracellular signaling cascade	0.00011	101	0	0
lymphocyte mediated immunity	0.00018	17	0	0
leukocyte activation	0.00018	33	0	0
positive regulation of apoptosis	0.00062	46	10	0
leukocyte mediated immunity	0.0007	18	0	0
positive regulation of programmed cell death	0.00075	46	0	0
positive regulation of cell death	0.00085	46	0	0
inflammatory response	0.00091	38	12	2
defense response	0.001	58	1	0
cell activation	0.0011	35	0	0
lymphocyte activation	0.0011	28	0	0
regulation of programmed cell death	0.0016	70	0	0
regulation of cell death	0.0018	70	1	0
regulation of apoptosis	0.0023	69	7	0
programmed cell death	0.004	56	0	0
regulation of MAPKKK cascade	0.005	19	1	0
apoptosis	0.0055	55	26	0
cell death	0.0076	62	15	0
protein kinase cascade	0.008	39	2	0
death	0.0094	62	1	0
immunoglobulin mediated immune response	0.01	13	1	0
innate immune response	0.011	21	28	4
B cell mediated immunity	0.015	13	0	0
response to organic substance	0.017	61	0	0
positive regulation of kinase activity	0.02	28	0	0
adaptive immune response based on somatic recombination of immune receptors built from immunoglobulin superfamily domains	0.021	15	0	0
adaptive immune response	0.021	15	0	0
positive regulation of immune response	0.024	21	0	0
regulation of stress-activated protein kinase signaling pathway	0.028	14	0	0
cell proliferation	0.03	42	16	1
positive regulation of transferase activity	0.039	28	1	0
sum		1392	134	10

50 CD14-CD16+ monocytes vs. CD8+ T cells (down-regulated)

GO term	FDR	Counts at both methods	Counts at RECLU	Counts at original Paraclu
lymphocyte activation	0.00019	30	0	0
chromatin organization	0.00077	43	0	0
nucleosome assembly	0.00079	18	9	0
T cell activation	0.0011	22	4	0
chromatin assembly or disassembly	0.0013	22	0	0
chromatin assembly	0.0013	18	0	0
protein-DNA complex assembly	0.0026	18	0	0
nucleosome organization	0.0036	18	0	0
leukocyte activation	0.0038	31	0	0
protein amino acid phosphorylation	0.0043	61	4	0
regulation of T cell activation	0.0058	20	0	0
lymphocyte differentiation	0.015	18	0	0
regulation of lymphocyte activation	0.016	22	0	0
positive regulation of immune system process	0.023	29	0	0
hemopoietic or lymphoid organ development	0.044	30	0	0
cell activation	0.045	32	1	0
sum		432	18	0

51 CD14-CD16+ monocytes vs. Macrophage - monocyte derived (up-regulated)

GO term	FDR	Counts at both methods	Counts at RECLU	Counts at original Paraclu
immune response	7e-07	74	7	2
inflammatory response	0.00043	40	10	4
leukocyte activation	0.0006	33	1	0
intracellular signaling cascade	0.00076	102	0	0
immune effector process	0.0011	23	0	0
defense response	0.0011	60	1	0
cell death	0.0012	67	8	1
response to wounding	0.0012	54	1	0
regulation of programmed cell death	0.0013	73	0	0
death	0.0015	67	1	0
regulation of cell death	0.0015	73	0	0
regulation of apoptosis	0.0019	72	2	1
positive regulation of apoptosis	0.0026	46	10	1
lymphocyte activation	0.003	28	0	0
positive regulation of programmed cell death	0.0031	46	0	0
cell activation	0.0034	35	0	0
positive regulation of cell death	0.0035	46	0	0
programmed cell death	0.009	57	0	0
regulation of MAPKKK cascade	0.01	19	1	0
regulation of actin cytoskeleton organization	0.011	17	1	0
apoptosis	0.012	56	23	3
regulation of actin polymerization or depolymerization	0.012	14	0	0
regulation of protein complex assembly	0.013	17	0	0
regulation of actin filament polymerization	0.017	13	0	0
regulation of actin filament-based process	0.017	17	0	0
regulation of actin filament length	0.017	14	0	0
cell proliferation	0.02	44	10	2
protein kinase cascade	0.026	39	7	0
positive regulation of T cell differentiation	0.03	10	0	0
actin cytoskeleton organization	0.033	28	5	1
regulation of stress-activated protein kinase signaling pathway	0.049	14	0	0
sum		1298	88	15

52 CD14-CD16+ monocytes vs. Macrophage - monocyte derived (down-regulated)

GO term	FDR	Counts at both methods	Counts at RECLU	Counts at original Paraclu
leukocyte activation	8.9e-05	36	0	0
lymphocyte activation	9.3e-05	32	0	0
hemopoietic or lymphoid organ development	0.00018	37	0	0
hemopoiesis	0.00048	34	6	1
lymphocyte differentiation	0.00062	21	0	0
cell activation	0.00074	38	0	0
immune system development	0.0008	37	0	0
leukocyte differentiation	0.0022	23	0	0
protein amino acid phosphorylation	0.0038	65	4	1
T cell activation	0.0044	22	7	0
nucleosome organization	0.011	18	0	0
nucleosome assembly	0.012	17	8	0
phosphorus metabolic process	0.013	84	0	0
phosphate metabolic process	0.013	84	0	1
chromatin assembly	0.019	17	1	0
regulation of T cell activation	0.019	20	0	0
protein-DNA complex assembly	0.034	17	0	0
intracellular signaling cascade	0.045	100	0	0
sum		702	26	3

53 CD14-CD16+ monocytes vs. Neutrophils (up-regulated)

GO term	FDR	Counts at both methods	Counts at RECLU	Counts at original Paraclu
translation	3.2e-37	114	8	1
ncRNA metabolic process	1.1e-21	75	0	0
ribonucleoprotein complex biogenesis	4.5e-18	61	0	0
translational elongation	5.4e-18	45	2	0
ribosome biogenesis	1.6e-17	49	2	0
RNA processing	1.8e-14	110	2	0
ncRNA processing	2.5e-14	57	0	0
rRNA metabolic process	2e-13	39	0	0
rRNA processing	1.6e-12	37	4	0
tRNA metabolic process	4.8e-08	36	0	0
tRNA aminoacylation	2.6e-05	19	0	0
amino acid activation	2.6e-05	19	0	0
tRNA aminoacylation for protein translation	2.6e-05	19	1	0
cellular respiration	4.1e-05	28	0	0
cellular macromolecular complex assembly	0.003	54	0	0
cellular macromolecular complex subunit organization	0.0049	58	0	0
cofactor metabolic process	0.0054	38	0	0
ribosomal large subunit biogenesis	0.0056	8	1	0
generation of precursor metabolites and energy	0.0091	52	2	1
intracellular protein transport	0.021	58	4	0
oxidation reduction	0.024	87	1	0
cellular protein localization	0.024	62	0	0
cellular macromolecule localization	0.03	62	0	0
aerobic respiration	0.034	13	0	0
sum		1200	27	2

54 CD14-CD16+ monocytes vs. Neutrophils (down-regulated)

GO term	FDR	Counts at both methods	Counts at RECLU	Counts at original Paraclu
defense response	5.5e-08	62	1	0
inflammatory response	8.9e-08	42	3	3
immune response	1.1e-07	66	4	3
response to wounding	9e-07	54	0	0
intracellular signaling cascade	0.00014	89	0	0
protein kinase cascade	0.0012	37	1	0
protein amino acid phosphorylation	0.02	51	0	2
sum		401	9	8

55 CD14+ monocytes vs. CD19+ B cells (up-regulated)

GO term	FDR	Counts at both methods	Counts at RECLU	Counts at original Paraclu
response to wounding	1.4e-17	81	0	0
immune response	1.4e-12	85	15	0
inflammatory response	3.4e-12	54	11	0
defense response	5.4e-11	76	2	0
regulation of cell proliferation	1.7e-08	83	4	0
cell activation	4.3e-07	42	0	0
leukocyte activation	1.8e-06	37	0	0
anti-apoptosis	5.2e-06	33	0	0
positive regulation of immune system process	1.5e-05	35	0	0
positive regulation of developmental process	2.3e-05	38	0	0
negative regulation of apoptosis	2.5e-05	44	30	1
negative regulation of programmed cell death	3.8e-05	44	0	0
negative regulation of cell death	4.1e-05	44	0	0
positive regulation of cell proliferation	4.5e-05	48	16	0
regulation of cytokine production	5.6e-05	29	0	0
intracellular signaling cascade	6.6e-05	103	0	0
positive regulation of cell differentiation	7.2e-05	33	2	1
lymphocyte activation	0.00012	30	0	0
hemopoiesis	0.00015	33	1	0
regulation of apoptosis	0.00022	73	6	0
protein kinase cascade	0.00025	43	5	0
regulation of programmed cell death	0.00033	73	0	0
regulation of cell death	0.00038	73	0	0
hemopoietic or lymphoid organ development	0.00045	34	0	0
immune system development	0.0006	35	0	0
wound healing	0.00065	28	3	1
positive regulation of multicellular organismal process	0.001	32	0	0
positive regulation of cell communication	0.0018	38	0	0
positive regulation of immune response	0.0024	23	0	0
positive regulation of response to stimulus	0.0046	30	0	0
lymphocyte proliferation	0.0052	12	0	0
positive regulation of signal transduction	0.0077	34	0	0
regulation of phosphorylation	0.0085	46	0	0
leukocyte proliferation	0.0085	12	0	0
mononuclear cell proliferation	0.0085	12	0	0
regulation of phosphorus metabolic process	0.011	47	0	0
regulation of phosphate metabolic process	0.011	47	0	0
positive regulation of defense response	0.013	15	0	0
T cell activation	0.014	20	2	0
cell proliferation	0.019	43	18	0
leukocyte differentiation	0.024	20	0	0
response to bacterium	0.028	25	1	0
regulation of transferase activity	0.031	38	0	0
positive regulation of cell activation	0.033	18	1	0
response to cytokine stimulus	0.033	15	1	1
regulation of protein kinase activity	0.035	36	0	0
positive regulation of cytokine production	0.036	16	2	0
regulation of protein kinase cascade	0.037	29	0	0
regulation of response to external stimulus	0.037	22	0	0
regulation of T cell differentiation	0.039	12	0	0
mesenchymal cell development	0.039	12	0	0
mesenchymal cell differentiation	0.039	12	0	0
positive regulation of protein kinase activity	0.039	27	0	0
taxis	0.041	22	0	0
chemotaxis	0.041	22	8	0
positive regulation of cellular biosynthetic process	0.045	58	0	0
mesenchyme development	0.047	12	0	0
sum		2138	128	4

56 CD14+ monocytes vs. CD19+ B cells (down-regulated)

GO term	FDR	Counts at both methods	Counts at RECLU	Counts at original Paraclu
regulation of T cell activation	0.0023	15	0	0
regulation of alpha-beta T cell activation	0.004	9	0	0
lymphocyte activation	0.0046	19	0	0
regulation of lymphocyte differentiation	0.0058	11	0	0
regulation of T cell differentiation	0.0072	10	0	0
leukocyte activation	0.019	20	0	0
T cell activation	0.028	14	3	1
positive regulation of T cell activation	0.032	11	1	2
regulation of lymphocyte activation	0.036	15	0	0
leukocyte differentiation	0.042	14	0	0
sum		138	4	3

57 CD14+ monocytes vs. CD34 cells differentiated to erythrocyte lineage (up-regulated)

GO term	FDR	Counts at both methods	Counts at RECLU	Counts at original Paraclu
immune response	1.6e-22	151	7	1
leukocyte activation	4.1e-18	74	0	0
cell activation	1.9e-16	79	0	0
lymphocyte activation	1.6e-14	61	0	0
intracellular signaling cascade	4.1e-13	200	0	0
regulation of apoptosis	8.2e-13	144	1	1
regulation of programmed cell death	2.1e-12	144	0	0
regulation of cell death	2.9e-12	144	0	0
T cell activation	6.6e-12	44	0	0
positive regulation of immune system process	1.3e-11	63	0	0
regulation of T cell differentiation	2.6e-11	27	0	0
leukocyte differentiation	3.1e-11	44	0	0
hemopoiesis	1.2e-10	61	4	0
regulation of lymphocyte differentiation	2.1e-10	29	1	0
regulation of T cell activation	3e-10	40	0	0
regulation of lymphocyte activation	8.1e-10	45	0	0
hemopoietic or lymphoid organ development	9.9e-10	63	0	0
immune system development	1.6e-09	65	0	0
lymphocyte differentiation	1.5e-08	35	0	0
positive regulation of lymphocyte differentiation	2.2e-08	20	0	0
response to wounding	2.8e-08	97	0	1
regulation of cell activation	2.9e-08	47	0	0
T cell differentiation	3e-08	27	2	0
positive regulation of cell activation	3.2e-08	36	0	1
positive regulation of T cell differentiation	3.5e-08	19	0	0
positive regulation of T cell activation	5.2e-08	29	2	0
defense response	5.3e-08	107	2	0
positive regulation of cell proliferation	5.7e-08	81	9	1
regulation of leukocyte activation	6e-08	45	0	0
protein kinase cascade	6e-08	75	5	0
positive regulation of lymphocyte activation	6.1e-08	33	0	0
regulation of cytokine production	1e-07	47	0	0
positive regulation of apoptosis	1.6e-07	82	8	1
positive regulation of leukocyte activation	1.8e-07	34	0	0
positive regulation of programmed cell death	2.3e-07	82	0	0
positive regulation of cell death	2.9e-07	82	0	1
inflammatory response	3.7e-07	67	7	1
regulation of alpha-beta T cell activation	4.7e-07	19	0	0
positive regulation of cell differentiation	5.1e-07	53	2	1
positive regulation of developmental process	5.7e-07	60	0	0
regulation of cell proliferation	9.3e-07	124	3	0
positive regulation of signal transduction	2.3e-06	61	0	0
anti-apoptosis	3.1e-06	48	0	0
apoptosis	3.5e-06	100	23	3
regulation of protein kinase cascade	4.1e-06	54	0	0
positive regulation of cell communication	4.3e-06	65	0	0
regulation of alpha-beta T cell differentiation	6.9e-06	15	0	0
programmed cell death	7.8e-06	100	0	0
immune effector process	9.4e-06	36	0	0
positive regulation of alpha-beta T cell activation	1.4e-05	15	0	0
negative regulation of apoptosis	1.4e-05	67	23	0
positive regulation of cytokine production	2.2e-05	28	0	0
regulation of phosphate metabolic process	2.6e-05	83	0	0
regulation of phosphorus metabolic process	2.6e-05	83	0	0
negative regulation of programmed cell death	2.6e-05	67	0	0
negative regulation of cell death	2.9e-05	67	0	0
positive regulation of alpha-beta T cell differentiation	3.2e-05	13	1	0
positive regulation of protein kinase cascade	3.8e-05	40	0	0
regulation of phosphorylation	4.2e-05	80	1	0
regulation of transferase activity	4.6e-05	68	0	0
positive regulation of kinase activity	0.00014	48	0	0
death	0.00015	109	1	0
positive regulation of transferase activity	0.00018	49	0	0
protein amino acid phosphorylation	0.00019	102	2	1
cell death	0.00019	108	11	0
regulation of kinase activity	0.00026	64	0	0
regulation of I-kappaB kinase/NF-kappaB cascade	0.0003	29	0	0
taxis	0.00036	37	0	0
chemotaxis	0.00036	37	6	0
regulation of mononuclear cell proliferation	0.00039	25	0	0
regulation of leukocyte proliferation	0.00039	25	0	0
lymphocyte proliferation	0.00055	17	0	0
positive regulation of immune response	0.00088	34	0	0
leukocyte proliferation	0.0012	17	0	0
mononuclear cell proliferation	0.0012	17	0	0
regulation of lymphocyte proliferation	0.0013	24	0	0
induction of apoptosis	0.0017	57	11	1
positive regulation of response to stimulus	0.0018	46	0	0
induction of programmed cell death	0.0019	57	0	0
positive regulation of multicellular organismal process	0.002	47	0	0
regulation of CD4-positive, alpha beta T cell differentiation	0.0021	10	0	0
myeloid leukocyte activation	0.0023	17	0	0
positive regulation of protein kinase activity	0.0023	44	0	0
regulation of cytokine biosynthetic process	0.0025	22	0	0
leukocyte mediated immunity	0.0025	24	0	0
T cell selection	0.0027	11	0	0

negative regulation of macromolecule metabolic process	0.0031	105	0	0
regulation of protein kinase activity	0.0042	59	0	0
negative regulation of macromolecule biosynthetic process	0.0046	83	0	0
negative regulation of biosynthetic process	0.0047	86	0	0
regulation of small GTPase mediated signal transduction	0.0049	47	5	0
enzyme linked receptor protein signaling pathway	0.0067	58	0	0
positive regulation of I-kappaB kinase/NF-kappaB cascade	0.0067	25	8	1
negative regulation of cellular biosynthetic process	0.007	84	0	0
positive regulation of cellular biosynthetic process	0.0073	98	0	0
positive regulation of macromolecule metabolic process	0.0075	117	0	0
positive regulation of biosynthetic process	0.0078	99	0	0
positive regulation of mononuclear cell proliferation	0.0092	18	0	0
positive regulation of leukocyte proliferation	0.0092	18	0	0
phosphate metabolic process	0.01	129	0	0
phosphorus metabolic process	0.01	129	0	0
positive regulation of molecular function	0.012	86	0	0
regulation of smooth muscle cell proliferation	0.012	16	0	0
response to organic substance	0.013	101	1	0
positive regulation of smooth muscle cell proliferation	0.014	13	2	0
positive regulation of macromolecule biosynthetic process	0.017	93	1	0
regulation of Ras protein signal transduction	0.018	40	0	0
antigen processing and presentation	0.019	22	2	0
actin cytoskeleton organization	0.02	42	9	2
positive regulation of CD4-positive, alpha beta T cell differentiation	0.021	8	0	0
positive regulation of cytokine biosynthetic process	0.022	16	0	0
antigen processing and presentation of peptide antigen	0.028	12	0	0
positive regulation of lymphocyte proliferation	0.032	17	0	0
cell proliferation	0.032	67	9	0
angiogenesis	0.034	31	11	2
regulation of cellular component size	0.036	47	0	0
regulation of cellular protein metabolic process	0.042	71	0	0
cell motion	0.045	71	5	0
sum		6854	185	19

58 CD14+ monocytes vs. CD34 cells differentiated to erythrocyte lineage (down-regulated)

GO term	FDR	Counts at both methods	Counts at RECLU	Counts at original Paraclu
chromosome organization	1e-17	96	0	0
DNA packaging	1.2e-16	43	0	0
protein-DNA complex assembly	1.5e-16	38	0	0
nucleosome assembly	5.6e-16	36	1	0
cell cycle	1.5e-15	124	7	1
chromatin assembly	2.2e-15	36	0	0
nucleosome organization	3.2e-15	37	0	0
cellular macromolecular complex subunit organization	6e-13	72	0	0
cellular macromolecular complex assembly	8e-13	67	0	0
DNA metabolic process	1e-12	89	0	0
cell cycle process	4.1e-11	92	1	0
chromatin assembly or disassembly	4.9e-11	38	0	0
mitotic cell cycle	5.2e-11	70	6	0
M phase	6.1e-11	65	0	0
cell cycle phase	1.8e-10	74	0	0
organelle fission	4.4e-10	51	0	0
mitosis	1.4e-09	49	10	0
nuclear division	1.4e-09	49	0	0
M phase of mitotic cell cycle	2.8e-09	49	0	0
chromatin organization	1.4e-08	66	0	0
macromolecular complex subunit organization	2.3e-08	100	0	0
DNA replication	2.4e-08	43	5	0
macromolecular complex assembly	3.7e-08	95	0	0
cell division	7.5e-08	55	10	1
chromosome segregation	1.2e-06	25	2	0
response to DNA damage stimulus	1.4e-06	61	4	1
DNA repair	4.9e-06	50	9	0
DNA replication initiation	2.5e-05	11	0	0
cellular response to stress	3.5e-05	77	1	0
nitrogen compound biosynthetic process	0.0057	47	0	0
regulation of cell cycle	0.0094	47	1	1
translation	0.0094	47	9	0
microtubule-based process	0.01	39	2	0
DNA-dependent DNA replication	0.012	16	0	0
double-strand break repair	0.03	16	2	0
microtubule cytoskeleton organization	0.049	26	4	0
sum		1996	74	4

59 CD14+ monocytes vs. CD4+CD25-CD45RA+ naive conventional T cell (up-regulated)

GO term	FDR	Counts at both methods	Counts at RECLU	Counts at original Paraclu
response to wounding	1.6e-14	96	0	1
intracellular signaling cascade	6.1e-13	167	0	0
regulation of apoptosis	1.5e-11	119	4	0
regulation of programmed cell death	3.2e-11	119	0	0
regulation of cell death	4.1e-11	119	0	0
cell activation	3.6e-10	59	0	0
protein kinase cascade	3.7e-10	69	4	0
inflammatory response	8e-10	63	11	0
leukocyte activation	2e-09	52	0	0
regulation of cell proliferation	8.3e-09	110	4	0
defense response	1.5e-08	92	1	0
regulation of phosphorylation	2.1e-08	76	1	0
regulation of phosphate metabolic process	2.2e-08	78	0	0
regulation of phosphorus metabolic process	2.2e-08	78	0	0
immune response	2.6e-08	99	13	0
positive regulation of apoptosis	4.3e-07	69	6	1
regulation of cytokine production	4.8e-07	40	0	0
positive regulation of programmed cell death	5.9e-07	69	0	0
positive regulation of cell death	7.2e-07	69	1	1
regulation of transferase activity	8.7e-07	62	0	0
positive regulation of developmental process	1.3e-06	51	0	0
positive regulation of cell proliferation	1.5e-06	66	14	1
lymphocyte activation	2.5e-06	41	0	0
regulation of kinase activity	3.4e-06	59	0	0
regulation of cellular protein metabolic process	4.9e-06	71	0	0
anti-apoptosis	7.2e-06	41	0	0
positive regulation of kinase activity	7.2e-06	44	0	0
response to organic substance	1.5e-05	94	2	0
negative regulation of apoptosis	1.7e-05	57	22	0
positive regulation of immune system process	1.8e-05	44	0	0
apoptosis	2.2e-05	82	31	1
regulation of protein amino acid phosphorylation	2.2e-05	36	1	0
positive regulation of transferase activity	2.4e-05	44	0	0
negative regulation of programmed cell death	2.9e-05	57	0	0
negative regulation of cell death	3.2e-05	57	0	0
MAPKKK cascade	3.5e-05	37	4	0
programmed cell death	4.3e-05	82	0	0
regulation of protein kinase activity	4.6e-05	55	0	0
positive regulation of cell differentiation	5.3e-05	42	3	1
regulation of protein kinase cascade	7.3e-05	44	0	0
immune effector process	8.5e-05	30	0	0
hemopoietic or lymphoid organ development	9.3e-05	45	0	0
positive regulation of multicellular organismal process	0.00011	43	0	0
hemopoiesis	0.00013	42	2	0
positive regulation of cell communication	0.00016	52	0	0
immune system development	0.00021	46	0	0
positive regulation of signal transduction	0.00022	48	0	0
positive regulation of protein kinase activity	0.00022	40	0	0
positive regulation of macromolecule metabolic process	0.00033	102	0	0
positive regulation of biosynthetic process	0.00036	87	0	0
positive regulation of cellular biosynthetic process	0.00038	86	0	0
cell death	0.00042	89	10	0
death	0.00056	89	2	0
small GTPase mediated signal transduction	0.0006	48	22	0
leukocyte mediated immunity	0.00072	22	0	0
cell motion	0.00076	65	5	0
enzyme linked receptor protein signaling pathway	0.0013	51	0	0
chemotaxis	0.0014	31	8	0
taxis	0.0014	31	0	0
regulation of protein modification process	0.0014	46	0	0
positive regulation of cytokine production	0.0016	22	1	0
wound healing	0.0027	34	4	1
T cell activation	0.0039	26	2	0
regulation of cytokine biosynthetic process	0.0051	19	0	0
positive regulation of immune response	0.0056	28	0	0
positive regulation of macromolecule biosynthetic process	0.0058	79	1	0
negative regulation of macromolecule metabolic process	0.0068	86	0	0
positive regulation of response to stimulus	0.0071	38	0	0
positive regulation of phosphorus metabolic process	0.0099	22	0	0
positive regulation of phosphate metabolic process	0.0099	22	0	0
regulation of MAPKKK cascade	0.012	23	2	0
protein amino acid phosphorylation	0.012	79	4	0
phosphate metabolic process	0.013	106	0	0
phosphorus metabolic process	0.013	106	0	0
response to molecule of bacterial origin	0.013	20	1	0
induction of apoptosis	0.013	46	13	1
induction of programmed cell death	0.014	46	0	0
positive regulation of molecular function	0.018	71	0	0
negative regulation of molecular function	0.018	47	0	0
positive regulation of nitrogen compound metabolic process	0.021	76	0	0
positive regulation of protein amino acid phosphorylation	0.021	20	1	1

regulation of smooth muscle cell proliferation	0.022	14	0	0
positive regulation of phosphorylation	0.022	21	1	0
positive regulation of gene expression	0.025	70	1	1
response to bacterium	0.026	32	2	0
leukocyte differentiation	0.026	25	0	0
positive regulation of protein kinase cascade	0.03	29	0	0
regulation of cellular component size	0.031	40	0	0
positive regulation of catalytic activity	0.032	64	2	0
regulation of small GTPase mediated signal transduction	0.033	38	9	0
regulation of stress-activated protein kinase signaling pathway	0.035	17	0	0
negative regulation of phosphate metabolic process	0.036	14	0	0
negative regulation of phosphorus metabolic process	0.036	14	0	0
response to lipopolysaccharide	0.039	18	4	0
cell migration	0.048	40	2	2
sum		5354	221	12

60 CD14+ monocytes vs. CD4+CD25-CD45RA+ naive conventional T cell (down-regulated)

GO term	FDR	Counts at both methods	Counts at RECLU	Counts at original Paraclu
translational elongation	0.00021	19	8	2
leukocyte differentiation	0.041	18	0	0
sum		37	8	2

61 CD14+ monocytes vs. CD4+ T cells (up-regulated)

GO term	FDR	Counts at both methods	Counts at RECLU	Counts at original Paraclu
response to wounding	2.2e-15	82	0	1
inflammatory response	2.4e-10	54	15	0
regulation of cell proliferation	4e-10	93	7	0
defense response	5e-10	79	1	0
immune response	1.5e-09	84	8	0
regulation of apoptosis	3.6e-09	92	5	0
regulation of programmed cell death	6.2e-09	92	0	0
regulation of cell death	7.6e-09	92	0	0
cell activation	2e-08	47	0	0
leukocyte activation	4.5e-08	42	0	0
positive regulation of developmental process	3.1e-07	44	0	0
regulation of cytokine production	5.7e-07	34	0	0
positive regulation of cell proliferation	1e-06	55	13	1
positive regulation of multicellular organismal process	3e-06	39	0	0
intracellular signaling cascade	3.1e-06	116	0	0
protein kinase cascade	4e-06	50	6	0
positive regulation of immune system process	5.1e-06	38	0	0
regulation of phosphate metabolic process	6.6e-06	59	0	0
regulation of phosphorus metabolic process	6.6e-06	59	0	0
lymphocyte activation	7.1e-06	34	0	0
negative regulation of apoptosis	7.8e-06	48	23	1
regulation of phosphorylation	1e-05	57	0	0
negative regulation of programmed cell death	1.2e-05	48	1	0
negative regulation of cell death	1.3e-05	48	0	0
anti-apoptosis	1.7e-05	34	0	0
positive regulation of cell differentiation	2.2e-05	36	3	2
positive regulation of apoptosis	3e-05	53	5	1
positive regulation of programmed cell death	3.7e-05	53	0	0
positive regulation of cell death	4.4e-05	53	0	1
regulation of transferase activity	0.0001	47	0	0
regulation of cellular protein metabolic process	0.00012	55	0	0
positive regulation of cell communication	0.00016	43	0	0
regulation of protein amino acid phosphorylation	0.00018	29	1	0
positive regulation of transferase activity	0.00023	35	0	0
positive regulation of kinase activity	0.0003	34	0	0
positive regulation of signal transduction	0.00053	39	1	0
positive regulation of macromolecule metabolic process	0.00059	81	0	0
regulation of kinase activity	0.0006	44	0	0
response to organic substance	0.0008	71	2	0
positive regulation of protein kinase activity	0.0013	32	0	0
hemopoiesis	0.0015	33	0	0
positive regulation of response to stimulus	0.0015	33	0	0
wound healing	0.0015	29	6	1
positive regulation of cytokine production	0.0015	19	2	0
regulation of protein kinase activity	0.0016	42	0	0
regulation of MAPKKK cascade	0.0016	21	2	0
regulation of protein kinase cascade	0.0017	34	0	0
positive regulation of biosynthetic process	0.0018	68	0	0
regulation of cytokine biosynthetic process	0.0021	17	0	0
positive regulation of cellular biosynthetic process	0.0023	67	0	0
immune effector process	0.0033	23	0	0
positive regulation of immune response	0.0036	24	0	0
regulation of protein modification process	0.004	37	0	0
regulation of stress-activated protein kinase signaling pathway	0.0042	16	0	0
T cell activation	0.0045	22	2	0
hemopoietic or lymphoid organ development	0.0045	34	0	0
response to cytokine stimulus	0.0052	17	1	1
response to bacterium	0.0059	28	2	0
immune system development	0.0062	35	0	0
positive regulation of phosphorus metabolic process	0.0076	19	0	0
positive regulation of phosphate metabolic process	0.0076	19	0	0
positive regulation of cell activation	0.0089	20	1	1
regulation of cell activation	0.0093	26	0	0
regulation of JNK cascade	0.01	15	0	0
apoptosis	0.01	59	25	0
programmed cell death	0.016	59	0	0
response to molecule of bacterial origin	0.017	17	0	0
positive regulation of macromolecule biosynthetic process	0.017	62	1	0
regulation of lymphocyte activation	0.018	23	0	0
regulation of T cell differentiation	0.018	13	0	0
chemotaxis	0.02	24	8	0
taxis	0.02	24	0	0
enzyme linked receptor protein signaling pathway	0.02	39	0	0
positive regulation of phosphorylation	0.02	18	2	0
response to nutrient	0.024	22	2	0
positive regulation of protein amino acid phosphorylation	0.026	17	2	1
leukocyte differentiation	0.03	21	0	0
developmental maturation	0.035	18	0	0
regulation of lymphocyte differentiation	0.036	14	0	0
regulation of smooth muscle cell proliferation	0.036	12	0	0
regulation of leukocyte activation	0.036	24	0	0
cell migration	0.042	33	1	2

cell death	0.044	65	7	0
cell chemotaxis	0.045	11	1	0
regulation of small GTPase mediated signal transduction	0.045	31	7	0
cell proliferation	0.047	45	18	0
sum		3574	181	13

62 CD14+ monocytes vs. CD4+ T cells (down-regulated)

GO term	FDR	Counts at both methods	Counts at RECLU	Counts at original Paraclu
nucleosome assembly	5e-12	23	5	2
chromatin assembly	1.1e-11	23	0	0
protein-DNA complex assembly	3.1e-11	23	0	0
nucleosome organization	5.1e-11	23	0	0
chromatin assembly or disassembly	6.9e-10	25	0	0
DNA packaging	9e-10	24	0	0
lymphocyte activation	4.3e-07	27	0	0
chromatin organization	1.4e-06	37	1	0
T cell activation	1.1e-05	20	2	0
leukocyte activation	2.9e-05	27	0	0
chromosome organization	3.3e-05	40	1	0
lymphocyte differentiation	0.00011	17	0	0
regulation of T cell activation	0.00012	18	0	0
cellular macromolecular complex assembly	0.00059	29	0	0
leukocyte differentiation	0.00064	18	0	0
regulation of lymphocyte activation	0.00078	19	0	0
cell activation	0.0009	27	0	0
regulation of cell activation	0.0022	20	0	0
regulation of leukocyte activation	0.0042	19	0	0
cellular macromolecular complex subunit organization	0.0058	29	0	0
hemopoiesis	0.015	22	3	1
immune system development	0.017	24	0	0
hemopoietic or lymphoid organ development	0.02	23	0	0
sum		557	12	3

63 CD14+ monocytes vs. CD8+ T cells (up-regulated)

GO term	FDR	Counts at both methods	Counts at RECLU	Counts at original Paraclu
response to wounding	5.9e-18	85	0	1
inflammatory response	1.8e-11	55	16	0
regulation of cell proliferation	6e-11	93	6	0
defense response	6.2e-09	75	1	0
regulation of apoptosis	2.4e-08	88	3	0
regulation of programmed cell death	4e-08	88	0	0
regulation of cell death	4.9e-08	88	0	0
positive regulation of cell proliferation	3.2e-07	55	14	1
positive regulation of developmental process	4.1e-07	43	0	0
immune response	5.8e-07	76	7	0
positive regulation of immune system process	5.9e-07	39	0	0
regulation of cytokine production	4.5e-06	32	0	0
intracellular signaling cascade	7.7e-06	112	0	0
cell activation	1.2e-05	41	0	0
leukocyte activation	1.2e-05	37	0	0
wound healing	1.7e-05	32	5	1
positive regulation of apoptosis	2.8e-05	52	5	1
positive regulation of cell differentiation	3.4e-05	35	3	2
protein kinase cascade	3.5e-05	47	5	0
positive regulation of programmed cell death	3.5e-05	52	0	0
positive regulation of cell death	4.1e-05	52	0	1
regulation of phosphorus metabolic process	9.2e-05	55	0	0
regulation of phosphate metabolic process	9.2e-05	55	0	0
regulation of phosphorylation	0.00015	53	0	0
positive regulation of multicellular organismal process	0.00017	35	0	0
anti-apoptosis	0.00036	31	0	0
response to organic substance	0.00052	70	1	0
negative regulation of apoptosis	0.00054	43	24	1
lymphocyte activation	0.00057	30	0	0
negative regulation of programmed cell death	0.00079	43	1	0
regulation of transferase activity	0.0008	44	0	0
negative regulation of cell death	0.00085	43	0	0
regulation of protein amino acid phosphorylation	0.0013	27	0	0
positive regulation of cell activation	0.0013	21	1	1
regulation of cytokine biosynthetic process	0.0014	17	0	0
positive regulation of cell communication	0.0015	40	0	0
positive regulation of response to stimulus	0.0023	32	0	0
regulation of protein kinase cascade	0.0025	33	0	0
response to cytokine stimulus	0.0035	17	1	1
regulation of cellular protein metabolic process	0.0035	50	0	0
enzyme linked receptor protein signaling pathway	0.0039	40	0	0
apoptosis	0.0039	59	29	0
regulation of MAPKKK cascade	0.0043	20	2	0
positive regulation of phosphorus metabolic process	0.0049	19	0	0
positive regulation of phosphate metabolic process	0.0049	19	0	0
positive regulation of signal transduction	0.0053	36	0	0
regulation of cell activation	0.0053	26	0	0
positive regulation of biosynthetic process	0.0053	65	0	0
programmed cell death	0.0062	59	0	0
positive regulation of cellular biosynthetic process	0.0067	64	0	0
immune effector process	0.0075	22	0	0
positive regulation of immune response	0.0077	23	0	0
positive regulation of transferase activity	0.0094	31	0	0
positive regulation of macromolecule metabolic process	0.0094	75	0	0
regulation of lymphocyte activation	0.011	23	0	0
regulation of kinase activity	0.011	40	0	0
response to molecule of bacterial origin	0.011	17	1	0
positive regulation of leukocyte activation	0.011	19	0	0
regulation of protein kinase activity	0.012	39	1	0
positive regulation of kinase activity	0.012	30	0	0
positive regulation of phosphorylation	0.013	18	3	0
positive regulation of lymphocyte activation	0.013	18	0	0
regulation of stress-activated protein kinase signaling pathway	0.015	15	0	0
response to nutrient	0.015	22	2	0
regulation of hydrolase activity	0.017	38	0	0
positive regulation of protein amino acid phosphorylation	0.018	17	1	1
positive regulation of protein kinase activity	0.018	29	1	0
positive regulation of cytokine production	0.02	17	1	0
regulation of leukocyte activation	0.022	24	0	0
regulation of smooth muscle cell proliferation	0.027	12	0	0
cell death	0.031	64	10	0
response to bacterium	0.031	26	0	0
regulation of lymphocyte proliferation	0.031	16	0	0
negative regulation of molecular function	0.033	37	0	0
negative regulation of cell proliferation	0.034	39	14	1
regulation of leukocyte proliferation	0.036	16	0	0
regulation of mononuclear cell proliferation	0.036	16	0	0
regulation of JNK cascade	0.037	14	0	0
T cell activation	0.038	20	2	0

death	0.038	64	0	0
positive regulation of cytokine biosynthetic process	0.041	12	0	0
positive regulation of specific transcription from RNA polymerase II promoter	0.044	13	0	0
positive regulation of macromolecule biosynthetic process	0.048	59	1	0
cell proliferation	0.049	44	19	0
sum		3392	180	12

64 CD14+ monocytes vs. CD8+ T cells (down-regulated)

GO term	FDR	Counts at both methods	Counts at RECLU	Counts at original Paraclu
lymphocyte activation	0.00022	20	0	0
T cell activation	0.0016	15	0	0
leukocyte activation	0.0044	20	0	0
lymphocyte differentiation	0.006	13	0	0
leukocyte differentiation	0.014	14	0	0
hemopoiesis	0.045	18	1	0
cell activation	0.05	20	0	0
sum		120	1	0

65 CD14+ monocytes vs. Macrophage - monocyte derived (up-regulated)

GO term	FDR	Counts at both methods	Counts at RECLU	Counts at original Paraclu
leukocyte activation	2e-11	48	0	0
lymphocyte activation	1.3e-10	42	0	0
cell activation	2.5e-10	51	0	0
regulation of apoptosis	4e-10	96	5	1
regulation of programmed cell death	7.2e-10	96	0	0
regulation of cell death	9e-10	96	0	0
T cell activation	3.6e-08	30	2	0
immune system development	4.4e-08	46	0	0
hemopoiesis	4.5e-08	42	1	0
hemopoietic or lymphoid organ development	7.7e-08	44	0	0
leukocyte differentiation	1e-07	30	0	0
intracellular signaling cascade	1.1e-07	123	0	0
lymphocyte differentiation	2.6e-07	26	0	0
regulation of phosphorylation	5.2e-07	61	1	0
regulation of phosphate metabolic process	9.5e-07	62	0	0
regulation of phosphorus metabolic process	9.5e-07	62	1	0
immune response	1.4e-06	78	13	0
regulation of kinase activity	8.2e-06	49	0	0
positive regulation of immune system process	1e-05	38	0	0
T cell differentiation	1.1e-05	19	3	0
regulation of transferase activity	1.1e-05	50	0	0
positive regulation of cell proliferation	1.9e-05	53	24	1
positive regulation of apoptosis	2.7e-05	54	9	1
positive regulation of programmed cell death	3.5e-05	54	0	0
positive regulation of cell death	4e-05	54	1	1
positive regulation of cell communication	4.3e-05	45	0	0
protein kinase cascade	7.2e-05	48	6	0
regulation of protein kinase cascade	0.00011	37	0	0
positive regulation of signal transduction	0.00013	41	0	0
positive regulation of kinase activity	0.00017	35	0	0
regulation of protein kinase activity	0.00018	45	0	0
regulation of cell proliferation	0.00021	79	5	0
positive regulation of transferase activity	0.00044	35	1	0
regulation of cytokine production	0.00083	29	0	0
negative regulation of apoptosis	0.00096	44	30	0
regulation of smooth muscle cell proliferation	0.0011	14	0	0
protein amino acid phosphorylation	0.0011	68	3	0
anti-apoptosis	0.0012	31	0	0
negative regulation of programmed cell death	0.0014	44	0	0
negative regulation of cell death	0.0015	44	0	0
regulation of cellular protein metabolic process	0.0016	53	0	0
response to protein stimulus	0.0018	21	0	0
regulation of T cell activation	0.0019	22	0	0
response to wounding	0.002	57	0	1
apoptosis	0.0027	62	38	3
negative regulation of macromolecule metabolic process	0.004	71	0	0
programmed cell death	0.0043	62	0	0
death	0.0049	70	0	0
positive regulation of smooth muscle cell proliferation	0.0058	11	3	0
enzyme linked receptor protein signaling pathway	0.0062	41	0	0
positive regulation of protein kinase activity	0.0066	31	0	0
cell death	0.0075	69	13	0
positive regulation of response to stimulus	0.0075	32	0	0
regulation of lymphocyte activation	0.008	24	0	0
regulation of MAPKKK cascade	0.0098	20	0	0
positive regulation of molecular function	0.01	59	0	0
regulation of transcription from RNA polymerase II promoter	0.011	69	7	0
positive regulation of macromolecule metabolic process	0.012	78	0	0
regulation of protein amino acid phosphorylation	0.012	26	1	0
regulation of lymphocyte proliferation	0.014	17	0	0
response to organic substance	0.016	68	0	0
regulation of mononuclear cell proliferation	0.017	17	0	0
regulation of leukocyte proliferation	0.017	17	0	0
regulation of leukocyte activation	0.018	25	0	0
positive regulation of protein kinase cascade	0.02	25	0	0
B cell activation	0.02	16	0	0
negative regulation of molecular function	0.021	39	0	0
regulation of T cell differentiation	0.024	13	0	0
positive regulation of leukocyte activation	0.025	19	0	0
regulation of I-kappaB kinase/NF-kappaB cascade	0.029	19	1	0
positive regulation of biosynthetic process	0.034	65	0	0
regulation of transcription	0.039	186	0	0
phosphorylation	0.041	72	1	0
positive regulation of cellular biosynthetic process	0.041	64	0	0
cell proliferation	0.041	46	17	0
regulation of cell activation	0.044	25	0	0
regulation of binding	0.045	23	0	0
negative regulation of macromolecule biosynthetic process	0.047	54	0	0
positive regulation of cell activation	0.048	19	1	1
regulation of cellular component size	0.049	33	0	0
sum		3783	187	9

66 CD14+ monocytes vs. Macrophage - monocyte derived (down-regulated)

GO term	FDR	Counts at both methods	Counts at RECLU	Counts at original Paraclu
oxidation reduction	1.5e-08	95	2	0
cell division	5e-06	52	10	2
mitosis	2e-05	42	10	5
nuclear division	2e-05	42	0	0
organelle fission	2.2e-05	43	0	0
M phase of mitotic cell cycle	3.4e-05	42	0	0
coenzyme metabolic process	0.00018	32	0	0
cofactor metabolic process	0.0002	37	0	0
M phase	0.0002	52	0	0
cell cycle	0.0046	91	9	0
DNA replication initiation	0.0091	9	0	0
DNA packaging	0.013	24	1	0
cellular response to stress	0.014	70	0	0
chromosome segregation	0.022	19	1	3
electron transport chain	0.028	23	1	0
oxidative phosphorylation	0.029	21	3	0
sum		694	37	10

67 CD14+ monocytes vs. Neutrophils (up-regulated)

GO term	FDR	Counts at both methods	Counts at RECLU	Counts at original Paraclu
translation	2.4e-21	86	18	0
translational elongation	1.2e-12	37	7	0
ribosome biogenesis	2.4e-08	35	3	0
ribonucleoprotein complex biogenesis	8.1e-07	41	0	0
ncRNA metabolic process	5.2e-06	46	2	0
rRNA metabolic process	0.00024	25	0	0
ncRNA processing	0.0003	37	0	0
rRNA processing	0.00044	24	15	0
regulation of apoptosis	0.00075	101	17	0
regulation of programmed cell death	0.0012	101	0	0
regulation of cell death	0.0015	101	1	0
positive regulation of immune system process	0.0026	41	1	0
leukocyte activation	0.0041	41	0	0
lymphocyte activation	0.0041	36	0	0
apoptosis	0.012	77	44	2
T cell activation	0.013	26	4	0
programmed cell death	0.02	77	1	0
positive regulation of apoptosis	0.021	59	19	0
anti-apoptosis	0.024	35	0	0
positive regulation of programmed cell death	0.026	59	0	0
leukocyte differentiation	0.027	26	0	0
positive regulation of cell death	0.031	59	0	1
hemopoiesis	0.033	38	5	0
T cell differentiation	0.036	17	3	0
positive regulation of innate immune response	0.045	14	0	0
sum		1239	140	3

68 CD14+ monocytes vs. Neutrophils (down-regulated)

GO term	FDR	Counts at both methods	Counts at RECLU	Counts at original Paraclu
immune response	5.5e-10	70	3	3
defense response	2.1e-08	62	1	0
inflammatory response	6.2e-07	40	9	3
response to wounding	1.1e-06	53	0	1
actin cytoskeleton organization	0.0013	27	2	0
actin filament-based process	0.0044	27	0	0
intracellular signaling cascade	0.0057	82	0	0
sum		361	15	7

69 CD19+ B cells vs. CD34 cells differentiated to erythrocyte lineage (up-regulated)

GO term	FDR	Counts at both methods	Counts at RECLU	Counts at original Paraclu
immune response	2.1e-14	104	6	1
leukocyte activation	9.4e-12	52	0	0
lymphocyte activation	5.5e-10	44	0	0
cell activation	2.9e-09	53	0	0
regulation of T cell activation	2.4e-08	31	0	0
regulation of lymphocyte activation	2.9e-08	35	0	0
positive regulation of lymphocyte activation	3.2e-08	28	0	0
T cell activation	3.4e-08	32	2	0
positive regulation of T cell activation	1.5e-07	24	1	0
regulation of alpha-beta T cell activation	1.6e-07	17	0	0
positive regulation of leukocyte activation	2.9e-07	28	0	0
regulation of leukocyte activation	7.9e-07	35	0	0
regulation of cell activation	8.6e-07	36	0	0
positive regulation of cell activation	9.1e-07	28	0	0
apoptosis	2.8e-06	77	23	1
positive regulation of immune system process	3.3e-06	42	0	0
programmed cell death	5.4e-06	77	0	0
regulation of lymphocyte differentiation	7.2e-06	20	0	0
regulation of T cell differentiation	8.6e-06	18	0	0
positive regulation of lymphocyte differentiation	1.3e-05	15	0	0
positive regulation of alpha-beta T cell activation	2.2e-05	13	0	0
hemopoiesis	2.7e-05	40	3	1
positive regulation of T cell differentiation	3.6e-05	14	1	0
leukocyte differentiation	4.3e-05	28	0	0
death	4.5e-05	84	0	0
hemopoietic or lymphoid organ development	4.7e-05	42	0	0
positive regulation of apoptosis	6.2e-05	58	12	0
intracellular signaling cascade	6.2e-05	126	0	0
cell death	7e-05	83	11	0
positive regulation of programmed cell death	7.9e-05	58	0	0
immune system development	9e-05	43	0	0
positive regulation of cell death	9.4e-05	58	0	0
lymphocyte differentiation	9.8e-05	24	0	0
regulation of apoptosis	0.00014	89	0	0
regulation of programmed cell death	0.00023	89	0	0
regulation of cell death	0.00026	89	0	0
phosphorus metabolic process	0.00039	101	0	0
phosphate metabolic process	0.00039	101	1	0
T cell differentiation	0.00048	18	3	0
regulation of lymphocyte proliferation	0.00093	20	0	0
immune effector process	0.0011	26	0	0
regulation of mononuclear cell proliferation	0.0011	20	0	0
regulation of leukocyte proliferation	0.0011	20	0	0
positive regulation of lymphocyte proliferation	0.0013	16	0	0
defense response	0.0016	70	1	0
positive regulation of leukocyte proliferation	0.0017	16	0	0
positive regulation of mononuclear cell proliferation	0.0017	16	0	0
regulation of alpha-beta T cell differentiation	0.0019	11	0	0
protein amino acid phosphorylation	0.002	74	5	0
positive regulation of alpha-beta T cell differentiation	0.0021	10	1	0
leukocyte mediated immunity	0.0073	19	0	0
regulation of alpha-beta T cell proliferation	0.0075	8	0	0
regulation of cytokine production	0.011	29	0	0
antigen processing and presentation	0.018	18	1	0
positive regulation of B cell activation	0.035	11	1	0
protein kinase cascade	0.04	45	6	0
inflammatory response	0.042	41	15	0
positive regulation of cytokine biosynthetic process	0.045	13	0	0
sum		2437	93	3

70 CD19+ B cells vs. CD34 cells differentiated to erythrocyte lineage (down-regulated)

GO term	FDR	Counts at both methods	Counts at RECLU	Counts at original Paraclu
DNA packaging	2.6e-20	44	0	0
protein-DNA complex assembly	4.6e-20	39	0	0
nucleosome assembly	2.3e-19	37	3	0
chromatin assembly	9.7e-19	37	0	0
nucleosome organization	1.3e-18	38	0	0
cell cycle	9.1e-16	112	17	0
chromatin assembly or disassembly	4.4e-15	40	0	0
chromosome organization	9.4e-15	82	1	0
mitotic cell cycle	8.2e-12	65	12	0
cell cycle process	3.5e-11	83	1	0
cell cycle phase	5.4e-11	68	0	0
organelle fission	6.3e-11	48	0	0
M phase	7.2e-11	59	0	0
mitosis	2.5e-10	46	13	0
nuclear division	2.5e-10	46	0	0
M phase of mitotic cell cycle	5e-10	46	0	0
chromatin organization	9.2e-10	62	0	0
cellular macromolecular complex subunit organization	8.6e-08	56	0	0
cellular macromolecular complex assembly	1e-07	52	0	0
cell division	2.2e-07	49	14	1
chromosome segregation	0.00031	20	3	0
macromolecular complex subunit organization	0.00049	77	0	0
macromolecular complex assembly	0.00067	73	0	0
DNA replication initiation	0.0022	9	1	0
DNA metabolic process	0.0072	57	0	0
negative regulation of organelle organization	0.0086	18	0	0
DNA replication	0.012	29	11	0
microtubule-based process	0.028	34	1	0
sum		1426	77	1

71 CD19+ B cells vs. CD4+CD25-CD45RA+ naive conventional T cell (up-regulated)

GO term	FDR	Counts at both methods	Counts at RECLU	Counts at original Paraclu
response to organic substance	0.014	25	2	0
RNA stabilization	0.046	5	0	0
mRNA stabilization	0.046	5	3	0
sum		35	5	0

72 CD19+ B cells vs. CD4+CD25-CD45RA+ naive conventional T cell (down-regulated)

GO term	FDR	Counts at both methods	Counts at RECLU	Counts at original Paraclu
regulation of T cell activation	1.7e-06	19	0	1
leukocyte differentiation	1.1e-05	19	0	0
immune response	1.2e-05	46	2	19
lymphocyte differentiation	1.3e-05	17	0	0
lymphocyte activation	1.4e-05	23	0	1
positive regulation of immune system process	1.9e-05	25	1	0
leukocyte activation	2.6e-05	25	0	0
T cell activation	3.8e-05	18	0	3
regulation of lymphocyte activation	7.7e-05	19	0	0
T cell differentiation	0.00014	13	0	3
regulation of cell activation	0.0002	20	0	0
hemopoiesis	0.00032	23	0	7
regulation of leukocyte activation	0.00045	19	0	0
cell activation	0.00066	25	0	0
positive regulation of T cell activation	0.00083	13	0	0
protein amino acid phosphorylation	0.00083	41	4	3
regulation of lymphocyte differentiation	0.00087	12	0	0
regulation of T cell differentiation	0.00094	11	0	1
hemopoietic or lymphoid organ development	0.0017	23	0	0
positive regulation of T cell differentiation	0.0018	9	0	0
regulation of apoptosis	0.0026	45	1	7
phosphate metabolic process	0.003	51	0	1
phosphorus metabolic process	0.003	51	0	0
regulation of programmed cell death	0.0034	45	0	0
regulation of cell death	0.0038	45	0	0
positive regulation of lymphocyte differentiation	0.0038	9	0	0
immune system development	0.0045	23	0	0
positive regulation of leukocyte activation	0.0053	14	0	0
positive regulation of cell activation	0.009	14	0	0
positive regulation of lymphocyte activation	0.012	13	0	0
phosphorylation	0.013	43	1	0
sum		773	9	46

73 CD19+ B cells vs. CD4+ T cells (up-regulated)

GO term	FDR	Counts at both methods	Counts at RECLU	Counts at original Paraclu
immune response	1.6e-12	55	4	0
positive regulation of immune system process	2.4e-10	30	0	0
leukocyte activation	2.4e-09	29	0	0
inflammatory response	4.7e-09	33	10	0
cell activation	5.1e-09	31	0	0
defense response	5.4e-09	46	1	0
positive regulation of cell activation	1.1e-08	20	0	0
regulation of cell activation	1.9e-08	24	0	0
regulation of T cell activation	2.9e-08	20	0	0
regulation of lymphocyte activation	3.4e-08	22	0	0
lymphocyte activation	4.2e-08	25	0	0
positive regulation of lymphocyte activation	9.7e-08	18	0	0
response to wounding	1.5e-07	40	1	0
positive regulation of T cell activation	2.3e-07	16	1	0
regulation of leukocyte activation	3.1e-07	22	0	0
positive regulation of leukocyte activation	4.2e-07	18	0	0
positive regulation of immune response	1.3e-06	20	1	0
regulation of T cell differentiation	2e-06	13	1	0
regulation of lymphocyte differentiation	2.3e-06	14	0	0
regulation of alpha-beta T cell activation	8.5e-06	11	0	0
positive regulation of response to stimulus	3.8e-05	23	0	0
positive regulation of T cell differentiation	4.5e-05	10	0	0
positive regulation of lymphocyte proliferation	6.1e-05	12	0	0
positive regulation of leukocyte proliferation	7.4e-05	12	0	0
positive regulation of mononuclear cell proliferation	7.4e-05	12	0	0
regulation of lymphocyte proliferation	7.6e-05	14	0	0
regulation of mononuclear cell proliferation	8.8e-05	14	0	0
regulation of leukocyte proliferation	8.8e-05	14	0	0
positive regulation of lymphocyte differentiation	0.00011	10	0	0
T cell activation	0.0003	16	1	0
immune effector process	0.00068	16	0	0
positive regulation of immune effector process	0.0009	10	0	0
regulation of cytokine production	0.0014	18	0	0
positive regulation of cytokine biosynthetic process	0.002	10	0	0
positive regulation of alpha-beta T cell activation	0.0023	8	0	0
antigen processing and presentation	0.0047	12	1	0
lymphocyte differentiation	0.0066	13	0	0
leukocyte mediated immunity	0.0067	12	0	0
regulation of cytokine biosynthetic process	0.011	11	0	0
regulation of alpha-beta T cell proliferation	0.011	6	0	0
antigen processing and presentation of peptide or polysaccharide antigen via MHC class II	0.013	8	0	0
B cell activation	0.014	11	0	0
leukocyte differentiation	0.016	14	0	0
regulation of T cell proliferation	0.018	10	0	0
humoral immune response	0.02	11	0	1
regulation of interleukin-6 production	0.024	8	1	0
positive regulation of T cell mediated immunity	0.026	6	0	0
innate immune response	0.028	14	14	1
regulation of alpha-beta T cell differentiation	0.028	7	0	0
regulation of B cell activation	0.031	9	0	0
regulation of immune effector process	0.032	12	0	0
immune system development	0.032	20	0	0
positive regulation of T cell proliferation	0.043	8	2	0
positive regulation of cell proliferation	0.047	25	7	0
lymphocyte mediated immunity	0.049	10	0	0
sum		933	45	2

74 CD19+ B cells vs. CD4+ T cells (down-regulated)

GO term	FDR	Counts at both methods	Counts at RECLU	Counts at original Paraclu
lymphocyte activation	6.4e-05	20	0	0
leukocyte activation	0.00031	21	0	0
lymphocyte differentiation	0.00038	14	0	0
immune response	0.00065	37	8	1
T cell activation	0.00065	15	1	0
leukocyte differentiation	0.0011	15	0	0
positive regulation of immune system process	0.0011	20	0	0
cell activation	0.0011	22	0	0
regulation of T cell activation	0.0017	14	1	0
regulation of lymphocyte activation	0.0046	15	0	0
T cell differentiation	0.013	10	1	1
hemopoiesis	0.017	18	3	1
protein amino acid phosphorylation	0.017	33	3	0
regulation of leukocyte activation	0.018	15	0	0
regulation of apoptosis	0.022	37	5	0
regulation of programmed cell death	0.027	37	0	0
regulation of cell death	0.029	37	0	0
regulation of cell activation	0.032	15	1	0
sum		395	23	3

75 CD19+ B cells vs. CD8+ T cells (up-regulated)

GO term	FDR	Counts at both methods	Counts at RECLU	Counts at original Paraclu
immune response	2e-09	48	4	0
positive regulation of immune system process	1.4e-08	27	0	0
inflammatory response	2.4e-08	31	11	0
response to wounding	8.4e-08	39	0	0
leukocyte activation	1.2e-07	26	0	0
cell activation	8.9e-07	27	0	0
defense response	1.7e-06	40	1	0
positive regulation of cell activation	2.8e-06	17	0	0
lymphocyte activation	2.8e-06	22	0	0
positive regulation of lymphocyte activation	3.4e-06	16	0	0
regulation of lymphocyte activation	4.5e-06	19	0	0
regulation of cell activation	1.1e-05	20	0	0
positive regulation of leukocyte activation	1.2e-05	16	0	0
regulation of leukocyte activation	2.8e-05	19	0	0
regulation of T cell activation	4.7e-05	16	0	0
positive regulation of T cell activation	0.00011	13	1	0
regulation of lymphocyte differentiation	0.00014	12	0	0
regulation of T cell differentiation	0.00017	11	1	0
positive regulation of T cell differentiation	0.00046	9	0	0
positive regulation of lymphocyte differentiation	0.00097	9	0	0
positive regulation of cytokine biosynthetic process	0.0011	10	0	0
regulation of alpha-beta T cell activation	0.0012	9	0	0
immune effector process	0.0018	15	0	0
positive regulation of immune response	0.0046	15	1	0
T cell activation	0.005	14	1	0
regulation of cytokine biosynthetic process	0.0063	11	0	0
B cell activation	0.0081	11	0	0
antigen processing and presentation of peptide or polysaccharide antigen via MHC class II	0.0087	8	0	0
humoral immune response	0.011	11	1	1
regulation of cytokine production	0.014	16	0	0
antigen processing and presentation	0.018	11	1	0
regulation of alpha-beta T cell differentiation	0.02	7	0	0
lymphocyte differentiation	0.021	12	0	0
leukocyte mediated immunity	0.025	11	0	0
positive regulation of alpha-beta T cell activation	0.025	7	0	0
positive regulation of lymphocyte proliferation	0.035	9	0	0
positive regulation of mononuclear cell proliferation	0.04	9	0	0
positive regulation of leukocyte proliferation	0.04	9	0	0
leukocyte differentiation	0.041	13	0	0
sum		645	22	1

76 CD19+ B cells vs. CD8+ T cells (down-regulated)

GO term	FDR	Counts at both methods	Counts at RECLU	Counts at original Paraclu
lymphocyte activation	0.00016	22	0	0
lymphocyte differentiation	0.00017	16	0	0
immune response	0.00037	44	7	0
T cell activation	0.00044	17	0	0
leukocyte differentiation	0.00075	17	0	0
leukocyte activation	0.0011	23	0	0
cell activation	0.0015	25	0	0
T cell differentiation	0.0019	12	2	0
intracellular signaling cascade	0.024	60	0	0
hemopoiesis	0.036	20	2	0
positive regulation of immune system process	0.04	20	0	0
sum		276	11	0

77 CD19+ B cells vs. Macrophage - monocyte derived (up-regulated)

GO term	FDR	Counts at both methods	Counts at RECLU	Counts at original Paraclu
transcription	1.9e-07	151	0	0
regulation of transcription	4e-07	176	0	0
chromatin organization	0.00011	42	0	0
lymphocyte activation	0.0009	27	0	0
regulation of T cell activation	0.0011	20	0	0
leukocyte activation	0.0013	30	0	0
translational elongation	0.0026	18	2	3
regulation of lymphocyte activation	0.0029	22	0	0
T cell activation	0.0036	20	1	0
positive regulation of lymphocyte activation	0.007	17	0	0
positive regulation of T cell activation	0.0074	15	3	0
chromosome organization	0.012	44	0	0
regulation of cell activation	0.013	23	0	0
cell activation	0.014	31	0	0
chromatin modification	0.015	30	3	0
lymphocyte differentiation	0.016	17	0	0
hemopoietic or lymphoid organ development	0.016	29	0	0
immune system development	0.018	30	0	0
regulation of leukocyte activation	0.019	22	0	0
positive regulation of leukocyte activation	0.023	17	0	0
regulation of lymphocyte differentiation	0.025	13	0	0
leukocyte differentiation	0.025	19	0	0
regulation of alpha-beta T cell activation	0.03	10	0	0
positive regulation of cell activation	0.041	17	0	0
sum		840	9	3

78 CD19+ B cells vs. Macrophage - monocyte derived (down-regulated)

GO term	FDR	Counts at both methods	Counts at RECLU	Counts at original Paraclu
oxidation reduction	5.2e-10	100	1	0
coenzyme metabolic process	0.00027	32	0	0
cofactor metabolic process	0.00033	37	0	0
response to wounding	0.00063	72	2	0
inflammatory response	0.0015	50	11	1
immune response	0.019	82	14	0
defense response	0.02	75	2	0
carboxylic acid biosynthetic process	0.034	28	0	0
organic acid biosynthetic process	0.034	28	0	0
sulfur metabolic process	0.044	23	0	0
sum		527	30	1

79 CD19+ B cells vs. Neutrophils (up-regulated)

GO term	FDR	Counts at both methods	Counts at RECLU	Counts at original Paraclu
translation	1.1e-29	100	14	0
translational elongation	2.3e-18	44	1	0
ncRNA metabolic process	6.2e-18	67	3	0
ribonucleoprotein complex biogenesis	1.5e-14	54	0	0
ribosome biogenesis	4e-13	42	4	0
RNA processing	1.6e-12	101	4	0
ncRNA processing	5.2e-11	50	0	0
rRNA metabolic process	1.3e-09	33	0	0
rRNA processing	1.3e-08	31	4	0
nucleosome organization	5.5e-07	29	0	0
nucleosome assembly	1.2e-06	27	9	0
chromatin assembly	2.8e-06	27	0	0
cellular macromolecular complex subunit organization	4.6e-06	63	0	0
cellular macromolecular complex assembly	6.3e-06	58	0	0
macromolecular complex assembly	6.8e-06	97	0	0
protein-DNA complex assembly	8.2e-06	27	0	0
tRNA metabolic process	1.1e-05	31	0	0
macromolecular complex subunit organization	2.4e-05	100	0	0
chromatin organization	0.0001	62	0	0
chromatin assembly or disassembly	0.00026	30	0	0
DNA packaging	0.002	27	0	0
tRNA aminoacylation	0.0024	16	0	0
tRNA aminoacylation for protein translation	0.0024	16	5	0
amino acid activation	0.0024	16	0	0
chromosome organization	0.004	69	2	0
mitochondrion organization	0.016	28	3	0
sum		1245	49	0

80 CD19+ B cells vs. Neutrophils (down-regulated)

GO term	FDR	Counts at both methods	Counts at RECLU	Counts at original Paraclu
immune response	2.7e-11	78	5	2
response to wounding	1.6e-09	63	0	0
defense response	5e-09	68	1	0
inflammatory response	3.3e-08	45	5	6
intracellular signaling cascade	0.00036	94	0	0
immune effector process	0.00058	22	0	0
response to bacterium	0.0019	26	0	0
actin cytoskeleton organization	0.0035	28	5	0
response to molecule of bacterial origin	0.0072	16	0	0
leukocyte mediated immunity	0.0072	16	0	0
response to lipopolysaccharide	0.0091	15	3	2
protein kinase cascade	0.01	37	1	0
taxis	0.011	22	0	0
chemotaxis	0.011	22	1	1
actin filament-based process	0.012	28	0	0
regulation of apoptosis	0.014	63	1	1
innate immune response	0.015	20	15	7
regulation of programmed cell death	0.019	63	0	0
regulation of cell death	0.021	63	0	0
protein amino acid phosphorylation	0.028	54	1	0
apoptosis	0.033	50	14	4
myeloid leukocyte activation	0.041	11	0	0
programmed cell death	0.049	50	0	0
sum		954	52	23

81 CD34 cells differentiated to erythrocyte lineage vs. CD4+CD25-CD45RA+ naive conventional T cell (up-regulated)

GO term	FDR	Counts at both methods	Counts at RECLU	Counts at original Paraclu
protein-DNA complex assembly	2.3e-14	33	0	0
DNA packaging	3.1e-14	37	0	0
nucleosome organization	4.8e-14	33	0	0
nucleosome assembly	2e-13	31	8	0
chromatin assembly	4e-13	31	0	0
cell cycle	8.6e-11	99	16	1
chromosome organization	9.4e-10	71	1	0
chromatin assembly or disassembly	1.1e-09	33	0	0
cell cycle phase	3.9e-08	61	0	0
mitotic cell cycle	1.1e-07	56	12	0
cell cycle process	1.8e-07	73	0	0
M phase	3.7e-07	51	0	0
organelle fission	3.7e-07	41	0	0
nuclear division	1.5e-06	39	0	0
mitosis	1.5e-06	39	13	0
M phase of mitotic cell cycle	2.5e-06	39	0	0
chromatin organization	4.4e-05	51	0	0
cell division	7.4e-05	43	16	2
cellular macromolecular complex subunit organization	0.00035	47	0	0
chromosome segregation	0.00098	19	5	0
DNA metabolic process	0.0012	58	0	0
cellular macromolecular complex assembly	0.0016	42	0	0
regulation of cell cycle	0.0045	42	3	0
negative regulation of organelle organization	0.0056	18	0	0
DNA replication	0.0068	29	8	0
negative regulation of cellular component organization	0.01	24	0	0
cellular response to stress	0.039	58	1	0
regulation of protein complex disassembly	0.041	13	0	0
negative regulation of mitosis	0.043	7	0	0
negative regulation of nuclear division	0.043	7	0	0
carboxylic acid biosynthetic process	0.044	24	0	0
organic acid biosynthetic process	0.044	24	0	0
sum		1273	83	3

82 CD34 cells differentiated to erythrocyte lineage vs. CD4+CD25-CD45RA+ naive conventional T cell (down-regulated)

GO term	FDR	Counts at both methods	Counts at RECLU	Counts at original Paraclu
leukocyte activation	8.4e-07	44	0	0
lymphocyte activation	1.1e-06	39	0	0
regulation of T cell activation	4.7e-06	28	0	0
T cell activation	5.9e-06	29	1	0
protein amino acid phosphorylation	1.1e-05	82	3	0
phosphorus metabolic process	3.8e-05	106	0	0
phosphate metabolic process	3.8e-05	106	0	0
immune response	5.1e-05	82	3	1
leukocyte differentiation	6.2e-05	28	0	0
regulation of lymphocyte activation	6.3e-05	30	0	0
regulation of T cell differentiation	8.4e-05	17	0	0
lymphocyte differentiation	0.00014	24	0	0
hemopoiesis	0.00014	39	0	2
cell activation	0.00016	44	1	0
regulation of alpha-beta T cell activation	0.00024	14	0	0
regulation of lymphocyte differentiation	0.00038	18	0	0
T cell differentiation	0.00062	18	0	0
regulation of cell activation	0.00081	31	0	0
regulation of leukocyte activation	0.00084	30	0	0
hemopoietic or lymphoid organ development	0.0017	39	0	0
phosphorylation	0.0018	86	0	0
positive regulation of T cell differentiation	0.0039	12	0	0
immune system development	0.0074	39	0	0
regulation of apoptosis	0.0079	84	0	0
positive regulation of immune system process	0.011	35	0	0
positive regulation of lymphocyte differentiation	0.011	12	0	0
regulation of programmed cell death	0.011	84	0	0
intracellular signaling cascade	0.013	118	0	0
regulation of cell death	0.013	84	0	0
regulation of alpha-beta T cell differentiation	0.022	10	0	0
positive regulation of alpha-beta T cell differentiation	0.027	9	0	0
positive regulation of T cell activation	0.029	17	0	0
positive regulation of alpha-beta T cell activation	0.031	10	0	0
sum		1448	8	3

83 CD34 cells differentiated to erythrocyte lineage vs. CD4+ T cells (up-regulated)

GO term	FDR	Counts at both methods	Counts at RECLU	Counts at original Paraclu
protein-DNA complex assembly	8.6e-15	36	0	0
chromosome organization	2.5e-14	89	1	0
DNA packaging	3.5e-14	40	0	0
nucleosome assembly	3.6e-14	34	4	0
chromatin assembly	2e-13	34	0	0
nucleosome organization	2e-13	35	0	0
chromatin assembly or disassembly	2e-10	37	0	0
cell cycle	2.5e-10	111	12	2
M phase	1.4e-08	60	0	0
DNA metabolic process	1.9e-08	79	0	0
cell cycle phase	2.3e-08	69	0	0
cell cycle process	5e-08	84	1	0
organelle fission	6e-08	47	0	0
mitotic cell cycle	8.3e-08	63	7	0
nuclear division	1.9e-07	45	0	0
mitosis	1.9e-07	45	8	0
chromatin organization	2.1e-07	63	0	0
M phase of mitotic cell cycle	3.5e-07	45	0	0
cellular macromolecular complex assembly	9.3e-07	55	0	0
cellular macromolecular complex subunit organization	3.4e-06	58	0	0
chromosome segregation	3e-05	23	3	0
macromolecular complex assembly	3e-05	86	0	0
cell division	3.5e-05	49	8	3
macromolecular complex subunit organization	7.1e-05	89	0	0
DNA replication	0.00043	35	7	0
cellular response to stress	0.00099	72	1	0
response to DNA damage stimulus	0.0034	52	3	0
negative regulation of organelle organization	0.004	20	0	0
nitrogen compound biosynthetic process	0.0043	47	0	0
heterocycle biosynthetic process	0.014	16	0	0
cofactor metabolic process	0.019	32	0	0
DNA repair	0.022	41	12	0
negative regulation of cellular component organization	0.022	26	0	0
sum		1717	67	5

84 CD34 cells differentiated to erythrocyte lineage vs. CD4+ T cells (down-regulated)

GO term	FDR	Counts at both methods	Counts at RECLU	Counts at original Paraclu
leukocyte activation	2.7e-09	52	1	0
lymphocyte activation	1.7e-08	45	0	0
regulation of T cell activation	3.4e-08	33	0	0
T cell activation	6e-08	34	0	0
immune response	9.6e-08	98	5	1
regulation of lymphocyte activation	1.4e-06	35	0	0
cell activation	1.8e-06	52	0	0
protein amino acid phosphorylation	1.9e-06	92	4	0
leukocyte differentiation	3.6e-06	32	0	0
lymphocyte differentiation	3.6e-06	28	0	0
hemopoiesis	1.8e-05	44	5	0
T cell differentiation	2.3e-05	21	0	1
phosphate metabolic process	3e-05	117	1	0
phosphorus metabolic process	3e-05	117	0	0
regulation of leukocyte activation	3.2e-05	35	0	0
intracellular signaling cascade	3.6e-05	142	0	0
regulation of cell activation	3.7e-05	36	0	0
regulation of T cell differentiation	7.5e-05	18	0	0
hemopoietic or lymphoid organ development	0.00012	45	0	0
regulation of alpha-beta T cell activation	0.00013	15	0	0
positive regulation of T cell differentiation	0.0002	14	0	0
positive regulation of T cell activation	0.00041	21	1	0
regulation of lymphocyte differentiation	0.00043	19	0	0
protein kinase cascade	0.00063	55	4	0
immune system development	0.00067	45	0	0
positive regulation of lymphocyte differentiation	0.0007	14	0	0
phosphorylation	0.0015	95	1	0
positive regulation of lymphocyte activation	0.0017	23	0	0
positive regulation of leukocyte activation	0.0022	24	0	0
positive regulation of immune system process	0.004	39	1	0
positive regulation of cell activation	0.0052	24	0	0
regulation of apoptosis	0.0063	93	10	0
positive regulation of alpha-beta T cell differentiation	0.0069	10	0	0
regulation of alpha-beta T cell differentiation	0.0072	11	0	0
regulation of programmed cell death	0.0096	93	0	0
positive regulation of alpha-beta T cell activation	0.011	11	0	0
regulation of cell death	0.011	93	1	0
positive T cell selection	0.016	7	0	0
apoptosis	0.017	73	25	2
programmed cell death	0.028	73	0	0
negative regulation of T cell activation	0.044	13	0	0
sum		1941	59	4

85 CD34 cells differentiated to erythrocyte lineage vs. CD8+ T cells (up-regulated)

GO term	FDR	Counts at both methods	Counts at RECLU	Counts at original Paraclu
protein-DNA complex assembly	4.6e-17	38	0	0
nucleosome organization	1.1e-16	38	0	0
nucleosome assembly	1.9e-16	36	2	0
DNA packaging	2.7e-16	42	0	0
chromatin assembly	7.3e-16	36	0	0
chromosome organization	8e-13	85	1	0
chromatin assembly or disassembly	2.6e-12	39	0	0
cell cycle	1.9e-11	112	16	2
M phase	1.7e-09	61	0	0
cellular macromolecular complex subunit organization	2e-09	64	0	0
cell cycle process	2.2e-09	86	0	0
cell cycle phase	2.7e-09	70	0	0
cellular macromolecular complex assembly	4e-09	59	0	0
mitotic cell cycle	1e-08	64	9	0
organelle fission	2.6e-08	47	0	0
DNA metabolic process	4e-08	77	0	0
nuclear division	8.6e-08	45	0	0
mitosis	8.6e-08	45	8	0
M phase of mitotic cell cycle	1.6e-07	45	0	0
chromatin organization	2.2e-07	62	0	0
macromolecular complex assembly	4.5e-06	87	0	0
macromolecular complex subunit organization	5.1e-06	91	0	0
cell division	5.8e-06	50	9	3
cellular response to stress	0.00019	73	2	0
chromosome segregation	0.0005	21	5	0
response to DNA damage stimulus	0.00073	53	6	0
DNA replication	0.00077	34	6	0
nitrogen compound biosynthetic process	0.0053	46	0	0
heterocycle biosynthetic process	0.01	16	0	0
negative regulation of organelle organization	0.012	19	0	0
DNA repair	0.012	41	11	0
negative regulation of cellular component organization	0.015	26	0	0
organic acid biosynthetic process	0.024	27	0	0
carboxylic acid biosynthetic process	0.024	27	0	0
DNA recombination	0.036	21	3	0
regulation of cell cycle	0.043	44	1	0
sum		1827	79	5

86 CD34 cells differentiated to erythrocyte lineage vs. CD8+ T cells (down-regulated)

GO term	FDR	Counts at both methods	Counts at RECLU	Counts at original Paraclu
leukocyte activation	4.6e-10	58	0	0
lymphocyte activation	1e-09	51	0	0
T cell activation	3.5e-08	37	2	0
lymphocyte differentiation	4e-08	33	0	0
immune response	8.1e-08	109	6	1
leukocyte differentiation	1.2e-07	37	0	0
cell activation	2.3e-07	59	0	0
protein amino acid phosphorylation	2.4e-07	105	3	2
hemopoiesis	7.9e-07	51	1	0
T cell differentiation	1.5e-06	24	1	0
regulation of T cell activation	1.6e-06	33	0	0
hemopoietic or lymphoid organ development	3.1e-06	53	0	0
regulation of T cell differentiation	1.6e-05	20	0	0
immune system development	2.6e-05	53	0	0
intracellular signaling cascade	3.1e-05	160	0	0
phosphate metabolic process	3.2e-05	131	1	0
phosphorus metabolic process	3.2e-05	131	0	0
regulation of I-kappaB kinase/NF-kappaB cascade	5.1e-05	29	0	0
regulation of lymphocyte differentiation	0.00015	21	0	0
regulation of lymphocyte activation	0.00021	34	0	0
positive regulation of protein kinase cascade	0.00043	36	0	0
phosphorylation	0.00063	108	0	0
regulation of protein kinase cascade	0.00084	46	0	0
positive regulation of T cell activation	0.001	22	3	0
positive regulation of T cell differentiation	0.0012	14	0	0
positive regulation of I-kappaB kinase/NF-kappaB cascade	0.0015	25	12	1
regulation of leukocyte activation	0.0033	34	0	0
positive regulation of lymphocyte differentiation	0.0039	14	0	0
regulation of cell activation	0.004	35	0	0
regulation of apoptosis	0.0044	105	8	0
regulation of cell death	0.0048	106	1	0
regulation of alpha-beta T cell activation	0.0056	14	0	0
protein kinase cascade	0.0061	58	1	1
regulation of programmed cell death	0.0071	105	0	0
induction of apoptosis	0.016	51	8	0
induction of programmed cell death	0.018	51	0	0
positive regulation of lymphocyte activation	0.02	23	0	0
positive regulation of immune system process	0.023	41	0	0
positive regulation of alpha-beta T cell differentiation	0.024	10	0	1
positive regulation of leukocyte activation	0.027	24	0	0
regulation of alpha-beta T cell differentiation	0.027	11	0	0
positive T cell selection	0.039	7	0	0
positive regulation of apoptosis	0.039	62	8	1
positive regulation of alpha-beta T cell activation	0.041	11	0	0
positive regulation of programmed cell death	0.048	62	1	0
sum		2304	56	7

87 CD34 cells differentiated to erythrocyte lineage vs. Macrophage - monocyte derived (up-regulated)

GO term	FDR	Counts at both methods	Counts at RECLU	Counts at original Paraclu
protein-DNA complex assembly	1.4e-23	39	0	0
DNA packaging	3.8e-23	43	0	0
nucleosome assembly	1e-22	37	3	0
chromatin assembly	4.5e-22	37	0	0
nucleosome organization	5.2e-22	38	0	0
chromosome organization	6.6e-21	82	0	0
chromatin assembly or disassembly	1.5e-17	39	0	0
chromatin organization	2.2e-14	62	0	0
cell cycle	5.9e-13	92	10	1
cell cycle phase	4.2e-10	58	0	0
cell cycle process	4e-09	68	0	0
M phase	1.6e-08	48	0	0
cellular macromolecular complex subunit organization	2.5e-08	50	0	0
cellular macromolecular complex assembly	6.1e-08	46	0	0
mitotic cell cycle	3e-07	49	12	0
organelle fission	3.1e-07	37	0	0
mitosis	1.6e-06	35	6	0
nuclear division	1.6e-06	35	0	0
M phase of mitotic cell cycle	2.6e-06	35	0	0
cell division	3.1e-05	39	9	1
chromosome segregation	5.4e-05	19	3	0
DNA replication	0.00051	28	7	0
macromolecular complex subunit organization	0.00074	65	0	0
macromolecular complex assembly	0.0017	61	0	0
DNA metabolic process	0.0056	49	0	0
DNA replication initiation	0.0071	8	0	0
sum		1199	50	2

88 CD34 cells differentiated to erythrocyte lineage vs. Macrophage - monocyte derived (down-regulated)

GO term	FDR	Counts at both methods	Counts at RECLU	Counts at original Paraclu
immune response	1e-17	131	5	2
inflammatory response	2.5e-08	65	8	1
intracellular signaling cascade	3.4e-08	168	0	0
defense response	5.5e-08	99	0	0
regulation of apoptosis	1.1e-07	119	3	0
regulation of programmed cell death	2.2e-07	119	0	0
regulation of cell death	2.7e-07	119	0	0
response to wounding	3.8e-07	87	0	0
actin cytoskeleton organization	2.1e-06	48	7	1
leukocyte activation	2.4e-06	50	0	0
actin filament-based process	6.2e-06	49	0	0
positive regulation of T cell differentiation	1e-05	16	1	0
immune effector process	3e-05	33	0	0
positive regulation of immune system process	3.5e-05	47	0	0
positive regulation of lymphocyte differentiation	4.6e-05	16	0	0
cell activation	0.00011	52	0	0
antigen processing and presentation of peptide antigen	0.00012	14	0	0
lymphocyte activation	0.00029	40	0	0
regulation of lymphocyte activation	0.00037	33	0	0
apoptosis	0.00073	85	21	1
regulation of cell activation	0.00074	36	1	0
small GTPase mediated signal transduction	0.00078	52	20	3
regulation of T cell activation	0.00083	28	0	0
positive regulation of apoptosis	0.00097	66	9	0
Ras protein signal transduction	0.0012	26	2	0
positive regulation of programmed cell death	0.0012	66	0	0
programmed cell death	0.0014	85	0	0
positive regulation of cell death	0.0015	66	0	0
positive regulation of alpha-beta T cell differentiation	0.0018	11	1	0
regulation of T cell differentiation	0.0025	17	1	0
negative regulation of apoptosis	0.0031	56	21	2
positive regulation of cell activation	0.0035	26	0	0
positive regulation of alpha-beta T cell activation	0.0041	12	0	0
regulation of alpha-beta T cell activation	0.0041	14	0	0
negative regulation of programmed cell death	0.0048	56	0	0
negative regulation of cell death	0.0052	56	0	0
regulation of leukocyte activation	0.0053	33	0	0
anti-apoptosis	0.0054	38	0	0
innate immune response	0.0079	29	28	1
cell death	0.009	93	10	0
positive regulation of CD4-positive, alpha beta T cell differentiation	0.0098	8	0	0
positive regulation of T cell activation	0.012	20	1	0
regulation of lymphocyte differentiation	0.012	18	0	0
death	0.012	93	0	0
positive regulation of lymphocyte activation	0.013	23	0	0
cell motion	0.015	67	4	0
induction of apoptosis	0.017	50	6	2
myeloid leukocyte activation	0.017	15	0	0
induction of programmed cell death	0.018	50	0	0
positive regulation of developmental process	0.021	45	0	0
regulation of alpha-beta T cell differentiation	0.022	11	0	0
protein kinase cascade	0.025	55	4	0
T cell activation	0.038	26	3	1
antigen processing and presentation	0.046	20	2	0
sum		2757	158	14

89 CD34 cells differentiated to erythrocyte lineage vs. Neutrophils (up-regulated)

GO term	FDR	Counts at both methods	Counts at RECLU	Counts at original Paraclu
translation	1.3e-33	116	6	2
ncRNA metabolic process	1.6e-18	75	3	0
RNA processing	5.4e-15	120	1	0
cell cycle	4.1e-14	150	6	1
ribosome biogenesis	8.8e-14	47	1	0
chromosome organization	2e-13	108	0	0
protein-DNA complex assembly	2e-13	40	0	0
ribonucleoprotein complex biogenesis	2e-13	58	0	0
nucleosome assembly	2e-13	38	4	0
nucleosome organization	4e-13	40	0	0
DNA packaging	4e-13	45	0	0
cellular macromolecular complex subunit organization	8.1e-13	87	0	0
chromatin assembly	1.2e-12	38	0	0
translational elongation	9.9e-12	40	3	0
cellular macromolecular complex assembly	2.2e-11	78	0	0
DNA metabolic process	2.5e-11	106	0	0
ncRNA processing	2.8e-11	56	0	0
cell cycle process	3.4e-11	114	1	0
chromatin assembly or disassembly	9.5e-11	44	0	0
mitotic cell cycle	5.7e-10	83	6	0
M phase	1.4e-09	76	0	0
rRNA metabolic process	1.8e-09	36	0	0
macromolecular complex subunit organization	3.9e-09	128	0	0
cell cycle phase	6.5e-09	87	0	0
rRNA processing	1.4e-08	34	2	1
macromolecular complex assembly	2e-08	120	0	0
organelle fission	2e-08	58	0	0
M phase of mitotic cell cycle	2.5e-08	57	0	0
mitosis	3.9e-08	56	6	2
nuclear division	3.9e-08	56	0	0
tRNA metabolic process	7.3e-08	38	0	0
chromatin organization	6.1e-07	77	0	0
DNA replication	1.6e-06	48	3	1
cell division	1.1e-05	62	12	4
amino acid activation	0.00017	19	0	0
tRNA aminoacylation for protein translation	0.00017	19	1	0
tRNA aminoacylation	0.00017	19	0	0
response to DNA damage stimulus	0.0014	67	4	0
DNA repair	0.0015	55	10	0
cofactor metabolic process	0.0022	42	0	0
chromosome segregation	0.0025	24	0	0
mitochondrion organization	0.0036	33	5	1
DNA replication initiation	0.0078	10	2	0
negative regulation of cellular protein metabolic process	0.013	38	1	0
cellular respiration	0.021	25	1	0
negative regulation of protein metabolic process	0.031	38	0	0
RNA splicing	0.034	51	13	1
RNA splicing, via transesterification reactions	0.036	33	0	0
nuclear mRNA splicing, via spliceosome	0.036	33	7	0
RNA splicing, via transesterification reactions with bulged adenosine as nucleophile	0.036	33	0	0
mRNA metabolic process	0.036	62	3	0
regulation of cell cycle	0.037	57	1	0
tRNA processing	0.042	21	2	2
heterocycle biosynthetic process	0.048	18	0	0
sum		3113	104	15

90 CD34 cells differentiated to erythrocyte lineage vs. Neutrophils (down-regulated)

GO term	FDR	Counts at both methods	Counts at RECLU	Counts at original Paraclu
immune response	9.1e-16	110	2	4
intracellular signaling cascade	9.4e-09	144	0	0
defense response	1e-08	87	0	0
response to wounding	1.5e-07	76	0	1
inflammatory response	6.1e-07	54	2	9
leukocyte activation	2e-06	44	0	0
regulation of apoptosis	4.2e-06	97	1	1
regulation of programmed cell death	7e-06	97	0	0
regulation of cell death	8.4e-06	97	0	0
actin cytoskeleton organization	2.8e-05	40	4	0
apoptosis	4.6e-05	76	13	5
cell activation	4.6e-05	46	0	0
programmed cell death	8.6e-05	76	0	0
actin filament-based process	0.00017	40	0	0
immune effector process	0.00018	28	0	0
positive regulation of lymphocyte differentiation	0.00022	14	0	0
regulation of alpha-beta T cell activation	0.00033	14	0	0
death	0.00034	84	0	0
positive regulation of immune system process	0.00035	39	0	0
regulation of protein kinase cascade	0.00041	40	0	0
regulation of I-kappaB kinase/NF-kappaB cascade	0.00047	24	1	0
cell death	0.0005	83	3	1
positive regulation of T cell differentiation	0.00061	13	0	0
protein kinase cascade	0.00096	51	2	0
positive regulation of I-kappaB kinase/NF-kappaB cascade	0.0014	22	1	2
protein amino acid phosphorylation	0.0015	77	2	2
positive regulation of protein kinase cascade	0.0017	30	0	0
lymphocyte activation	0.0027	33	0	0
regulation of alpha-beta T cell differentiation	0.003	11	0	0
leukocyte mediated immunity	0.0033	20	0	0
positive regulation of alpha-beta T cell activation	0.0045	11	0	0
regulation of T cell differentiation	0.005	15	1	0
anti-apoptosis	0.0059	33	0	0
phosphorus metabolic process	0.006	100	0	0
phosphate metabolic process	0.006	100	0	0
regulation of phosphate metabolic process	0.007	59	0	0
regulation of phosphorus metabolic process	0.007	59	0	0
positive regulation of apoptosis	0.0076	54	3	1
negative regulation of apoptosis	0.0081	47	11	1
regulation of phosphorylation	0.0088	57	0	0
positive regulation of programmed cell death	0.0093	54	0	0
positive regulation of cell death	0.011	54	0	0
negative regulation of programmed cell death	0.012	47	0	0
positive regulation of signal transduction	0.012	41	0	0
negative regulation of cell death	0.013	47	0	0
positive regulation of cell activation	0.013	22	0	0
regulation of lymphocyte differentiation	0.015	16	0	0
positive regulation of lymphocyte activation	0.022	20	0	0
regulation of cytokine production	0.026	29	0	0
regulation of T cell activation	0.031	22	0	0
positive regulation of cell communication	0.032	43	0	0
positive regulation of alpha-beta T cell differentiation	0.034	9	0	0
positive regulation of T cell activation	0.041	17	0	0
innate immune response	0.044	24	11	10
regulation of lymphocyte activation	0.048	25	0	0
cytoskeleton organization	0.048	52	2	0
regulation of kinase activity	0.049	45	0	0
sum		2769	59	37

91 CD4+CD25-CD45RA+ naive conventional T cell vs. CD4+ T cells (up-regulated)

GO term	FDR	Counts at both methods	Counts at RECLU	Counts at original Paraclu
sum		0	0	0

92 CD4+CD25-CD45RA+ naive conventional T cell vs. CD4+ T cells (down-regulated)

GO term	FDR	Counts at both methods	Counts at RECLU	Counts at original Paraclu
sum		0	0	0

93 CD4+CD25-CD45RA+ naive conventional T cell vs. CD8+ T cells (up-regulated)

GO term	FDR	Counts at both methods	Counts at RECLU	Counts at original Paraclu
regulation of apoptosis	0.049	26	1	0
sum		26	1	0

94 CD4+CD25-CD45RA+ naive conventional T cell vs. CD8+ T cells (down-regulated)

GO term	FDR	Counts at both methods	Counts at RECLU	Counts at original Paraclu
protein kinase cascade	0.00024	37	2	0
leukocyte activation	0.00063	28	0	0
cell activation	0.0019	30	0	0
intracellular signaling cascade	0.0037	80	0	0
MAPKKK cascade	0.0089	22	2	0
immune response	0.018	50	9	1
death	0.03	51	0	0
regulation of apoptosis	0.031	55	5	0
regulation of programmed cell death	0.04	55	0	0
regulation of cell death	0.044	55	0	0
sum		463	18	1

95 CD4+CD25-CD45RA+ naive conventional T cell vs. Macrophage - monocyte derived (up-regulated)

GO term	FDR	Counts at both methods	Counts at RECLU	Counts at original Paraclu
translational elongation	2.2e-12	30	9	0
regulation of T cell activation	0.0002	22	0	0
protein-DNA complex assembly	0.00035	19	0	0
hemopoietic or lymphoid organ development	0.00037	34	0	0
nucleosome assembly	0.00053	18	7	0
chromatin assembly	0.00091	18	0	0
hemopoiesis	0.0013	31	0	0
leukocyte differentiation	0.0014	22	0	0
immune system development	0.0015	34	0	0
translation	0.0017	38	4	0
lymphocyte differentiation	0.0024	19	0	0
nucleosome organization	0.0024	18	0	0
T cell activation	0.003	21	3	0
lymphocyte activation	0.0041	27	1	0
regulation of lymphocyte activation	0.011	22	0	0
chromatin assembly or disassembly	0.013	20	0	0
positive regulation of immune system process	0.014	29	0	0
T cell differentiation	0.015	14	0	0
DNA packaging	0.015	19	0	0
chromatin organization	0.015	39	0	0
protein amino acid phosphorylation	0.031	57	5	0
regulation of T cell differentiation	0.035	12	0	0
regulation of cell activation	0.045	23	0	0
sum		586	29	0

96 CD4+CD25-CD45RA+ naive conventional T cell vs. Macrophage - monocyte derived (down-regulated)

GO term	FDR	Counts at both methods	Counts at RECLU	Counts at original Paraclu
inflammatory response	5.8e-07	62	11	2
response to wounding	2.1e-06	85	1	0
immune response	7.3e-06	101	7	2
immune effector process	3e-05	33	0	0
defense response	6e-05	90	1	0
oxidation reduction	0.00035	90	0	0
response to organic substance	0.00096	97	0	0
intracellular signaling cascade	0.0027	148	0	0
leukocyte mediated immunity	0.0059	22	0	0
membrane organization	0.0067	58	6	1
lymphocyte mediated immunity	0.014	19	0	0
regulation of organelle organization	0.019	38	0	0
sterol biosynthetic process	0.019	13	0	0
regulation of apoptosis	0.021	100	6	0
small GTPase mediated signal transduction	0.022	48	22	4
innate immune response	0.023	28	34	5
cell proliferation	0.027	62	16	5
regulation of programmed cell death	0.032	100	0	0
fatty acid metabolic process	0.036	35	2	0
regulation of cell death	0.037	100	0	0
vesicle-mediated transport	0.039	76	5	1
lipid biosynthetic process	0.047	49	0	0
actin cytoskeleton organization	0.048	38	7	2
sum		1492	118	22

97 CD4+CD25-CD45RA+ naive conventional T cell vs. Neutrophils (up-regulated)

GO term	FDR	Counts at both methods	Counts at RECLU	Counts at original Paraclu
translation	3.9e-32	115	13	0
ncRNA metabolic process	1.9e-21	80	3	0
translational elongation	1.6e-16	46	1	0
ribonucleoprotein complex biogenesis	4.2e-15	61	0	0
ribosome biogenesis	3.1e-14	48	3	0
ncRNA processing	8.1e-13	59	0	0
RNA processing	3e-11	113	5	0
rRNA metabolic process	5.5e-10	37	0	0
rRNA processing	4.1e-09	35	4	0
tRNA metabolic process	6e-09	40	0	0
nucleosome organization	3.7e-06	31	0	0
cellular macromolecular complex subunit organization	2e-05	71	0	0
nucleosome assembly	2.5e-05	28	7	1
amino acid activation	3.5e-05	20	0	0
tRNA aminoacylation	3.5e-05	20	0	0
tRNA aminoacylation for protein translation	3.5e-05	20	5	0
protein-DNA complex assembly	4.1e-05	29	0	0
chromatin assembly	5.9e-05	28	0	0
cellular macromolecular complex assembly	7.2e-05	64	0	0
mitochondrion organization	0.00018	36	4	0
chromosome organization	0.00051	84	1	0
DNA metabolic process	0.00084	86	1	0
chromatin organization	0.00099	69	0	0
DNA packaging	0.0011	31	0	0
macromolecular complex subunit organization	0.011	107	0	0
macromolecular complex assembly	0.014	101	0	0
chromatin assembly or disassembly	0.021	30	0	0
protein import	0.039	30	0	0
sum		1519	47	1

98 CD4+CD25-CD45RA+ naive conventional T cell vs. Neutrophils (down-regulated)

GO term	FDR	Counts at both methods	Counts at RECLU	Counts at original Paraclu
immune response	6e-13	94	6	4
defense response	7.8e-12	85	1	0
response to wounding	1.3e-11	77	0	0
inflammatory response	9.4e-11	56	4	4
intracellular signaling cascade	3e-07	123	0	0
protein kinase cascade	6.3e-07	53	1	0
regulation of apoptosis	2.4e-06	87	1	2
regulation of programmed cell death	3.8e-06	87	0	0
regulation of cell death	4.6e-06	87	0	0
immune effector process	5.9e-06	28	0	0
apoptosis	3.9e-05	68	9	4
positive regulation of immune system process	5.1e-05	37	0	0
programmed cell death	7.1e-05	68	0	0
protein amino acid phosphorylation	9e-05	72	3	1
regulation of cytokine production	9.2e-05	31	0	0
response to organic substance	9.7e-05	76	1	0
actin cytoskeleton organization	0.00014	35	3	0
positive regulation of transferase activity	0.0002	36	0	0
positive regulation of kinase activity	0.00024	35	0	0
negative regulation of apoptosis	0.00057	45	15	2
actin filament-based process	0.00069	35	0	0
regulation of kinase activity	0.00072	45	0	0
phosphorylation	0.00074	79	0	0
leukocyte activation	0.00076	35	0	0
negative regulation of programmed cell death	0.00084	45	0	0
regulation of transferase activity	0.00089	46	0	0
negative regulation of cell death	0.0009	45	0	0
positive regulation of protein kinase activity	0.001	33	0	0
positive regulation of response to stimulus	0.0012	34	0	0
response to bacterium	0.0013	30	0	0
response to molecule of bacterial origin	0.0014	19	0	0
leukocyte mediated immunity	0.0014	19	0	0
regulation of phosphorylation	0.0015	53	1	0
cell death	0.0016	72	1	1
cell activation	0.002	38	0	0
positive regulation of immune response	0.002	25	0	0
death	0.0021	72	1	0
regulation of phosphorus metabolic process	0.0022	54	0	0
regulation of phosphate metabolic process	0.0022	54	0	0
positive regulation of cytokine production	0.0028	19	0	0
phosphate metabolic process	0.0034	89	0	0
phosphorus metabolic process	0.0034	89	0	0
regulation of protein kinase activity	0.0046	42	1	0
anti-apoptosis	0.0051	30	0	0
response to lipopolysaccharide	0.0062	17	2	1
innate immune response	0.011	23	19	6
regulation of protein kinase cascade	0.012	33	0	0
positive regulation of apoptosis	0.02	47	3	1
positive regulation of programmed cell death	0.024	47	0	0
positive regulation of cell death	0.027	47	0	0
adaptive immune response	0.029	16	0	0
adaptive immune response based on somatic recombination of immune receptors built from immunoglobulin superfamily domains	0.029	16	0	0
regulation of I-kappaB kinase/NF-kappaB cascade	0.035	19	1	0
taxis	0.038	24	0	0
chemotaxis	0.038	24	1	1
MAPKKK cascade	0.045	26	1	1
positive regulation of cell communication	0.049	38	0	0
sum		2759	75	28

99 CD4+ T cells vs. CD8+ T cells (up-regulated)

GO term	FDR	Counts at both methods	Counts at RECLU	Counts at original Paraclu
inflammatory response	7.1e-07	12	0	0
response to wounding	9.5e-06	13	0	0
chemotaxis	0.00028	8	0	0
taxis	0.00028	8	0	0
defense response	0.00052	12	0	0
locomotory behavior	0.01	8	0	0
sum		61	0	0

100 CD4+ T cells vs. CD8+ T cells (down-regulated)

GO term	FDR	Counts at both methods	Counts at RECLU	Counts at original Paraclu
immune response	0.0012	18	2	0
sum		18	2	0

101 CD4+ T cells vs. Macrophage - monocyte derived (up-regulated)

GO term	FDR	Counts at both methods	Counts at RECLU	Counts at original Paraclu
chromatin organization	4.6e-06	52	0	0
regulation of T cell activation	3.3e-05	25	0	0
protein-DNA complex assembly	0.00075	20	0	0
regulation of lymphocyte activation	0.0009	26	0	0
nucleosome assembly	0.001	19	6	0
nucleosome organization	0.0011	20	0	0
chromatin assembly	0.0017	19	0	0
chromosome organization	0.0025	54	2	0
chromatin assembly or disassembly	0.0028	23	0	0
lymphocyte differentiation	0.0054	20	0	0
regulation of cell activation	0.0066	27	0	0
regulation of leukocyte activation	0.0079	26	0	0
T cell activation	0.0091	22	4	0
hemopoietic or lymphoid organ development	0.012	34	0	0
translational elongation	0.016	19	7	1
leukocyte differentiation	0.017	22	0	0
T cell differentiation	0.017	15	1	1
lymphocyte activation	0.024	28	1	0
hemopoiesis	0.03	31	2	0
chromatin modification	0.036	34	5	0
DNA packaging	0.037	20	1	0
immune system development	0.042	34	0	0
transcription	0.046	158	0	0
sum		748	29	2

102 CD4+ T cells vs. Macrophage - monocyte derived (down-regulated)

GO term	FDR	Counts at both methods	Counts at RECLU	Counts at original Paraclu
oxidation reduction	3.2e-09	107	0	0
inflammatory response	7.3e-05	58	8	0
response to wounding	0.00063	79	1	0
coenzyme metabolic process	0.0015	33	0	0
immune effector process	0.0023	30	0	0
cofactor metabolic process	0.0026	38	0	0
lymphocyte mediated immunity	0.0047	20	0	0
immune response	0.0088	92	7	1
leukocyte mediated immunity	0.009	22	0	0
fatty acid metabolic process	0.025	36	4	0
immunoglobulin mediated immune response	0.039	16	1	0
carboxylic acid biosynthetic process	0.046	30	0	0
organic acid biosynthetic process	0.046	30	0	0
sum		591	21	1

103 CD4+ T cells vs. Neutrophils (up-regulated)

GO term	FDR	Counts at both methods	Counts at RECLU	Counts at original Paraclu
translation	4.6e-27	98	29	0
ncRNA metabolic process	7.6e-18	68	5	0
translational elongation	8.2e-18	44	3	0
ribonucleoprotein complex biogenesis	8.8e-17	58	0	0
ribosome biogenesis	6.2e-15	45	4	0
RNA processing	6e-13	104	10	0
ncRNA processing	2.2e-12	53	0	0
rRNA metabolic process	5.5e-10	34	0	0
rRNA processing	5.3e-09	32	6	0
nucleosome assembly	1.3e-08	30	10	0
protein-DNA complex assembly	2.2e-08	31	0	0
chromatin assembly	3.6e-08	30	0	0
nucleosome organization	2.3e-07	30	0	0
cellular macromolecular complex assembly	1.1e-06	61	0	0
tRNA metabolic process	6e-06	32	0	0
cellular macromolecular complex subunit organization	1.7e-05	63	0	0
DNA packaging	2e-05	31	1	0
chromatin organization	2.6e-05	65	0	0
chromosome organization	3.8e-05	77	1	0
chromatin assembly or disassembly	0.00015	31	0	0
macromolecular complex assembly	0.001	92	0	0
macromolecular complex subunit organization	0.0053	94	0	0
mitochondrion organization	0.01	29	3	0
sum		1232	72	0

104 CD4+ T cells vs. Neutrophils (down-regulated)

GO term	FDR	Counts at both methods	Counts at RECLU	Counts at original Paraclu
inflammatory response	6.3e-15	57	4	8
defense response	4.7e-14	80	1	2
response to wounding	6.7e-14	73	0	0
immune response	6e-13	84	5	5
actin cytoskeleton organization	1.5e-06	35	0	2
actin filament-based process	8.4e-06	35	0	0
immune effector process	1.5e-05	25	0	0
apoptosis	4.4e-05	60	5	7
intracellular signaling cascade	7.4e-05	100	0	0
programmed cell death	7.5e-05	60	0	0
regulation of apoptosis	0.00011	72	0	1
regulation of programmed cell death	0.00017	72	0	0
regulation of cell death	0.00019	72	0	0
regulation of cytokine production	0.00039	27	0	0
response to bacterium	0.00039	28	0	0
response to molecule of bacterial origin	0.00052	18	0	1
protein kinase cascade	0.00072	41	0	9
cell death	0.00087	64	1	3
death	0.0011	64	0	0
leukocyte activation	0.0012	31	0	0
phosphorylation	0.0019	68	0	0
cell activation	0.0019	34	0	0
positive regulation of immune system process	0.0027	30	0	0
response to lipopolysaccharide	0.0032	16	2	1
protein amino acid phosphorylation	0.0032	59	1	3
cytoskeleton organization	0.0032	44	0	0
response to organic substance	0.0042	62	0	0
negative regulation of apoptosis	0.0044	38	9	6
positive regulation of kinase activity	0.0045	29	0	0
negative regulation of programmed cell death	0.0062	38	0	0
negative regulation of cell death	0.0066	38	0	0
positive regulation of response to stimulus	0.0069	29	0	0
taxis	0.0069	23	0	0
chemotaxis	0.0069	23	0	3
innate immune response	0.0084	21	7	13
positive regulation of transferase activity	0.0095	29	1	0
cytokine production	0.0095	12	0	2
regulation of tumor necrosis factor production	0.012	10	0	0
leukocyte mediated immunity	0.013	16	0	0
adaptive immune response based on somatic recombination of immune receptors built from immunoglobulin superfamily domains	0.016	15	0	0
adaptive immune response	0.016	15	0	1
positive regulation of immune response	0.018	21	0	0
regulation of protein kinase cascade	0.019	29	0	0
positive regulation of cytokine production	0.024	16	0	0
lymphocyte mediated immunity	0.027	14	0	0
phosphate metabolic process	0.032	74	0	0
phosphorus metabolic process	0.032	74	0	0
sum		1975	36	67

105 CD8+ T cells vs. Macrophage - monocyte derived (up-regulated)

GO term	FDR	Counts at both methods	Counts at RECLU	Counts at original Paraclu
chromatin organization	0.00066	50	0	0
lymphocyte differentiation	0.0014	22	0	0
hemopoietic or lymphoid organ development	0.0019	38	0	0
protein amino acid phosphorylation	0.002	73	5	1
T cell differentiation	0.002	17	2	0
T cell activation	0.0032	24	4	0
hemopoiesis	0.004	35	1	0
lymphocyte activation	0.0061	31	0	0
leukocyte differentiation	0.0064	24	0	0
leukocyte activation	0.007	35	0	0
immune system development	0.0081	38	0	0
chromatin modification	0.017	37	1	0
sum		424	13	1

106 CD8+ T cells vs. Macrophage - monocyte derived (down-regulated)

GO term	FDR	Counts at both methods	Counts at RECLU	Counts at original Paraclu
oxidation reduction	1.8e-09	111	0	0
inflammatory response	1.3e-06	64	7	0
response to wounding	2.5e-06	89	1	0
immune response	0.00027	101	5	0
cofactor metabolic process	0.00037	41	0	0
coenzyme metabolic process	0.00042	35	0	0
regulation of apoptosis	0.0012	111	9	0
immune effector process	0.0018	31	0	0
regulation of programmed cell death	0.002	111	0	0
regulation of cell death	0.0024	111	0	0
defense response	0.0027	89	1	0
leukocyte mediated immunity	0.018	22	0	0
fatty acid metabolic process	0.027	37	4	0
intracellular signaling cascade	0.034	151	0	0
lymphocyte mediated immunity	0.036	19	0	0
sum		1123	27	0

107 CD8+ T cells vs. Neutrophils (up-regulated)

GO term	FDR	Counts at both methods	Counts at RECLU	Counts at original Paraclu
translation	1.7e-23	100	21	0
ncRNA metabolic process	1.9e-16	71	3	0
ribonucleoprotein complex biogenesis	2.9e-15	60	0	0
translational elongation	9.4e-14	42	2	0
ribosome biogenesis	2e-13	46	4	0
RNA processing	1.6e-12	113	5	0
ncRNA processing	1.8e-10	54	0	0
rRNA metabolic process	5.3e-09	35	0	0
rRNA processing	4e-08	33	9	0
tRNA metabolic process	1.2e-05	34	0	0
protein-DNA complex assembly	1.7e-05	29	0	0
nucleosome assembly	4.9e-05	27	10	0
cellular macromolecular complex assembly	9.2e-05	62	0	0
chromatin assembly	0.00011	27	0	0
cellular macromolecular complex subunit organization	0.00011	67	0	0
nucleosome organization	0.00012	28	0	0
mitochondrion organization	0.0022	33	2	0
chromatin organization	0.0091	64	0	0
chromosome organization	0.011	77	1	0
ribosomal large subunit biogenesis	0.011	8	1	0
macromolecular complex assembly	0.013	98	0	0
macromolecular complex subunit organization	0.015	103	0	0
DNA packaging	0.016	28	1	0
tRNA aminoacylation	0.021	16	0	0
amino acid activation	0.021	16	0	0
tRNA aminoacylation for protein translation	0.021	16	6	0
chromatin assembly or disassembly	0.028	29	0	0
sum		1316	65	0

108 CD8+ T cells vs. Neutrophils (down-regulated)

GO term	FDR	Counts at both methods	Counts at RECLU	Counts at original Paraclu
response to wounding	2e-13	79	0	1
inflammatory response	6e-13	59	7	3
defense response	5.8e-12	84	2	0
immune response	8.4e-12	90	5	2
intracellular signaling cascade	2.1e-06	118	0	0
immune effector process	1.6e-05	27	0	0
actin cytoskeleton organization	2.3e-05	36	2	0
protein kinase cascade	5.4e-05	48	2	0
actin filament-based process	0.00012	36	0	0
apoptosis	0.00019	65	10	3
programmed cell death	0.00032	65	0	0
regulation of apoptosis	0.00034	79	0	1
leukocyte activation	0.00043	35	0	0
regulation of programmed cell death	0.00051	79	0	0
regulation of cell death	0.00059	79	0	0
response to bacterium	0.00079	30	0	0
response to lipopolysaccharide	0.00088	18	2	0
response to molecule of bacterial origin	0.00096	19	0	0
cell activation	0.0011	38	0	0
protein amino acid phosphorylation	0.0016	67	2	0
phosphorylation	0.0022	76	0	0
regulation of cytokine production	0.0023	28	0	0
response to organic substance	0.0031	70	0	0
positive regulation of kinase activity	0.0039	32	0	0
leukocyte mediated immunity	0.0046	18	0	0
positive regulation of cytokine production	0.0088	18	0	0
positive regulation of transferase activity	0.0089	32	1	0
negative regulation of apoptosis	0.012	41	16	2
positive regulation of protein kinase activity	0.015	30	0	0
negative regulation of programmed cell death	0.016	41	1	0
positive regulation of response to stimulus	0.017	31	0	0
negative regulation of cell death	0.017	41	0	0
positive regulation of immune system process	0.02	31	0	0
cell death	0.021	67	4	0
positive regulation of apoptosis	0.023	46	3	0
phosphate metabolic process	0.025	84	0	0
phosphorus metabolic process	0.025	84	0	0
innate immune response	0.025	22	18	6
chemotaxis	0.026	24	1	2
taxis	0.026	24	0	0
death	0.026	67	0	0
positive regulation of programmed cell death	0.028	46	0	0
positive regulation of cell death	0.031	46	0	0
cytoskeleton organization	0.033	46	0	0
regulation of kinase activity	0.034	40	0	0
cytokine production	0.042	12	1	0
regulation of tumor necrosis factor production	0.044	10	0	0
regulation of protein kinase cascade	0.05	31	0	0
sum		2289	77	20

109 Macrophage - monocyte derived vs. Neutrophils (up-regulated)

GO term	FDR	Counts at both methods	Counts at RECLU	Counts at original Paraclu
translation	2.8e-20	108	11	0
ncRNA metabolic process	3.3e-16	80	3	0
ribosome biogenesis	3.1e-10	47	1	0
ribonucleoprotein complex biogenesis	6.5e-10	59	0	0
ncRNA processing	1.1e-09	60	0	0
tRNA metabolic process	5.4e-07	41	0	0
rRNA metabolic process	7.2e-07	36	0	0
oxidation reduction	1.9e-06	129	1	0
translational elongation	3.5e-06	36	4	0
rRNA processing	3.7e-06	34	5	0
cellular macromolecular complex subunit organization	2e-05	81	0	0
cellular macromolecular complex assembly	3.1e-05	74	0	0
cofactor metabolic process	5.7e-05	52	1	0
RNA processing	9.3e-05	109	5	1
mitochondrion organization	0.00027	40	3	0
coenzyme metabolic process	0.00063	42	0	0
tRNA aminoacylation for protein translation	0.00079	20	4	0
amino acid activation	0.00079	20	0	0
tRNA aminoacylation	0.00079	20	0	0
DNA metabolic process	0.001	99	1	0
macromolecular complex subunit organization	0.0012	129	0	0
macromolecular complex assembly	0.0015	122	0	0
cellular respiration	0.0029	30	0	0
cell cycle	0.0071	135	11	0
intracellular protein transport	0.039	73	12	0
cell cycle process	0.045	101	0	0
sum		1777	62	1

110 Macrophage - monocyte derived vs. Neutrophils (down-regulated)

GO term	FDR	Counts at both methods	Counts at RECLU	Counts at original Paraclu
immune response	2e-08	76	7	3
defense response	2.6e-07	68	2	0
inflammatory response	1.9e-06	44	7	2
protein amino acid phosphorylation	4.4e-05	66	0	1
response to wounding	6.9e-05	56	0	0
intracellular signaling cascade	0.00014	102	0	0
phosphorylation	0.0017	70	0	0
leukocyte activation	0.0027	31	0	0
phosphate metabolic process	0.003	80	0	0
phosphorus metabolic process	0.003	80	0	0
protein kinase cascade	0.0048	40	1	0
apoptosis	0.0092	55	7	1
programmed cell death	0.014	55	0	0
regulation of apoptosis	0.016	67	3	1
regulation of programmed cell death	0.022	67	0	0
regulation of cell death	0.025	67	0	0
regulation of cytokine production	0.029	24	0	0
cell activation	0.031	32	0	0
sum		1080	27	8
